# The polyglutamine protein ataxin-3 enables normal growth under heat shock conditions in the methylotrophic yeast *Pichia pastoris*

**DOI:** 10.1038/s41598-017-13814-1

**Published:** 2017-10-17

**Authors:** Marcella Bonanomi, Valentina Roffia, Antonella De Palma, Alessio Lombardi, Francesco Antonio Aprile, Cristina Visentin, Paolo Tortora, Pierluigi Mauri, Maria Elena Regonesi

**Affiliations:** 1 Department of Biotechnology and Biosciences, University of Milano-Bicocca, 20126 Milan, Italy; 2 SYSBIO.IT, Centre of Systems Biology, 20126 Milano, Italy; 3 Institute for Biomedical Technologies, National Research Council, 20090 Milan, Italy; 4 Department of Chemistry, University of Cambridge, Cambridge, CB2 1EW United Kingdom; 5 Milan Center of Neuroscience (NeuroMI), 20126 Milano, Italy

## Abstract

The protein ataxin-3 carries a polyglutamine stretch close to the C-terminus that triggers a neurodegenerative disease in humans when its length exceeds a critical threshold. A role as a transcriptional regulator but also as a ubiquitin hydrolase has been proposed for this protein. Here, we report that, when expressed in the yeast *Pichia pastoris*, full-length ataxin-3 enabled almost normal growth at 37 °C, well above the physiological optimum of 30 °C. The N-terminal Josephin domain (JD) was also effective but significantly less, whereas catalytically inactive JD was completely ineffective. Based on MudPIT proteomic analysis, we observed that the strain expressing full-length, functional ataxin-3 displayed persistent upregulation of enzymes involved in mitochondrial energy metabolism during growth at 37 °C compared with the strain transformed with the empty vector. Concurrently, in the transformed strain intracellular ATP levels at 37 °C were even higher than normal ones at 30 °C. Elevated ATP was also paralleled by upregulation of enzymes involved in both protein biosynthesis and biosynthetic pathways, as well as of several stress-induced proteins. A similar pattern was observed when comparing a strain expressing JD with another expressing its catalytically inactive counterpart. We suggest that such effects mostly result from mechanisms of transcriptional regulation.

## Introduction

Ataxin-3 (ATX3) is one among several proteins containing stretches of consecutive glutamines that are responsible for different, albeit related, neurodegenerative diseases in humans, when their size exceeds a critical threshold^1–3^. ATX3 triggers the Machado-Joseph disease, an autosomal dominantly inherited neurodegenerative disorder, also referred to as spinocerebellar ataxia type-3. Protein variants carrying polyglutamine (polyQ) stretches whose length exceeds a critical threshold of about 50 consecutive residues, lead to protein misfolding and aggregation into large intracellular inclusions, cytotoxicity and finally dysfunction and demise of specific neurons^4,5^.

ATX3 consists of a structured globular N-terminal domain, the Josephin domain (JD), followed by a disordered C-terminal tail containing the polyQ stretch, close to the C-terminus^4,6,7^. Different physiological roles have been proposed for this protein. Besides a possible role as a modulator of transcription^8–10^, plenty of evidence supports its role as a cysteine protease capable of cleaving isopeptide bonds between ubiquitin (Ub) monomers^11–13^. In fact, the JD has the catalytic triad found in these proteases, the residue Cys14 being the one directly involved in catalysis, and displays ubiquitin hydrolase activity even in isolation^11^. Furthermore, the C-terminal, disordered domain has two or three ubiquitin-interacting motifs (UIMs) depending on the splice variant^14^. However, the mechanisms by which ATX3 binds and cleaves polyUb chains is complex and far from being fully understood. Actually, the protein binds polyUb chains containing four or more ubiquitins, both K48 and K63-linked. Also, it cleaves both types of linkages, yet displays a preference, at least *in vitro*, for protein substrates carrying mixed-linkage and K63-linkage chains^12^. In this process, the UIMs regulate the specificity of the cleavage by restricting what can be cleaved by the N-terminal protease domain.

Regarding ATX3’s role(s) in sorting proteins destined for degradation, an intricate and not yet completely understood picture has emerged from recent investigations^15^. This protein interacts with various components of the proteasome, such as Rad23 and valosin-containing protein (VCP), which are involved in the ubiquitin-proteasome degradation pathway and in the uptake of endoplasmic-reticulum-associated protein degradation (ERAD) substrates^16^. Moreover, it seems to play a role in sorting misfolded/aggregated proteins to the aggresomes when proteasomes are overloaded or their function compromised. Available data suggest that ATX3 fulfills this task as a part of a multiprotein complex wherein, along with K63-ubiquinated aggregates, HDAC6 is also included. The latter recruits them to dynein motor complexes that subsequently transport the aggregated cargo to the aggresomes via microtubules^17–21^. On the whole, this picture highlights ATX3 as a sorting device of proteins committed to degradation, including those residing in the ER, by destining them for either the proteasome or the aggresome.

Aiming at clarifying ATX3’s physiological role(s) and mechanisms of toxicity, in previous investigations we expressed this protein in different animal and cellular models, both prokaryotic and eukaryotic^22–25^. Still to provide insight into normal and pathological ATX3 behavior, we recently expressed different variants thereof in *Pichia pastoris*. This methylotrophic yeast is one of the most commonly used host organisms for the production of recombinant proteins, due to its ability to grow at high cell density in inexpensive media, to express foreign genes and, no less important, to glycosylate the corresponding proteins with human-like pattern^26^. Notably, this microorganism does not have an ATX3 counterpart. However, during this investigation we surprisingly observed that expression of normal full-length ATX3 conferred on the yeast a remarkable heat resistance, thus enabling it to grow at 37 °C at almost normal rates, i.e., those observed at 30 °C. This prompted us to undertake the present study to clarify the molecular and cellular mechanism(s) underlying this adaptation. To this end, we took advantage of Multidimensional Protein Identification Technology (MudPIT)^27–29^, a gel-free proteomic approach that has been successfully adopted to characterize ATX3 functions and interactions^19,30^. Besides extending our understanding of ATX3’s physiological roles, our finding might also help enlarge the potential of *P*. *pastoris* as a protein expression system.

## Experimental procedures

### Yeast strains, plasmids and media

Experiments were carried out in X-33 *Pichia pastoris* wild type strain (Invitrogen, CA, USA). The cDNA-encoding human isoform 1 AT3Q26 (GI: 833928, entry uniprot: P54252) was digested with *XhoI* and *SfuI* restriction enzymes and subcloned into the constitutive expression vector pGAPZ B (Invitrogen, CA, USA). JD was obtained by inserting two stop codons into the wild-type human AT3 gene downstream of the triplet encoding residue 182. The JD-C14A was obtained using the following forward primer: 5′-CAAGAAGGCTCACTTgcTGCTCAACATTGCC-3′. Transformation of yeast was performed by electroporation^31^ with 20 μg of *DraI*-linearized plasmid and plated onto YPDS (1% yeast extract, 2% peptone, 2% glucose, 1 M sorbitol) plates containing 100 μg/ml Zeocin™ (Zeo). Yeast cells transformed with the pGAPZ B empty vector were used as a control. Transformed *P*. *pastoris* was grown in YPD medium (1% yeast extract, 2% peptone, 2% glucose) containing Zeocin™ (YPD-Zeo).

### SDS-PAGE and Western blot analysis

Cells from single yeast colonies were grown at different times at 30 °C in YPD-Zeo medium. Culture aliquots corresponding to 1 ml at 10 optical density units at 600 nm (OD_600_) were collected after centrifugation for 10 min at 6000 × g, washed with 500 μl 20% trichloroacetic acid (TCA), harvested and resuspended in 100 μl 20% TCA. Cells were broken using glass beads (0.5 mm diameter) by vortexing for 5 min. 50 μl 5% TCA was added to each sample and supernatants centrifuged at 960 × g for 10 min. Pellets were resuspended in 50 μl of sample buffer, neutralized with 50 μl 1 M Tris base and subjected to SDS PAGE. ATX3 expression levels in cultures grown at 30 °C and 37 °C were revealed by Western blotting using an anti-ATX3 Z46 rabbit polyclonal antibody, as previously described^22^. Briefly, proteins were transferred on a PVDF membrane by using a Mighty Small Transphor Blotting System (GE Healthcare Europe, Milan, Italy). Membranes were incubated in blocking solution (5% skim milk in PBS) for 1 h at room temperature, then probed 1 h at room temperature with antibody at a 1∶5000 dilution in blocking solution. After incubation, membranes were washed thrice in 0.05% Tween in PBS for 15 min each time, and subsequently incubated for 1 h at room temperature in donkey anti-rabbit IRDye 800CW secondary antibody (LI-COR Biosciences, Lincoln, NE, USA) at 1∶15000 dilution. Fluorescence was scanned at 800 nm with Odyssey Fc System (LI-COR Biosciences, Lincoln, NE, USA) and analyzed with the Image Studio software (LI-COR Biosciences, Lincoln, NE, USA). As a loading control, anti-PSTAIR antibody (Abcam, Cambridge, UK) at a 1:4000 dilution in blocking solution was used. In preliminary experiments, different expression levels were detected in different transformation clones. Thus, clones displaying similar expressions of the respective proteins were selected. To provide an independent validation of the proteomic results, two proteins displaying a Differential Average (Dave) value of 2.0 in MudPIT analysis were also quantified by Western blotting. 5 μl of proteins extracts from transformed and untransformed *Pichia* strains grown at 37 °C were subjected to SDS-PAGE and immunodecorated with either anti-phosphoglycerate kinase (Antibodies-online.com) or anti-glyoxalase I (MyBioSource.com) antibodies at a 1:1000 dilution as described above. As a secondary antibodies, anti-rabbit IRDye 800CW (LI-COR Biosciences, Lincoln, NE, USA) was used at a 1∶15000 dilution. As a loading control, anti-PSTAIR antibody (Abcam, Cambridge, UK) at a 1:4000 dilution in blocking solution was used.

### Growth assays

Yeast cells were grown overnight at 30 °C under constant shaking at 160 rpm in YPD-Zeo medium. For the spotting assays, cultures were then diluted to an OD_600_ of 1. The resulting cultures were further diluted (1, 0.5, 0.1, 0.05 fold), spotted onto YPD-Zeo plates and incubated for 3 days at 30 °C or 37 °C. For the heat shock assay, cultures at OD_600_ of 1 were pre-treated for 30 min at 48 °C. Then, they were diluted and spotted as above, and incubated for 3 days at 30 °C. To trace growth profiles in liquid medium, cultures were diluted to an OD_600_ of 0.1 in YPD-Zeo medium, then incubated at 30 °C or 37 °C under constant shaking at 160 rpm. Growth under limited anaerobic conditions was performed at both 30 °C and 37 °C under shaking at 160 rpm in sealed Falcon™ 15-ml conical centrifuge tubes, each containing 14 ml of growth medium. Growth rates were measured by monitoring the OD_600_ at different times.

### Glucose and ethanol assays

The control strain (transformed with the empty vector) and the ATX3Q26-expressing strain were grown at 30 °C overnight, diluted to an OD_600_ of 0.1 and incubated under constant shaking at 37 °C for 16, 24 or 48 h. Aliquots of the culture medium at the different growth times were collected after centrifugation for 10 min at 6000 × g. Glucose and ethanol content in the supernatants was determined by HPLC analyses as reported^32^.

### ATP assay

Cells were grown overnight at 30 °C under constant shaking at 160 rpm in YPD-Zeo medium. Cultures were diluted to an OD_600_ of 0.1 in YPD-Zeo medium, then incubated at 30 °C or 37 °C under shaking. At different incubation times, 10 OD_600_ of cells were collected by centrifugation. Cells were resuspended in 400 μl of 5% TCA and broken by vortexing for 3 min with glass beads (0.5 mm diameter). Cell debris was pelleted and the supernatants neutralized with 1.6 ml 1 M Tris acetate, pH 7.75. Neutralized samples were then 100-fold diluted with ATP-free water. ATP concentration was measured using the ENLITEN^®^ ATP Assay System Bioluminescence Detection Kit (Promega, Madison, WI, USA). Briefly, 100 μl of the diluted-supernatants was added to 100 μl of the luciferin/luciferase reagent. Luminescence intensity was measured at 25 °C using a Lumat LB 9507 luminometer (Berthold Technologies, Bad Wildbad, Germany). ATP concentration was determined using a standard curve and expressed as nmol ATP/OD of cells.

### Sample preparation for MudPIT analysis

The control strain and the ATX3Q26-expressing strain were grown at 30 °C overnight, diluted to OD_600_ of 0.1 and incubated under constant shaking at 37 °C for 16, 24 or 48 h. 100 ml-cultures were collected and resuspended in 200 mM ammonium bicarbonate, pH 8.9. Cells were broken using glass beads by vortexing five times for 1 min with intervals of 1 min on ice. Cell debris was pelleted and protein concentration of the supernatants determined by the Bradford assay (Coomassie Plus Protein Assay Reagent, Thermo Scientific, Rockford, IL, USA). 1 μg of trypsin was added to 50 μg of the different protein extracts. After an overnight digestion at 37 °C, the reaction was stopped by adjusting the pH to 2.0 by addition of 1 μl of trifluoroacetic acid. The tryptic digest mixture was desalted using a PepClean^TM^ C-18 spin column (Pierce, Il, USA) and resuspended in 0.1% formic acid (Sigma-Aldrich Inc., MO, USA) in deionized water (Millipore, MA, USA).

### MudPIT analysis

Trypsin-digested samples were analyzed by means of two-dimensional micro liquid chromatography coupled to tandem mass spectrometer (2DC-MS/MS, also referred to as Multidimensional Protein Identification Technology, MudPIT) (Thermo Fisher, CA, USA). 8 µl of the digested peptide mixture was loaded by means of an Autosampler Thermo MicroAS (Thermo Fisher Scientific, CA, USA), onto a strong cation exchange column (PoliLC column, 0.33 mm d.i. ×100 mm, 5 µm, PolyLC^INC^, Columbia, MD, USA) and eluted stepwise with an eight-step ammonium chloride concentration gradient (0, 40, 60, 100, 120, 150, 400 and 700 mM). Fractions were captured in peptide traps (Vydac Zorbax 300SB C18, 0.15mm i.d. ×100 mm, 5 μm, Agilent Technologies, Germany) for concentration and desalting prior to final separation. Each salt step was loaded onto a reversed phase C18 column (Biobasic-18, 0.18 mm i.d. ×100 mm, 5 μm, Thermo Fisher Scientific, CA, USA) and separated with an acetonitrile (Sigma-Aldrich Inc., MO, USA) gradient (eluent A, 0.1% formic acid in water, eluent B, 0.1% formic acid in acetonitrile). The gradient profile was started at 5% eluent B for 3 min, 15–40% eluent B for 42 min, 40–95% eluent B for 8 min and finally ramped to 95% eluent B for 4 min, at a flow-rate of 1 µl/min. The peptides eluted from the C18 column were directly analyzed with LTQ Orbitrap^XL^ ETD^TM^ (Thermo Fisher Scientific, CA, USA), equipped with a nano-Electrospray Ionization (ESI) spray ion source. The spray capillary voltage was set at 2.5 kV, while the ion transfer capillary temperature was held at 220 °C. For each step of peptide elution from C18 column, full mass spectrometry (MS) spectra were recorded over a 400–1600 m/z range in positive ion mode, followed by five MS/MS events sequentially generated in a data-dependent manner on the first, second, third, fourth and fifth most intense ions selected from the full MS spectrum, using a dynamic exclusion for MS/MS analysis. In particular, MS/MS scans were acquired setting a Normalized Collision Energy of 35% on the precursor ion. A total of 16 LC-MS/MS runs were performed, representing the four conditions examined in replicates (30 °C at 48 h, 37 °C at 16 h, 24 h and 48 h) for the ATX3-transformed and control *P*. *pastoris* strains.

### Data handling

All data were searched using SEQUEST algorithm contained in the 3.3.1 BioWorks version software (University of Washington, licensed to ThermoFinnigan Corp., CA, US). Experimental MS/MS spectra were correlated to tryptic peptide sequences by comparison with theoretical mass spectra obtained by *in silico* digestion of the *Komagataella pastoris* protein database (6521 entries), downloaded on January 2013 from the National Centre for Biotechnology Information (NCBI) website (www.ncbi.nlm.nih.gov). This allowed the identification of peptide sequences and related proteins. The confidence of protein identification depends on the stringency of the identification of the peptide sequence and peptide matching. For this reason, a high stringency was guaranteed, in particular when using data from a single peptide. More specifically, the peptide mass search tolerance was set to 1.00 Da, the precursor on tolerance was set to 50 ppm and the intensity threshold was set to 100. Moreover, the searches were performed with no-enzyme, mass measurement tolerance levels of 2.00 Atomic Mass Units (AMU) for peptides and 1.00 AMU for MS/MS ions. Finally, to assign a final score to proteins, the SEQUEST output data were filtered setting the peptide probability to 1 × 10^−3^, minimum Xcorr of 1.5, 2.0, 2.5 and 3.0 for single, double, triple and quadruple-charged ions, respectively, and consensus score higher than 10. The output data obtained from SEQUEST software, i.e., Spectral count (total number of spectra identified for each protein) and Score (confidence index derived from the integration of several Sequest search engine variables)^33^ were compared with an in-house algorithm, Multidimensional Algorithm Protein Map (MAProMa)^28^.

### Label-free differential analysis

Differential analysis was performed by comparing SEQUEST Score values of each distinct protein identified in both technical replicates for all conditions. Differentially expressed proteins were compared using DAve and Differential Coefficient Index (DCI) algorithm in the MAProMa software. DAve is an index of the relative ratio between two compared protein lists and was defined as (X − Y)/(X + Y) × 0.5, whereas DCI is an index used to evaluate the confidence of DAve and was defined as (X + Y)/(X − Y)/2, where X and Y represent the SEQUEST SCORE values in two samples. Most confident up-represented proteins showed a DAve ≥ +0.4 and a DCI ≥ +400; most confident down-represented proteins showed a DAve ≤ −0.4 and DCI ≤ −400.

## Results

This study was initially undertaken to investigate possible toxic effects of ATX3 variants in *P*. *pastoris* used as a model organism. To do so, we used the yeast strain X-33, which enables constitutive expression of the proteins of interest by homologous recombination at the GAPDH locus. However, during these investigations we surprisingly observed that cells transformed with either full-length ATX3 or the JD in isolation could grow at 37 °C, a temperature well above the physiological value for this organism, i.e., 30 °C^26^, and capable of inducing heat shock in wild-type strains.

Thus, we decided to analyze in detail the effects of the expression of some ATX3 constructs on yeast growth, aiming ultimately at clarifying the cellular mechanisms responsible for the increased heat resistance. For this purpose, we investigated yeast strains expressing: i) a wild-type ATX3 carrying a non-pathogenic expansion of 26 consecutive glutamines (ATX3Q26); ii) the Josephin domain in isolation (JD) and iii) the JD carrying the C14A mutation (JD-C14A), which abolishes ubiquitin hydrolase activity;^11,30,34^ iv) a control strain, transformed with the empty vector. Expression analysis performed by Western blot showed that the three ATX3 variants were expressed at similar levels at both 30 °C and 37 °C (Fig. S1). In the case of JD-C14A, a doublet band was consistently detected at both temperatures and the earliest time. As a possible explanation, we speculate that ubiquitin may protect from proteolytic attack and that the mutant interacts more weakly with ubiquitin than the wild type.

**Figure 1:**
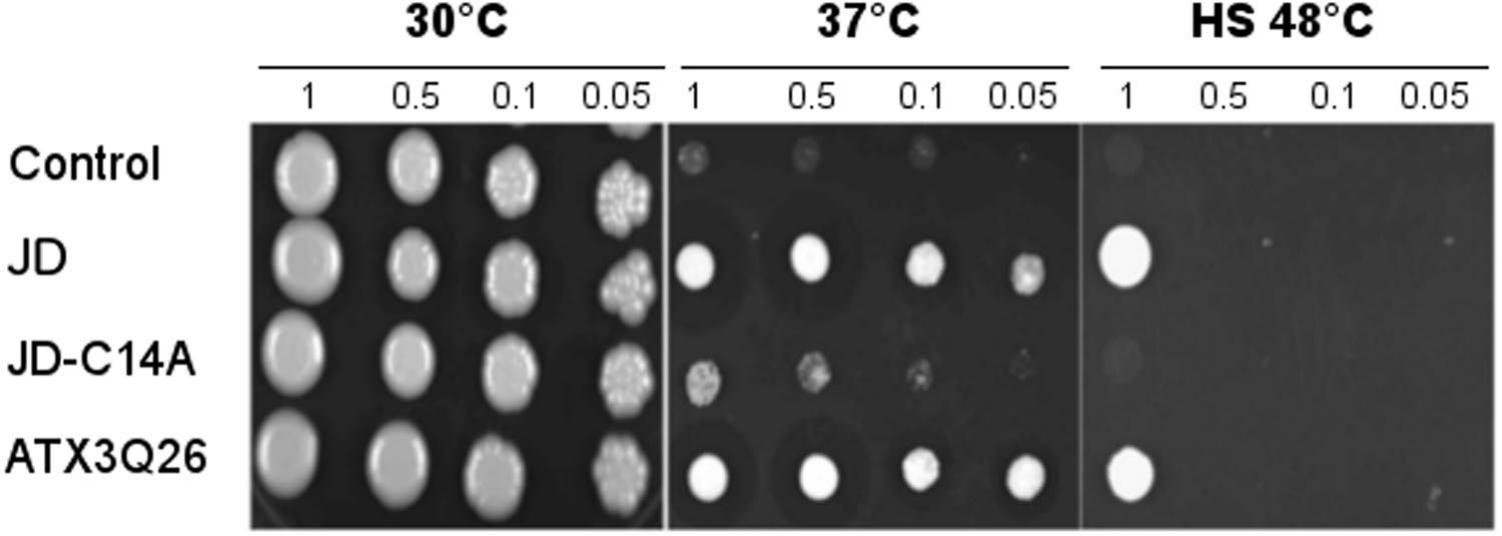
Spotting assay of ATX3 variants. After an overnight growth at 30 °C, cultures were diluted as indicated, spotted onto YPD-Zeo plates and incubated for 3 days at 30 °C or 37 °C. For the heat shock assay (HS), the cultures were treated for 30 min at 48 °C, spotted as above, then incubated at 30 °C.

We then performed spot assays at 30 °C, 37 °C and 48 °C (Fig. 1). As expected, at 30 °C significant growth was observed at any dilution. In contrast, at 37 °C the control strain did not display any appreciable growth even at the lowest dilution, whereas both ATX3Q26 and JD underwent significant cell proliferation even at the highest dilution (i.e., 20 fold). We also detected a residual growth capacity sustained by ATX3Q26 and JD at 48 °C (Fig. 1). Remarkably, the JD-C14A mutant did not rescue the proliferative capacity. As our previous data show that the mutation does not bring about appreciable structural alterations of the protein^30^, this strongly substantiates the idea of a direct involvement of the catalytic Cys14, responsible for ubiquitin hydrolase activity, in sustaining cell growth at elevated temperature.

**Figure 2:**
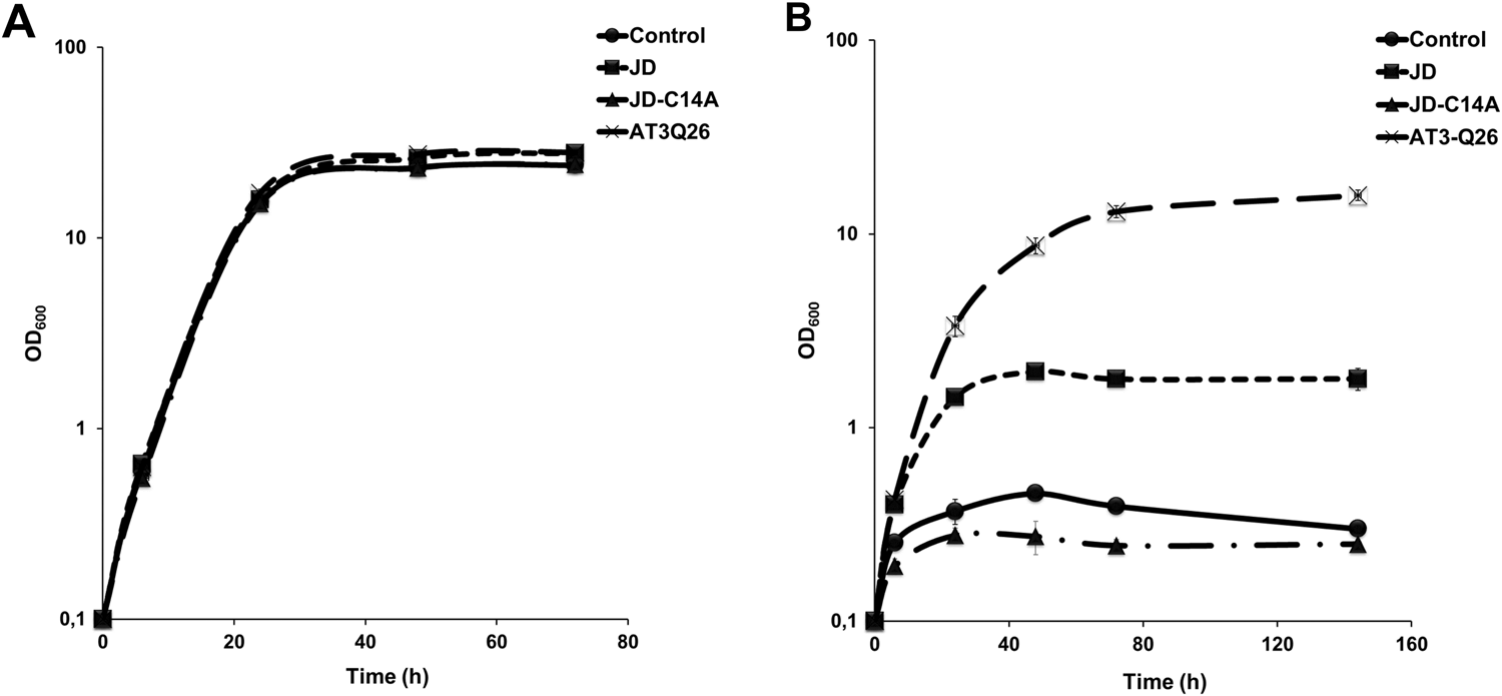
Growth curves of control strain (dots), or strains expressing JD (squares), JD-C14A (triangles) and ATX3Q26 (crosses) at 30 °C (**A**) or 37 °C (**B**). Cultures were grown in YPD-Zeo medium under constant shaking and growth profiles monitored. Error bars represent standard deviations and are derived from at least three independent replicates. In all cases, they are smaller than the symbols used.

We further analyzed the proliferative capacity of the strains under investigation by monitoring their growth in liquid medium at both 30 °C and 37 °C (Fig. 2). Whereas at 30 °C all of them underwent normal growth, with no significant difference among each other, at 37 °C the strain expressing ATX3Q26 was the only one capable of growth comparable with, although slightly lower than that of the control strain at 30 °C. Only scanty, albeit detectable proliferative capacity at 37 °C was displayed by the strain expressing the JD, which points to a role for also the disordered, C-terminal domain of ATX3 in supporting cell proliferation.

These findings prompted us to perform a proteomic investigation with the expectation that it could yield information about the cellular mechanisms responsible for cell growth at high temperature. In particular, we performed in parallel two comparative proteomic analyses: one on the strain expressing the full-length, functional ATX3, versus the control strain; the other on the JD-transformed strain versus the one carrying its non-functional counterpart, i.e. JD-C14A. Samples were analyzed at different temperatures and growth times. At 30 °C and 48 h of growth, corresponding to the stationary phase, we collected samples of full-length ATX3 and control strains. At 37 °C and 48 h, corresponding to the pre-stationary phase, we collected all four strains. Indeed, proteome analysis at the highest temperature should provide a deeper insight into the cellular adaptations underlying the observed thermoresistance. To achieve a more comprehensive view of such effects, we also analyzed ATX3Q26 and control strains at 37 °C and the earliest growth times (16 h and 24 h). Proteomic analyses were performed using an innovative MudPIT (Multidimensional Protein Identification Technology)-based platform, by which we were able to identify a total of 1078 distinct proteins. Their complete list is reported in supplemental Table S1. In addition, we demonstrated a good repeatability of our proteomic approach by plotting the Spectral Count (SpC) values of replicate analyses, which showed linear correlation (R^2^ value) and slope (y) close to theoretical value of 1 (Fig. S2). These results allowed us to align protein lists by means of the MAProMa (Multidimensional Algorithm Protein Map) Software for subsequent label-free investigations^28^. Using MAProMa, we plotted the identified proteins on a 2D virtual map, according to their theoretical Molecular Weight (MW) and Isoelectric point (pI) (Fig. S3). Furthermore, thanks to this software we made a pairwise comparison of the proteins identified under the four experimental conditions analyzed, obtaining an estimation of their relative abundance. Starting from the average Score value assigned to each protein in each list, MAProMa allowed us to calculate differentially expressed proteins by means of the parameters DAve (ranging from −2 to +2) and DCI (ranging from −∞ to ∞), which represent the ratio and the confidence in differential expression, respectively, of a protein between two samples.

By taking advantage of this proteomic approach, we first compared the *P*. *pastoris* strain expressing the full-length ATX3Q26 versus the control strain, when growing at 30 °C for 48 h. We found very few proteins differentially expressed (eight were up-regulated in the ATX3Q26-strain, whereas three were up-regulated in the control) (Table S2). Instead, interestingly, in the comparison between 30 °C and 37 °C as many as 93 proteins in the ATX3Q26-strain showed a differential expression pattern (Table S3). These proteins were stratified as follows: 15 up-regulated at 30 °C, among which 3 were exclusive; 78 up-regulated at 37 °C (among these, 47 were exclusive). Mainly, the proteins preferentially expressed at 37 °C were involved in either metabolic pathways (biosynthesis, ethanol assimilation, mitochondrial energy metabolism) or stress protection.

**Figure 3:**
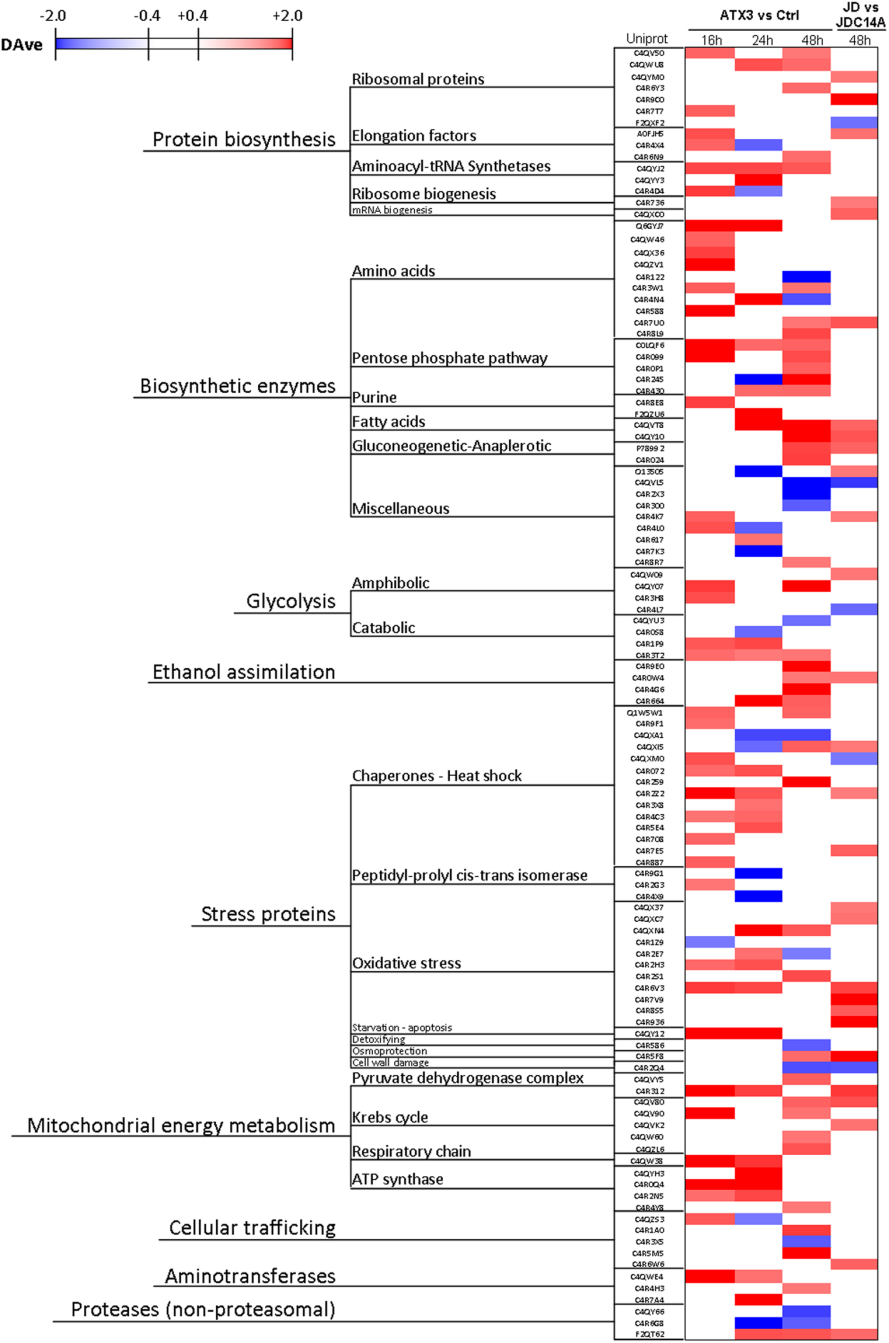
Differential changes in *P*. *pastoris* proteome as determined by MudPIT analysis during the growth at 37 °C. Differential expression of the proteins was determined at the indicated growth times using the MAProMa comparison between the ATX3-trasformed versus the control *P*. *pastoris* strain, and JD-transformed versus JD-C14A-transformed strain, respectively. Each protein was identified by its Entry Uniprot (See Supplementary Table S4 for the corresponding Gene name and complete reference). For each growth time, a color code was assigned that represents the respective DAve value. The relevant chromatic scale (reported in figure) ranges from −2.00 to −0.40 (dark blue to white) and from +0.40 to +2.00 (white to dark red). Proteins were primarily grouped according to their molecular function and secondarily by their increasing GI Accession numbers. The complete list of the proteins presented was extracted from the differential lists in Supplementary Table 4. Only proteins with unambiguously assigned functions are included.

Based on these initial findings, we extended this comparative analysis to the two strains at 37 °C at the three growth times assayed (i.e., including 16 h and 24 h). In Table S4 and Fig. 3, we show the comparison between the strain expressing full-length ATX3Q26 and the control strain at the three times. In these comparisons, 115 distinct proteins showed differential expression levels between ATX3Q26 and control vector. In detail, at 16 h 44 proteins were differentially expressed (43 were up-regulated in ATX3Q26, among which 13 were exclusive of this condition, whereas only one protein was up-regulated in the control strain); at 24 h, there were 51 differential proteins (38 were up-regulated in ATX2Q26, among which 14 were exclusive in this condition; 13 were up-regulated in the control strain, among which 6 were exclusive in this condition). Finally, at 48 h, 69 proteins showed a different expression pattern (49 were up-regulated in ATX3Q26, among which 10 were exclusive in this condition; 20 were up-regulated in the control strain, among which 3 were exclusive in this condition).

This comparison was performed between full-length ATX3Q26 and the control strain (transformed with the empty vector), as our attempts to express catalytically inactive, full-length variant (ATX3Q26-C14A) were unsuccessful. To disprove the possibility that the expression pattern detected results from an unspecific effect of ATX3 overexpression, we also performed a comparison between the strains expressing JD and JD-C14A at 37 °C and 48 h of incubation (Table S4 and Fig. 3). Specifically, out of 47 proteins reported in Table S4, 40 were upregulated (among which 6 were exclusive) in the JD strain; 7 up-regulated in the JD-C14A strain.

**Figure 4:**
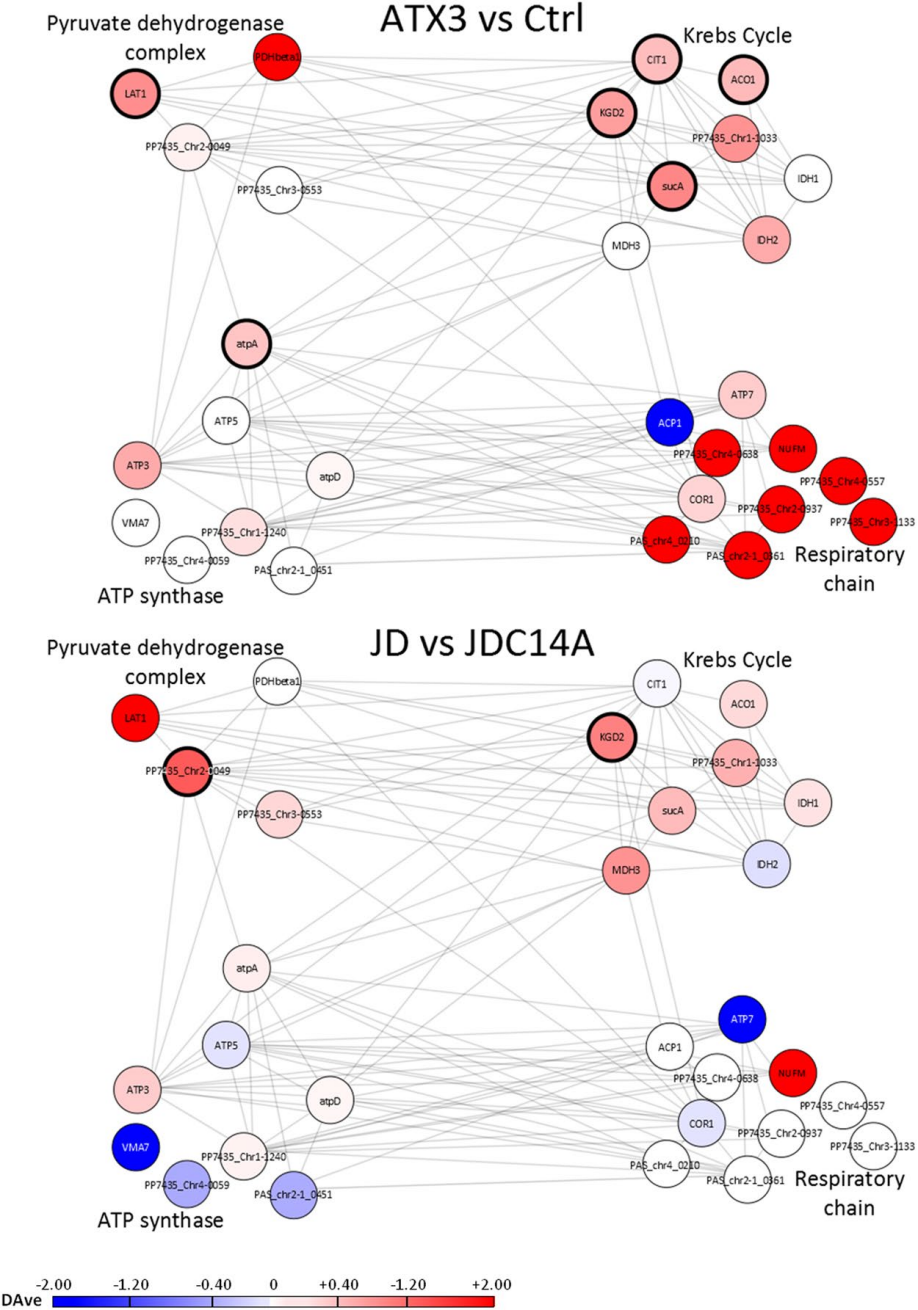
Differential changes in *P*. *pastoris* proteins related to mitochondrial energy metabolism during the growth at 37 °C. The differential expression was determined at 48 h growth time using the MAProMa comparison between the ATX3-trasformed- versus the control strain, and JD-transformed- versus JDC14A-transformed strain. Each protein was identified by its gene name (see Supplementary Table S5 for the complete reference) and represented as a node. Protein-protein interactions are indicated as grey edges. The color code of distinct nodes represents the DAve value and the relevant chromatic scale (reported in figure) ranges from −2.00 to 0 (dark blue to white) and from 0 to + 2.00 (white to dark red). The bold borders indicate DAve and DCI values that pass the filters: DAve ≥ l0.4 l and a DCI ≥ l400l. The proteins are grouped into four mitochondrial sub-pathways.

In the above results, we set filters at a DAve ≥ l0.4 l and a DCI ≥ l400l to maximize the confidence of the identification of the differentially expressed proteins, so the data in Fig. 3 and Table S4 only show these proteins. Under these conditions, only 11 out of the 53 differentially proteins identified (reported in Fig. 3) in the comparison ATX3 vs Ctrl at 48 h were also detected in the comparison JD vs JDC14A at the same incubation time (21%). The DAve and DCI filters we adopted allow identifying only proteins displaying an outstanding differential expression. However, when we also included comparisons whose Dave and DCI values do not pass the filter (marked with a hyphen in Table S4), we selected a wider protein ensemble of up- or downregulated proteins, which provided a more comprehensive view of the pathways underlying the protective effects fulfilled by ATX3 expression. In such case, 70 out of 112 distinct proteins with unambiguously assigned functions (63%) displayed the same pattern (data not shown). The significance of these findings was confirmed by means of the STRING database. This tool provides knowledge of biologically relevant interactions between the expressed proteins by consolidating known and predicted protein-protein association data, both physical and functional^35^. This enabled us to first select proteins belonging to the same compartment, referred to as “mitochondrial energy metabolism”, whereby “compartment” identifies the same subcellular localization, according to the aforementioned database. Then, we clustered functionally the proteins identified into four groupings, as shown in Fig. 4 and Table S5, each protein being represented in the figure as a node and grey edges identifying protein-protein interactions, both known and predicted.

As apparent in the figure, proteins belonging to the functional groupings “pyruvate dehydrogenase complex” and “Krebs cycle” show a similar trend in the two comparisons (i.e., ATX3 vs Ctrl and JD vs JDC14A). In contrast, in the case of “ATP synthase” this was proven to be only partially true, and in the grouping “respiratory chain” a significant divergence was detected, “ATX3 vs Ctrl” displaying a substantial upregulation, not observed in “JD vs JDC14A”. In conclusion, although this analysis highlights some discrepancy between the two comparisons, the changes observed in the latter are reasonably in line with the physiological responses induced by also JD.

To provide an independent validation of the above results, we quantified by Western blotting two proteins identified in the proteomic analysis. Our choice was restrained by the commercial availability of yeast antibodies, as well as by the need of selecting proteins displaying a fairly good expression level. We eventually opted for proteins appearing in comparisons displaying a DAve value of 2.00, which implies clear-cut differences in expression levels between the two compared conditions, i.e., phosphoglycerate kinase and glyoxalase I (Fig. S4). The expression levels quantified by Western blotting fit well with the proteomic data, as in the transformed strain the proteins assayed displayed expression levels about 8-fold greater than those detected in the control.

**Figure 5:**
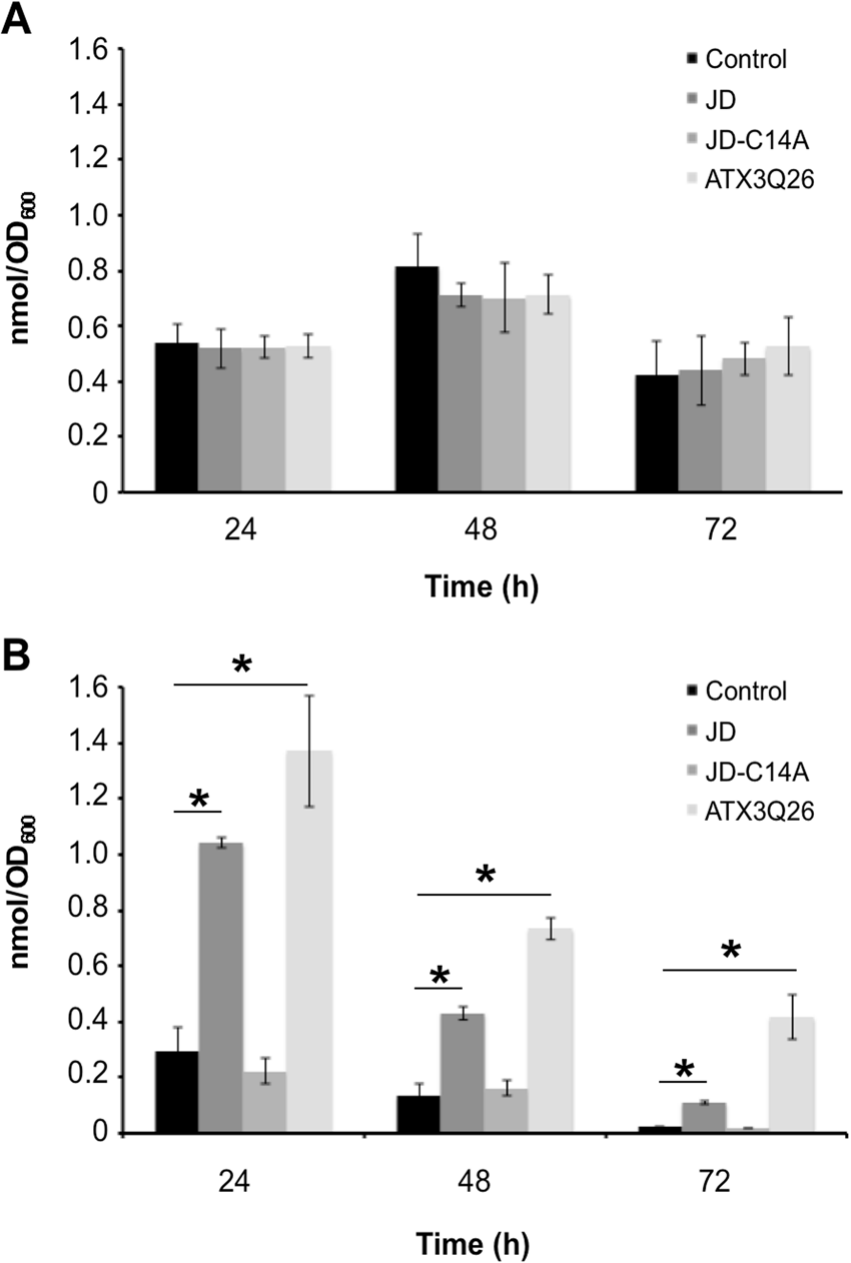
Determination of ATP levels at different growth times at 30 °C or 37 °C in strains expressing ATX3 variants. Cultures were grown at 30 °C (**A**) or 37 °C (**B**). At the indicated times samples were withdrawn and ATP assayed using the ENLITEN® ATP Assay System Bioluminescence Detection Kit. Data are expressed as nanomol of cells. Each experiments was performed in technical triplicate. Error bars represent standard deviations and are derived from at least three independent replicates. *p value < 0.01.

As outlined above, a prominent effect highlighted by the analysis performed on the strains growing at 37 °C is the ATX3-dependent upregulation of enzymes involved in mitochondrial energy metabolism (pyruvate dehydrogenase complex, Krebs cycle, respiratory chain, ATP synthase). We assume this might represent a compensatory effect to offset heat shock-induced energy depletion. The latter is a well-known phenomenon in eukaryotes, including fungi^36–38^, whose mechanisms are however only partially understood. This result prompted us to determine the ATP content in the strains under investigation during growth at both 30 °C and 37 °C (Fig. 5). At 30 °C, the nucleotide displayed marginal changes over time in all strains assayed, with no significant difference among them. However, in keeping with our assumption, at 37 °C it underwent a progressive decline in the control strain, its level at 24 h of growth being about one half of that detected at the same time at 30 °C, and becoming almost undetectable at 72 h. In contrast, in the strain transformed with full-length ATX3, ATP levels were either much higher than (24 h), or approximately equal to (48 and 72 h) those detected at 30 °C, although they also significantly declined over time. In the yeast transformed with the JD, ATP levels at 37 °C were restored to a lesser extent, consistent with the growth patterns presented in Figs 1 and 2, whereas the JD-C14A mutant was completely ineffective, which further confirms an essential role for ubiquitin hydrolase activity in sustaining growth at high temperature. Although the sole ATP depletion could fully account for the reduced growth at 37 °C, other effects triggered by the heat shock should quite likely be involved.

In any case, the reconstitution of ATP levels at 37 °C, which was apparent in the ATX3-transformed, as compared with the control strain, might account for much, if not all, of the changes in the proteome pattern at high temperature. In particular, the elevated energy charge was paralleled by an anabolic switch, as supported by a persistent upregulation of enzymes involved in both protein biosynthesis and biosynthetic pathways. Such upregulation represents the expected reprogramming of the expression pattern at high energy charge, a scenario that supports anabolism and active cell proliferation.

Independent and substantial evidence supporting the key role of ATX3-stimulated oxidative metabolism in sustaining growth at 37 °C was provided by comparing the growth rate under aerobic conditions with that under limited anaerobic conditions (Table 1). We observed that at 37 °C oxygen deprivation severely compromised the growth of the ATX3-transformed strain, which under these conditions was comparable to that of the control strain. In contrast, as expected, the latter underwent a scanty, fully oxygen-insensitive growth.

**Table 1:** Growth of ATX3-transformed and control (transformed with empty vector) *P*. *pastoris* strains in YPD medium at 30 °C and 37 °C under aerobic and limited anaerobic conditions.

Growth time and temperature	ATX3 transformed		Control	
**30 °C**	**Aerobic**	**Anaerobic**	**Aerobic**	**Anaerobic**
24 h	17.2 ± 1.4	0.78 ± 0.06	15.5 ± 0.8	0.66 ± 0.03
48 h	27.5 ± 0.6	0.84 ± 0.07	23.5 ± 0.3	0.86 ± 0.04
**37 °C**	**Aerobic**	**Anaerobic**	**Aerobic**	**Anaerobic**
24 h	3.35 ± 0.42	0.38 ± 0.01	0.24 ± 0.02	0.30 ± 0.04
48 h	8.81 ± 1.11	0.35 ± 0.06	0.23 ± 0.02	0.22 ± 0.02

Values are the mean of three determinations ± standard deviations. For other details, see Materials and Methods.

An additional feature highlighted by the proteome pattern is the upregulation of also several stress-induced proteins, which was noticeable especially at the earliest time, whereas at 24 h and 48 h their number decreased, along with the appearance of a number of downregulated hits. This points to a more prompt heat-stress response in the ATX3-transformed, compared with the control strain.

Overall, these results highlight a unique expression pattern, whereby both anabolic and catabolic/stress-related proteins are simultaneously upregulated.

Finally, at the latest time (48 h) we also observed substantial upregulation of enzymes involved in ethanol assimilation, with four hits detected. This is an obvious consequence of the faster glucose consumption by the ATX3-transformed strain and the resulting ethanol formation (Table S6). In fact, already at 24 h the alcohol was present in substantial amounts in the medium, and concomitantly at 48 h the sugar dropped to less than 15% of the initial value. In contrast, in the case of the control strain, no ethanol was detected in the medium throughout the growth, nor was glucose appreciably consumed, clearly due to the much slower growth rate.

## Discussion

The present investigation was initially undertaken to provide insight into the mechanisms of pathogenicity of expanded ATX3 variants, using *P*. *pastoris* as a model organism. However, our attention was soon drawn by the capability of wild-type ATX3 expressed in the microorganism to sustain its growth at a temperature well above the optimum values (i.e., 37 °C versus 30 °C). This prompted us to undertake the present research, aimed at clarifying the cellular mechanisms underlying the acquired heat resistance. In doing so, we mainly took advantage of the MudPIT proteomic technology. We produced three yeast strains that constitutively expressed wild-type full-length protein (ATX3Q26), the Josephin domain in isolation (JD), the catalytically inactive JD (JD-C14A), respectively. We also tried to transform the yeast with a catalytically inactive, full-length variant (ATX3Q26-C14A). However, our repeated attempts to express it were unsuccessful, although this mutant is capable of achieving a correct fold, as shown by previous circular dichroism data^30^. At the moment, the reasons for this outcome are unclear.

Based on our results, the capability to grow at high temperature conferred on *P*. *pastoris* by wild-type ATX3 expression was accompanied by substantial changes in the proteome pattern, with mostly upregulated, but also downregulated hits. Such an extensive reprogramming of the expression profile may appear at first glance surprising, as no ATX3 or related proteins are found in this yeast. It should be pointed out, however, that ATX3 is widespread throughout eukaryotes, including some other yeast species^39–41^. This suggests that the pathways under control of ATX3 might have been conserved in this as in other yeasts even in the absence of the protein, which may account for the effects detected in the present investigation. On the other hand, ATX3 is apparently dispensable, as substantiated by the viability of knock-out mice^42^ and *Caenorhabditis elegans* animal models^43^, which might justify its absence in *P*. *pastoris*.

One major, as yet unreported effect resulting from ATX3 expression was the upregulation of several enzymes involved in mitochondrial energy metabolism (i.e., pyruvate dehydrogenase complex, Krebs cycle, respiratory chain, ATP synthase), which persisted and even increased until the latest time monitored (48 h). This can quite plausibly justify the substantial increase in ATP levels, which were much higher than those observed at physiological temperature in the control strain. In contrast, the latter was subject to a dramatic depletion of the nucleotide when growing at 37 °C (Fig. 5). As a further support to the role of mitochondrial energy metabolism in sustaining thermosresistance, we observed that at 37 °C under conditions of limited anaerobiosis the growth of the ATX3-transformed strain was substantially impaired, thus becoming indistinguishable from that of the untransformed one (Table 1).

The cellular events triggered by heat shock in eukaryotes are complex and far from being fully elucidated, although it is reported that defects of the cytoskeleton represent one of the major damages^44^. In any case, drops in ATP levels following exposure to high temperature have been observed in fungi^38^, *Tetrahymena*^36^ and human cancer cells^37^. Whatever may be the mechanism underlying ATP depletion at 37 °C, its reconstitution effected by ATX3 expression can fully justify the resulting, persisting anabolic switch, which involved proteins taking part in both protein biosynthesis and in diverse biosynthetic pathways. In turn, this is the prerequisite for sustained growth at high temperature. Paradoxically, this scenario implies the simultaneous upregulation of both anabolic and catabolic pathways, which may be therefore regarded as the hallmark of ATX3 expression in this eukaryotic host.

A further, major reprogramming of the expression pattern was the upregulation of a set of stress-related proteins, mostly chaperones and enzymes protecting against oxidative stress/damage. In particular, several differentially upregulated proteins in the ATX3-transformed strain were spotted at the earliest time, i.e. 16 h, with eleven hits detected, but later their number declined, and concurrently downregulated hits were also recorded, representative of a similar but delayed response in the control strain.

In principle, the observed upregulation of stress proteins might result, at least in part, from an unspecific stressful effect induced by ATX3 protein overexpression. However, no way could this result in the adaptation mechanisms detected here. This is because overexpression of the catalytically inactive JD (JD-C14A) was completely devoid of any effect in terms of growth restoration and ATP levels reconstitution, whereas the functional counterpart exerted effects similar to, albeit less pronounced than, those of full-length ATX3. Additionally, the differential expression patterns observed in the two comparisons (ATX3 vs Ctrl and JD vs JD-C14A) displayed similar trends, although some proteins upregulated in the former were not in the latter. Furthermore, ATX3’s specific involvement in the response to stressful conditions is a well-known phenomenon. For instance, it modulates chaperone molecules at the transcriptional level, as supported by reduction in both basal and stress-induced levels of HSP70, as well as of its regulator Hsf1, in ATX3-deficient fibroblasts^45^. It also activates FOXO4-dependent transcription of the manganese superoxide dismutase-2 gene through direct interaction with the transcription factor^46^. Interestingly, the modulatory effects on the transcriptional activity apparently require ubiquitin hydrolase activity in the former but not in the latter case.

In any case, the ATX3-triggered energy metabolism activation is an as yet unknown phenomenon, which also might be regarded as an adaptation mechanism developed to cope with ATP depletion resulting from stressful conditions, as shown in the present investigation.

A key player in the adaptation to stressful conditions in higher eukaryotes is the AMP-dependent protein kinase (AMPK), whose ortholog in yeast is SNF1^47^. This is required for adaptation to both nutrient and environmental stresses, including heat shock. SNF1 is activated by three upstream kinases and allosterically by AMP. It acts as a transcriptional activator of several genes, and also inactivates enzymes involved in fatty acid biosynthesis and glycogen storage via phosphorylation. Furthermore, like AMPK in higher eukaryotes, SNF1 is a positive regulator of autophagy. Thus, it is expected that its activation will result in inhibition of anabolic processes and activation of catabolic ones, at both the transcriptional and the post-translational levels. As the high ATP levels at 37 °C in the ATX3-expressing strain must be paralleled by low AMP, we suggest that this condition might be responsible for the elevated expression of anabolic enzymes compared with those of the control strain, wherein ATP depletion is observed and high AMP is expected.

On the whole, the physiological condition detected at 37 °C strongly points to a low overall protein degradation rate in the ATX3-expressing strain and a high one in the control strain, as suggested by the respective ATP levels, which imply autophagy inhibition in the former and activation in the latter, also in keeping with the respective growth rates. Noteworthy, in this respect, is that the proteome analysis did not detect any change in the expression level of proteasome subunits, either 20S or 19S.

Besides the above remarks, it should be pointed out that he overall emerging picture of time-dependent changes in protein differential expression is unquestionably complex, so the interpretation of some data may not be straightforward, which is not surprising given the complexity of cellular/metabolic phenomena. However, plausible interpretations may be found for many of the changes detected. In particular, some trends observed in the comparison “ATX3 vs Ctrl” may be justified as follows: (i) as already mentioned, the downregulation or the loss in upregulation of some stress proteins at the latest growth times may be due to a slower adaptation of the control strain to stressful conditions compared with ATX3 strain; (ii) a similar trend observed for some glycolytic enzymes is an obvious consequence of glucose time course, as in the case of the ATX3-transformed strain the sugar was almost completely exhausted at 48 h and concurrently large ethanol concentrations accumulated in the medium. This should result in a metabolic switch from glycolytic to gluconeogenetic, which instead cannot take place in the control strain, in whose medium almost no glucose consumption and ethanol production took place; (iii) this metabolic condition also justifies upregulation of gluconeogenetic and ethanol-assimilating enzymes detected at 48 h.

Based on our results, the effects we have observed at 37 °C in the ATX3-transformed strain must be largely or exclusively mediated by the protein’s ubiquitin hydrolase activity, as clearly substantiated by the total inability of the mutated JD variant (JD-C14A), unlike the normal one, to sustain growth and reconstitute normal ATP levels. Conversely, also noteworthy is that JD could only partially reproduce the effects of full-length ATX3, both in terms of growth rate and ATP reconstitution at high temperature, as well as of repertoire of upregulated proteins, wherein those belonging to the grouping “respiratory chain” were absent. This highlights the role of the C-terminal, disordered domain in selecting substrates and fulfilling the multiplicity of effects underlying heat stress adaptation. Depending on the splice variant, this domain carries two or three UIMs that play important roles in ubiquitylated substrate recognition^14^. This further substantiates the idea that the effects detected are mediated by deubiquitylation of a defined repertoire of target proteins.

The mechanisms underlying the observed reprogramming of the expression pattern might be accounted for by both ATX3’s role in destining proteins for degradation via either the ERAD or the aggresome pathways^16^, and, more likely, by the aforementioned regulation phenomena at the transcriptional level^45,46^, which also would account for the wide range of proteins affected.

In conclusion, the present investigation demonstrates new roles for ATX3, further highlighting its involvement in the adaptation to stressful conditions via transcriptional regulation.

Our findings also broaden the scope of biotechnological applications for which *P*. *pastoris* may be suitable. In particular, fermentation systems devised for bioethanol production demand for thermotolerant yeasts strains, which in fact make it possible to improve yields and reduce costs^48,49^. Additionally, further investigations on our *Pichia* strain also offer the opportunity to better understand the mechanisms of yeast adaptation to high temperatures.

## Electronic supplementary material

Supplementary
