# Publisher Correction: The polyglutamine protein ataxin-3 enables normal growth under heat shock conditions in the methylotrophic yeast *Pichia pastoris*

**DOI:** 10.1038/s41598-018-22523-2

**Published:** 2018-03-06

**Authors:** Marcella Bonanomi, Valentina Roffia, Antonella De Palma, Alessio Lombardi, Francesco Antonio Aprile, Cristina Visentin, Paolo Tortora, Pierluigi Mauri, Maria Elena Regonesi

**Affiliations:** 1 Department of Biotechnology and Biosciences, University of Milano-Bicocca, 20126 Milan, Italy; 2 SYSBIO.IT, Centre of Systems Biology, 20126 Milano, Italy; 3 Institute for Biomedical Technologies, National Research Council, 20090 Milan, Italy; 4 Department of Chemistry, University of Cambridge, Cambridge, CB2 1EW United Kingdom; 5 Milan Center of Neuroscience (NeuroMI), 20126 Milano, Italy

Correction to: *Scientific Reports* 10.1038/s41598-017-13814-1, published online 17 October 2017.

The version of this Article previously published incorrectly indicated that Marcella Bonanomi and Valentina Roffia were the corresponding authors, rather than equally contributing authors. In addition, Paolo Tortora and Pierluigi Mauri were not listed as corresponding authors. Correspondence and request for materials should be addressed to paolo.tortora@unimib.it and pierluigi.mauri@itb.cnr.it.

The original version of this Article also contained an error in the legend of Figure 5.

“Determination of ATP levels at different growth times at 30 °C or 37 °C in strains expressing ATX3 variants. Cultures were grown at 30 °C or 37 °C. At the indicated times samples were withdrawn and ATP assayed using the ENLITEN® ATP Assay System Bioluminescence Detection Kit. Data are expressed as nanomol ${{\rm{ATP}}/{\rm{OD}}}_{600}$ of cells. Each experiments was performed in technical triplicate. Error bars represent standard deviations and are derived from at least three independent replicates. *p value < 0.01.”

now reads:

“Determination of ATP levels at different growth times at 30 °C or 37 °C in strains expressing ATX3 variants. Cultures were grown at 30 °C (A) or 37 °C (B). At the indicated times samples were withdrawn and ATP assayed using the ENLITEN® ATP Assay System Bioluminescence Detection Kit. Data are expressed as nanomol ${{\rm{ATP}}/{\rm{OD}}}_{600}$ of cells. Each experiments was performed in technical triplicate. Error bars represent standard deviations and are derived from at least three independent replicates. *p value < 0.01.”

These errors have now been corrected in the PDF and HTML versions of the Article.

