# Supplementary material for: The polyglutamine protein ataxin-3 enables normal growth under heat shock conditions in the methylotrophic yeast Pichia pastoris
Source: Sci Rep. 2017 Oct 17;7:13417. doi: 10.1038/s41598-017-13814-1 (PMC5645362; doi:10.1038/s41598-017-13814-1)
Supplement: Supplementary file 1 — Supplementary [file 41598_2017_13814_MOESM1_ESM.pdf]

The polyglutamine protein ataxin-3 enables normal growth under heat shock conditions in the methylotrophic yeast *Pichia pastoris*

Marcella Bonanomi, Valentina Roffia, Antonella De Palma, Alessio Lombardi,

Francesco Antonio Aprile, Cristina Visentin, Paolo Tortora, Pierluigi Mauri,

Maria Elena Regonesi

Supplementary figures legends

Fig. S1. **Western blot analysis of ATX3 expression levels at different growth times**. Whole protein extracts of *P. pastoris* strains expressing the ATX3 variants were obtained collecting cells at different time of growth (16-24-48 h) at 30°C or 37°C. Samples were subjected to SDS-PAGE (16%) and Western blotted using anti-ATX3 antibody. PSTAIR motif of cyclin-dependent kinases was used as a loading control.

Fig. S2. Example of technical repeatability of MudPIT analysis. To verify the repeatability of the proteomic approach, the Spectral Count (SpC) values of the first (X axis) and the second run (Y axis) were plotted. Linear correlation (R^2 value) and slope (y) are close to theoretical value of 1.

Fig. S3. Representative 2D virtual map plotted by MAProMa software. The identified proteins from the ATX3 strain (48 hours – 37°C) were plotted with MAProMa software according to their theoretical pI and MW. For each protein, a color/shape code is used according to the confidence of identification. Proteins with score ≤15 are reported as yellow triangles; proteins with score ≥35 are reported as red circles and proteins that with a score in the range 15-35 are plotted as blue squares.

Fig. S4. Validation of the mass spectrometry analysis by Western blotting. Whole protein extracts of *P. pastoris* strains were subjected to SDS-PAGE (14%), and Western blotted using either anti-phosphoglycerate kinase (PGK) or anti-glyoxalase I antibodies. PSTAIR motif of cyclin-dependent kinases was used as a loading control. Other details are reported in Materials and Methods. PGK was assayed in the ATX3Q26 strain versus control at 48 h of growth at 37°C. Glyoxalase I was assayed in the ATX3-JD strain versus the respective control (ATX3-JDC14A) at 48 h of growth at 37°C. In both cases, DAve values were 2.00. For each comparison, the respective expression ratios (transformed strain versus control) and standard deviations are reported. Data are the mean of three biological replicates, each of them being obtained from the mean of three technical replicates.

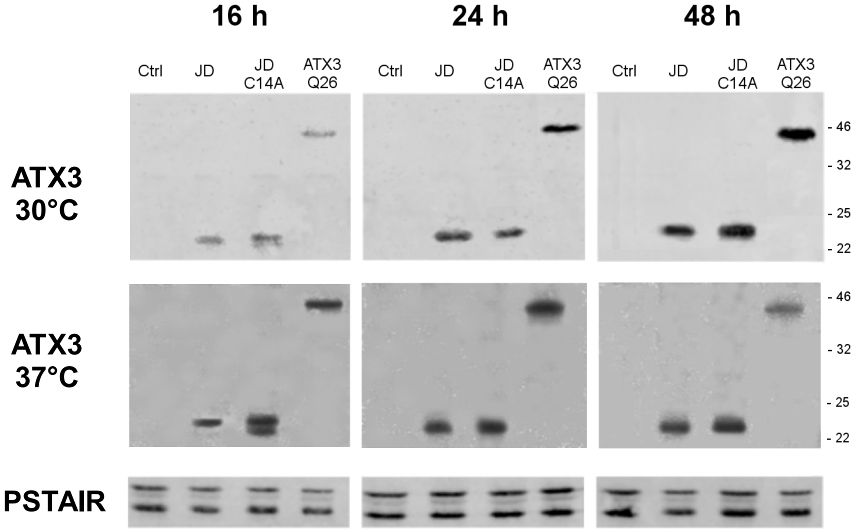
16 h
24 h
48 h
Ctrl
JD
JD C14A
ATX3 Q26
ATX3 30°C
ATX3 37°C
PSTAIR
- 46
- 32
- 25
- 22

Figure S1

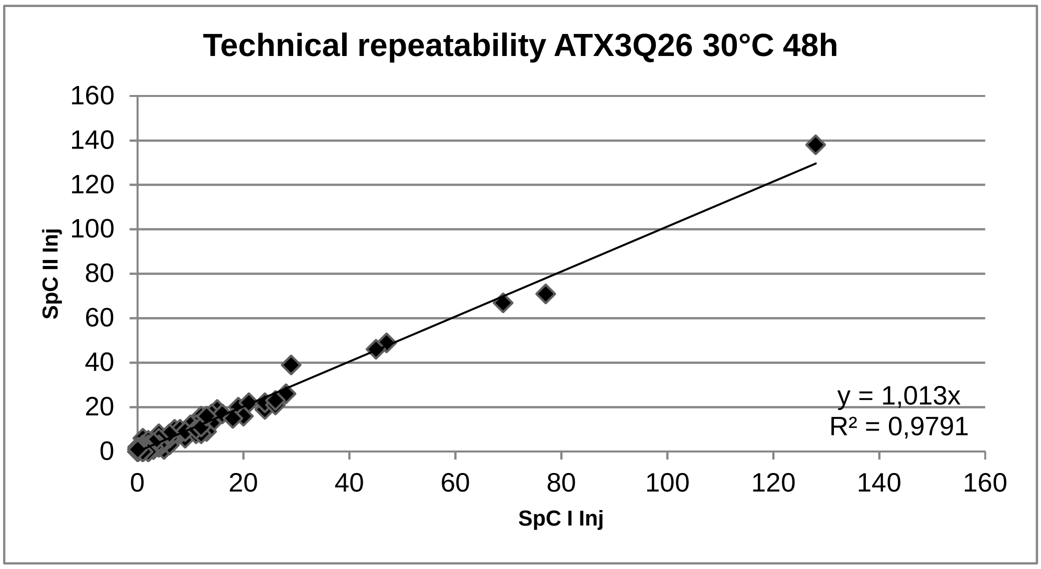

Technical repeatability ATX3Q26 30°C 48h
160
140
120
100
80
60
40
20
0
0
20
40
60
80
100
120
140
160
SpC II Inj
SpC I Inj
y = 1,013x
R² = 0,9791

Figure S2

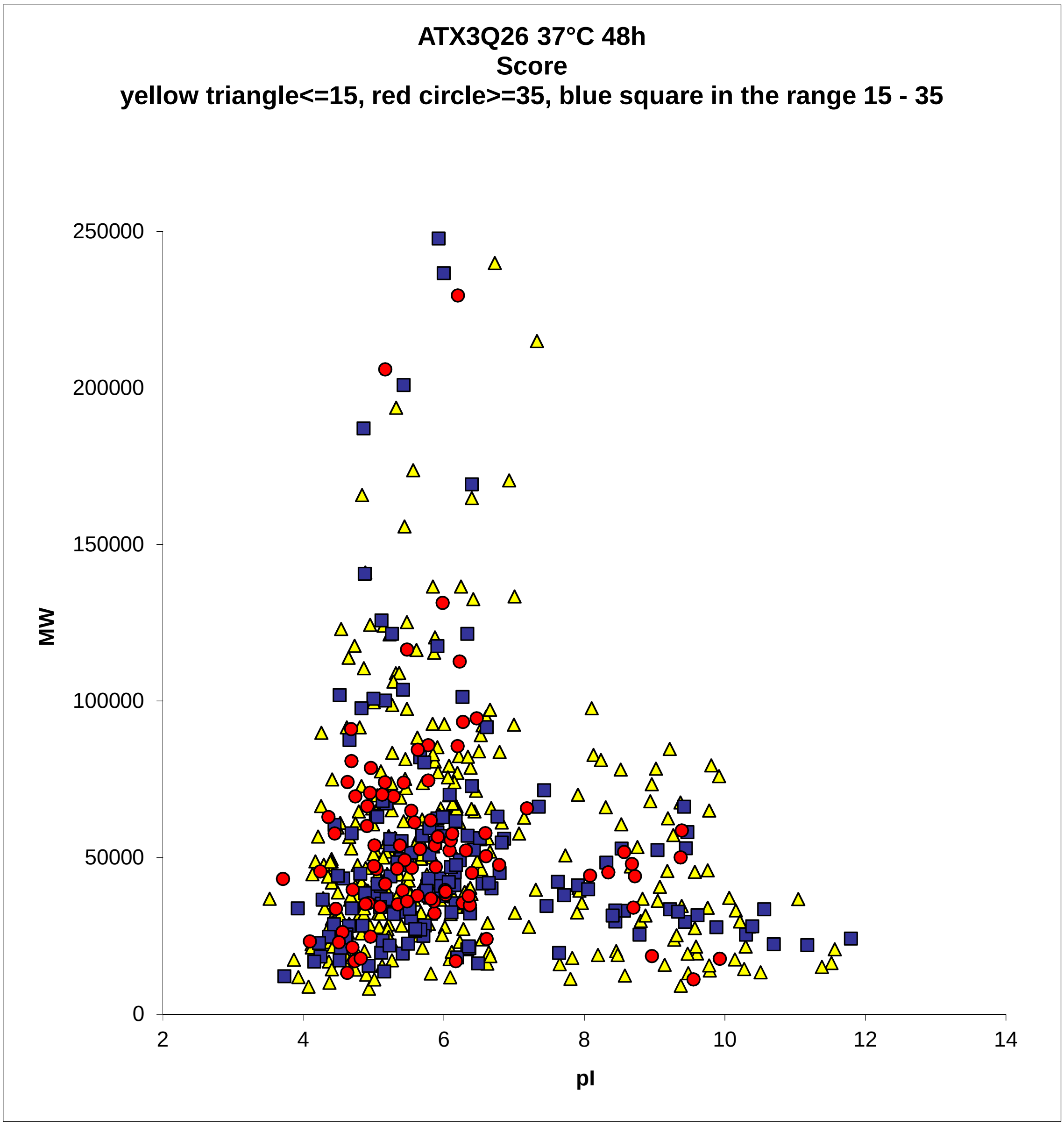
ATX3Q26 37°C 48h
Score
yellow triangle<=15, red circle>=35, blue square in the range 15 - 35
250000
200000
150000
100000
50000
0
MW
2
4
6
8
10
12
14
pI

Figure S3

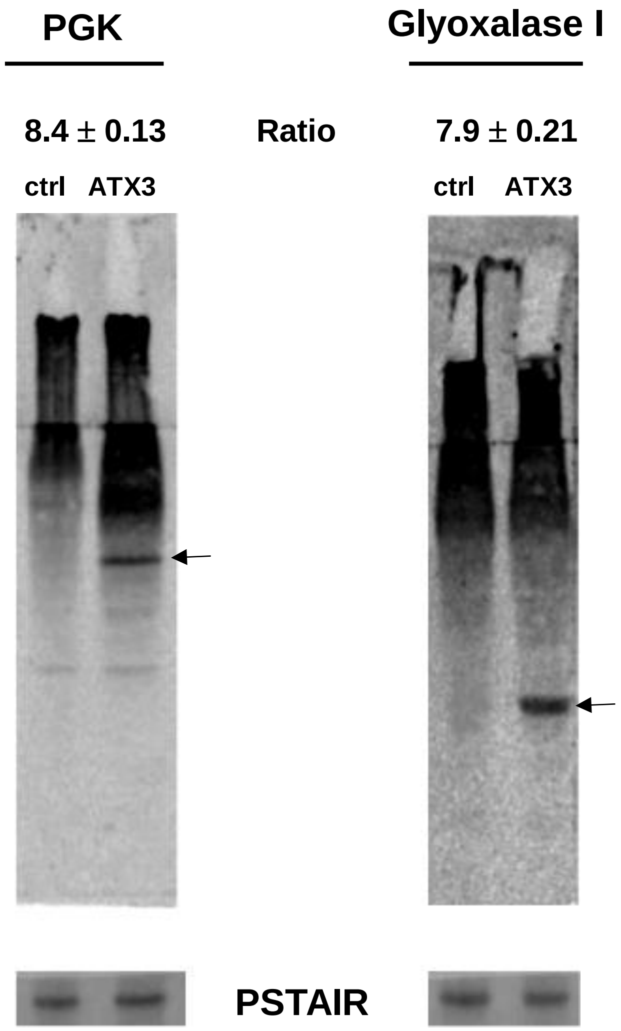
PGK
Glyoxalase I
8.4 ± 0.13
Ratio
7.9 ± 0.21
ctrl
ATX3
ctrl
ATX3
PSTAIR

Figure S4

Table S1. Complete list of the individual proteins detected in ATX3-transformed and control (transformed with empty vector) *P. pastoris* strains grown at either 30°C or 37°C for different times (48 h at 30°C; 16 h, 24 h, 48 h at 37°C). For each protein are reported: GI Accession number, Uniprot entry, Gene name, Reference, pI and MW, Frequency (Freq), SpC and Score. Frequency indicates how many times a given protein has been identified under each condition examined.The asterisk next to SpC and Score indicates that the mean values of two injections were given for each protein of the same condition.

Accession	Entry Uniprot	Gene name	Reference	pI	MW	30°C						37°C																	
						48h						16h						24h						48h					
						ATX3Q26			Ctrl			ATX3Q26			Ctrl			ATX3Q26			Ctrl			ATX3Q26			Ctrl		
						Freq	SpC*	Score*	Freq	SpC*	Score*	Freq	SpC*	Score*	Freq	SpC*	Score*	Freq	SpC*	Score*	Freq	SpC*	Score*	Freq	SpC*	Score*	Freq	SpC*	Score*
3203	P45353	HIS4	histidinol dehydrogenase	5,00	92163	2	2	15,18	2	1	10,17	2	1	10,20	0	0	0,00	1	1	10,13	0	0	0,00	0	0	0,00	0	0	0,00
1871627	P78992	PYC1	pyruvate carboxylase	5,98	131400	0	0	0,00	1	1	10,11	2	4	15,18	2	4	20,16	2	4	10,20	1	1	10,14	2	9	55,27	2	2	15,21
2624379	O13505	IPP1	inorganic pyrophosphatase (pyrophosphate phospho-hydrolase)	5,28	32030	0	0	0,00	0	0	0,00	1	1	10,14	0	0	0,00	1	1	10,13	2	6	30,19	2	2	18,16	0	0	0,00
12381850	Q9C1Z6	frs1	phenylalanyl-tRNA synthetase subunit	4,82	42192	0	0	0,00	0	0	0,00	2	2	20,18	0	0	0,00	2	2	20,15	0	0	0,00	1	1	10,17	0	0	0,00
13235614	Q9C1Z8	pdi	protein disulphide isomerase	4,44	57735	2	2	15,13	1	1	10,14	2	3	30,17	2	2	15,16	2	3	30,16	2	5	30,18	2	12	85,22	2	13	80,22
13397903	Q9C124	ICL1	isocitrate lyase	6,16	61744	2	4	10,22	2	5	15,21	0	0	0,00	0	0	0,00	0	0	0,00	0	0	0,00	2	2	15,19	1	1	10,16
13397905	Q9C123	met17	O-acetylhomoserine sulfhydrylase	5,80	48272	0	0	0,00	1	1	10,14	0	0	0,00	0	0	0,00	0	0	0,00	0	0	0,00	0	0	0,00	0	0	0,00
15825003	Q96V35	CBS	F367364_1 cystathionine beta-synthase	6,82	54887	1	1	10,19	0	0	0,00	1	1	10,13	0	0	0,00	0	0	0,00	0	0	0,00	2	2	20,20	2	1	10,15
19424735	Q8TGA0	PFK2	F395078_1 6-phosphofructo-1-kinase beta-subunit	6,60	103650	1	1	10,13	0	0	0,00	0	0	0,00	0	0	0,00	0	0	0,00	0	0	0,00	0	0	0,00	0	0	0,00
47132400	Q6GYJ7		methionine synthase	5,78	85966	2	27	133,25	2	23	100,25	2	4	30,16	0	0	0,00	2	6	39,20	1	4	40,17	2	12	59,20	2	17	50,22
57117457	Q5I6F4	ARO1	AROM pentafunctional enzyme, partial	6,39	169300	2	1	10,14	2	1	10,14	2	2	19,16	2	3	15,13	1	1	10,14	2	2	15,15	2	2	20,16	2	1	10,14
90110409	Q1W5W1		heat shock protein 70	4,91	66435	2	7	60,28	2	7	55,19	2	4	44,23	2	3	20,19	2	11	60,27	2	7	45,20	2	8	50,18	2	2	23,15
116293731	A0FJH5		translation elongation factor 1-alpha	9,37	50098	2	5	20,18	2	4	25,18	2	55	120,20	2	7	40,17	2	18	55,18	2	13	65,20	2	65	120,26	2	44	82,19
156099446	A8DN83		translation elongation factor 1-alpha, partial	7,96	35565	0	0	0,00	0	0	0,00	1	1	10,15	2	2	10,17	1	1	10,15	2	1	10,15	2	2	10,16	0	0	0,00
158426345	A9Q6B0		IF4E-like protein, partial	5,49	22592	0	0	0,00	0	0	0,00	0	0	0,00	0	0	0,00	0	0	0,00	0	0	0,00	2	5	25,17	1	1	10,13
224994879	C0LQF6	TKL1	transketolase 1, partial	5,78	74646	2	11	75,25	2	9	55,24	2	5	35,24	0	0	0,00	2	16	70,25	2	6	35,15	2	21	120,26	2	16	55,24
238034185	C4R9D1	PAS_c034_0007	transcriptional regulatory protein	6,19	77173	0	0	0,00	0	0	0,00	0	0	0,00	0	0	0,00	0	0	0,00	0	0	0,00	1	1	10,11	0	0	0,00
238034194	C4R9E0	PP7435_Chr1-1573	Alcohol dehydrogenase	6,35	37816	0	0	0,00	0	0	0,00	0	0	0,00	0	0	0,00	2	2	10,16	0	0	0,00	2	11	64,19	1	1	10,14
238034205	C4R9F1	SSE1	Heat shock protein	4,96	78709	2	4	20,18	2	6	30,18	2	15	80,26	2	5	42,24	2	21	95,23	2	8	65,19	2	11	85,22	2	9	65,23
238034214	C4R9G0	PAS_c034_0038	26S proteasome non-ATPase regulatory particle subunit	4,52	31669	0	0	0,00	0	0	0,00	0	0	0,00	1	1	10,13	0	0	0,00	0	0	0,00	1	1	10,16	0	0	0,00
238034215	C4R9G1	PAS_c034_0039	Peptidyl-prolyl isomerase	6,19	85597	0	0	0,00	0	0	0,00	2	2	15,17	2	5	25,20	0	0	0,00	2	4	30,18	2	6	50,21	2	9	50,20
254564465	C4QUY9	PP7435_Chr1-0001	hypothetical protein	5,97	55916	0	0	0,00	0	0	0,00	0	0	0,00	0	0	0,00	0	0	0,00	1	1	10,11	0	0	0,00	0	0	0,00
254564477	C4QUZ5	PP7435_Chr1-0008	hypothetical protein	5,69	21277	0	0	0,00	0	0	0,00	0	0	0,00	0	0	0,00	0	0	0,00	0	0	0,00	2	1	10,15	2	3	20,15
254564517	C4QV15	PP7435_Chr1-0027	Nuclear SAM-dependent mono-and asymmetric arginine dimethylating methyltransferase	5,06	39019	0	0	0,00	0	0	0,00	2	4	20,20	2	2	15,15	2	2	15,18	2	2	10,18	2	2	10,20	1	1	10,17
254564519	C4QV16	PP7435_Chr1-0028	60S ribosomal protein L4	11,04	36865	1	1	10,14	2	1	10,15	2	3	20,22	0	0	0,00	2	4	20,25	0	0	0,00	2	5	10,28	1	1	10,19
254564539	C4QV26	iga	Putative protein of unknown function	4,40	61065	1	1	10,20	0	0	0,00	0	0	0,00	0	0	0,00	0	0	0,00	0	0	0,00	0	0	0,00	0	0	0,00
254564559	C4QV36	TPS2	Phosphatase subunit of the trehalose-6-phosphate synthase/phosphatase complex	6,60	95091	0	0	0,00	0	0	0,00	0	0	0,00	0	0	0,00	0	0	0,00	0	0	0,00	2	1	10,15	0	0	0,00
254564587	C4QV50	RPP0	60S acidic ribosomal protein P0	4,46	33684	1	1	10,13	0	0	0,00	2	12	54,18	2	4	25,19	2	4	19,16	2	6	25,16	2	11	49,21	2	7	30,18
254564589	C4QV51	RSN1	Membrane protein of unknown function	8,10	97792	0	0	0,00	0	0	0,00	0	0	0,00	0	0	0,00	0	0	0,00	0	0	0,00	1	1	10,13	0	0	0,00
254564591	C4QV52	PPA2	Mitochondrial inorganic pyrophosphatase	5,39	33237	0	0	0,00	0	0	0,00	0	0	0,00	0	0	0,00	0	0	0,00	0	0	0,00	2	1	10,15	0	0	0,00
254564599	C4QV56	PAS_chr1-3_0074	Cytoplasmic ATP-dependent RNA helicase of the DEAD-box family	5,70	53920	0	0	0,00	2	1	10,13	2	1	10,20	1	1	10,19	2	1	10,18	0	0	0,00	2	1	10,17	0	0	0,00
254564607	C4QV60	PDT1	hypothetical protein	6,55	41820	1	1	16,12	1	1	10,12	1	1	10,11	0	0	0,00	1	1	10,14	0	0	0,00	2	4	15,18	2	16	25,22
254564611	C4QV62	PP7435_Chr1-0076	Nuclear protein that inhibits replication of Brome mosaic virus in S. cerevisiae	4,40	49619	0	0	0,00	0	0	0,00	0	0	0,00	0	0	0,00	0	0	0,00	0	0	0,00	1	1	10,13	0	0	0,00
254564619	C4QV66	PAS_chr1-3_0083	Catalytic subunit of DNA polymerase (II) epsilon	6,22	254566	0	0	0,00	0	0	0,00	1	1	10,11	0	0	0,00	0	0	0,00	0	0	0,00	0	0	0,00	0	0	0,00
254564641	C4QV77	PAS_chr1-3_0092	Putative protein of unknown function	4,38	23065	0	0	0,00	0	0	0,00	0	0	0,00	0	0	0,00	0	0	0,00	0	0	0,00	1	1	10,16	0	0	0,00
254564647	C4QV80	KGD2	Dihydrolipoyl transsuccinylase, component of the mitochondrial alpha-ketoglutarate dehydrogenase	8,67	48038	0	0	0,00	0	0	0,00	2	1	10,16	2	2	15,17	2	1	10,15	1	1	10,16	2	5	45,20	2	3	20,19
254564663	C4QV88	TIF6	Constituent of 66S pre-ribosomal particles, has similarity to human translation initiation factor 6	4,38	26309	0	0	0,00	0	0	0,00	1	1	10,14	1	2	20,14	0	0	0,00	2	1	10,19	1	1	10,14	0	0	0,00
254564665	C4QV89	clpB	Heat shock protein that cooperates with Ydj1p (Hsp40) and Ssa1p (Hsp70)	5,16	100224	0	0	0,00	0	0	0,00	2	2	15,15	0	0	0,00	1	1	10,14	0	0	0,00	2	2	15,17	0	0	0,00
254564667	C4QV90	ACO1	Aconitase, required for the tricarboxylic acid (TCA) cycle and also independently required for mitoc	5,63	84478	2	17	80,22	2	15	75,22	2	15	45,20	1	4	40,18	2	11	35,20	2	4	30,19	2	33	85,24	2	10	49,22
254564669	C4QV91	PAS_chr1-3_0299	hypothetical protein	5,96	72961	0	0	0,00	0	0	0,00	0	0	0,00	0	0	0,00	0	0	0,00	0	0	0,00	0	0	0,00	1	1	10,11
254564695	C4QVA4	PP7435_Chr1-0120	hypothetical protein	9,37	9145	0	0	0,00	0	0	0,00	0	0	0,00	0	0	0,00	0	0	0,00	0	0	0,00	1	1	10,15	0	0	0,00
254564705	C4QVA9	UGP1	UDP-glucose pyrophosphorylase (UGPase), catalyses the reversible formation of UDP-Glc	6,51	56370	1	1	10,15	0	0	0,00	0	0	0,00	0	0	0,00	0	0	0,00	0	0	0,00	2	5	25,21	0	0	0,00
254564717	C4QVB5	CPGL	Probable di-and tri-peptidase	5,23	55999	2	5	30,17	2	1	10,14	2	1	10,17	0	0	0,00	2	2	10,19	0	0	0,00	2	3	25,16	2	4	20,20
254564783	C4QVE8	PAS_chr1-3_0160	Putative protein of unknown function	5,15	71888	1	1	10,19	1	1	10,18	0	0	0,00	0	0	0,00	0	0	0,00	0	0	0,00	1	1	10,16	0	0	0,00
254564799	C4QVF6	PAS_chr1-3_0167	Protein with a role in 5'-end processing of mitochondrial RNAs	9,02	78468	0	0	0,00	0	0	0,00	0	0	0,00	0	0	0,00	0	0	0,00	0	0	0,00	1	1	10,16	0	0	0,00
254564809	C4QVG1	PAS_chr1-3_0172	Subunit beta1 of the nascent polypeptide-associated complex (NAC) involved in protein targeting	4,66	17904	0	0	0,00	0	0	0,00	0	0	0,00	0	0	0,00	0	0	0,00	0	0	0,00	0	0	0,00	1	1	10,11
254564825	C4QVG9	PP7435_Chr1-0180	hypothetical protein	5,12	15578	0	0	0,00	0	0	0,00	1	1	10,12	0	0	0,00	0	0	0,00	0	0	0,00	2	1	10,20	0	0	0,00
254564827	C4QVH0	EFR3	Non-essential protein of unknown function	8,13	82834	0	0	0,00	0	0	0,00	0	0	0,00	0	0	0,00	0	0	0,00	0	0	0,00	1	1	10,12	0	0	0,00
254564829	C4QVH1	PAS_chr1-3_0181	Protein of unknown function	9,12	166953	0	0	0,00	0	0	0,00	0	0	0,00	0	0	0,00	1	1	10,14	0	0	0,00	0	0	0,00	0	0	0,00
254564849	C4QVI1	PAS_chr1-3_0191	GMP synthase, an enzyme that catalyzes the second step in the biosynthesis of GMP from IMP	5,21	56954	0	0	0,00	0	0	0,00	0	0	0,00	0	0	0,00	0	0	0,00	1	1	10,10	2	2	10,17	0	0	0,00
254564851	C4QVI2	RAM2	Alpha subunit of both the farnesyltransferase and type I geranylgeranyltransferase	4,77	37563	0	0	0,00	0	0	0,00	0	0	0,00	0	0	0,00	0	0	0,00	0	0	0,00	1	1	10,13	0	0	0,00
254564867	C4QVJ0	PP7435_Chr1-0202	hypothetical protein	9,04	36260	0	0	0,00	0	0	0,00	0	0	0,00	0	0	0,00	0	0	0,00	0	0	0,00	2	1	10,18	0	0	0,00
254564871	C4QVJ2	PP7435_Chr1-0204	Essential subunit of Sec61 complex (Sec61p, Sbh1p, and Sss1p)	9,04	52509	0	0	0,00	0	0	0,00	0	0	0,00	0	0	0,00	0	0	0,00	0	0	0,00	2	2	15,16	0	0	0,00
254564891	C4QVK2	DYS1	Deoxyhypusine synthase, catalyzes formation of deoxyhypusine	4,87	41461	1	1	10,11	0	0	0,00	0	0	0,00	1	1	10,13	0	0	0,00	0	0	0,00	0	0	0,00	1	1	10,13
254564915	C4QVL4	GAS1	Beta-1,3-glucanosyltransferase, required for cell wall assembly	3,86	57236	0	0	0,00	0	0	0,00	0	0	0,00	0	0	0,00	0	0	0,00	0	0	0,00	0	0	0,00	2	1	10,21
254564917	C4QVL5	PHR2	Beta-1,3-glucanosyltransferase, required for cell wall assembly	3,84	58251	0	0	0,00	0	0	0,00	0	0	0,00	0	0	0,00	0	0	0,00	0	0	0,00	0	0	0,00	2	12	70,24
254564923	C4QVL8	RTN1	ER membrane protein that interacts with exocyst subunit Sec6p and with Yip3p	7,89	32514	0	0	0,00	0	0	0,00	0	0	0,00	0	0	0,00	0	0	0,00	1	1	10,12	2	1	10,19	0	0	0,00
254564929	C4QVM1	PP7435_Chr1-0238	Mitogen-activated protein kinase involved in osmoregulation via three independent osmosensors	5,03	40306	0	0	0,00	0	0	0,00	0	0	0,00	0	0	0,00	0	0	0,00	0	0	0,00	2	1	10,20	0	0	0,00
254564981	C4QVP7	lsm5	hypothetical protein	3,80	8673	1	1	10,11	2	1	10,13	0	0	0,00	0	0	0,00	0	0	0,00	0	0	0,00	0	0	0,00	0	0	0,00
254564989	C4QVQ1	PP7435_Chr1-0266	hypothetical protein	4,27	36663	2	3	20,19	2	3	20,20	2	3	20,17	0	0	0,00	2	5	25,23	0	0	0,00	2	4	25,24	1	1	10,14
254565001	C4QVQ7	SPY	Putative protein of unknown function	4,13	44754	0	0	0,00	0	0	0,00	0	0	0,00	0	0	0,00	0	0	0,00	0	0	0,00	1	1	10,18	0	0	0,00
254565045	C4QVS9	PMA1	Plasma membrane H+-ATPase, pumps protons out of the cell	4,82	97797	2	14	70,29	2	10	65,27	2	2	10,23	0	0	0,00	1	1	10,24	1	1	10,19	2	4	30,27	2	29	105,28
254565049	C4QVT1	PAS_chr1-1_0459	Dolichol-phosphate mannosyltransferase	5,78	28893	0	0	0,00	0	0	0,00	0	0	0,00	0	0	0,00	0	0	0,00	0	0	0,00	2	1	10,14	1	1	10,12
254565055	C4QVT4	PP7435_Chr1-0296	U3 small nucleolar RNA-associated protein	7,39	104060	0	0	0,00	0	0	0,00	0	0	0,00	0	0	0,00	1	1	10,13	0	0	0,00	0	0	0,00	0	0	0,00
254565063	C4QVT8	FAS1	Beta subunit of fatty acid synthetase	6,20	229580	2	8	40,19	2	7	35,19	2	5	30,19	2	3	25,20	2	9	45,20	0	0	0,00	2	15	60,21	0	0	0,00
254565081	C4QVU7	PP7435_Chr1-0310	hypothetical protein	4,69	26986	1	1	10,11	0	0	0,00	0	0	0,00	0	0	0,00	0	0	0,00	0	0	0,00	0	0	0,00	0	0	0,00
254565083	C4QVU8	POB3	Subunit of the heterodimeric FACT complex (Spt16p-Pob3p)	4,66	59955	0	0	0,00	0	0	0,00	2	1	10,22	0	0	0,00	0	0	0,00	0	0	0,00	0	0	0,00	0	0	0,00
254565129	C4QVX1	SSZ1	Hsp70 protein that interacts with Zuo1p (a DnaJ homolog) to form a ribosome-associated complex	4,68	57863	0	0	0,00	0	0	0,00	0	0	0,00	0	0	0,00	0	0	0,00	0	0	0,00	2	2	20,14	0	0	0,00
254565137	C4QVX5	UBP7	Ubiquitin carboxyl-terminal hydrolase	7,80	108666	1	1	10,17	0	0	0,00	0	0	0,00	0	0	0,00	0	0	0,00	0	0	0,00	0	0	0,00	0	0	0,00
254565157	C4QVY5	LAT1	Dihydrolipoamide acetyltransferase component (E2) of pyruvate dehydrogenase complex	6,60	50528	0	0	0,00	0	0	0,00	1	1	10,13	0	0	0,00	1	2	20,13	2	1	10,16	2	4	35,20	2	2	15,17
254565163	C4QVY8	PP7435_Chr1-0352	Translation initiation factor eIF4G, subunit of the mRNA cap-binding protein complex (eIF4F)	5,22	121335	0	0	0,00	0	0	0,00	0	0	0,00	0	0	0,00	0	0	0,00	0	0	0,00	1	1	10,14	0	0	0,00
254565185	C4QVZ9	PP7435_Chr1-0363	Protein of unknown function that associates with ribosomes and has a putative RNA binding domain	6,59	20276	0	0	0,00	0	0	0,00	0	0	0,00	0	0	0,00	2	2	15,17	0	0	0,00	0	0	0,00	1	1	10,14
254565191	C4QW02	PP7435_Chr1-0368	Putative X-Pro aminopeptidase	5,20	53791	1	1	10,14	0	0	0,00	0	0	0,00	0	0	0,00	0	0	0,00	0	0	0,00	1	1	10,14	0	0	0,00
254565203	C4QW08	PAS_chr1-1_0071	hypothetical protein	5,30	44744	1	1	10,14	0	0	0,00	0	0	0,00	0	0	0,00	0	0	0,00	0	0	0,00	0	0	0,00	0	0	0,00
254565205	C4QW09	FBA1	Fructose 1,6-bisphosphate aldolase, required for glycolysis and gluconeogenesis	6,02	39689	2	46	30,21	2	24	45,23	2	5	15,20	1	1	10,15	2	2	15,19	2	2	15,18	2	10	55,26	2	9	43,20
254565207	C4QW10	PAS_chr1-1_0470	hypothetical protein	6,07	69961	2	2	10,15	2	1	10,16	2	2	10,19	0	0	0,00	1	1	10,21	2	1	10,13	1	1	10,20	2	1	10,19
254565225	C4QW19	NPL3	RNA-binding protein that carries poly(A)+ mRNA from the nucleus into the cytoplasm	5,50	37290	0	0	0,00	0	0	0,00	0	0	0,00	1	1	10,12	1	1	10,16	0	0	0,00	0	0	0,00	0	0	0,00
254565237	C4QW25	RRS1	Essential protein that binds ribosomal protein L11	9,62	22455	0	0	0,00	0	0	0,00	2	1	10,17	1	2	10,19	0	0	0,00	0	0	0,00	0	0	0,00	0	0	0,00
254565243	C4QW28	PP7435_Chr1-0393	Protein involved in programmed cell death	4,87	80918	0	0	0,00	0	0	0,00	0	0	0,00	0	0	0,00	1	1	10,13	0	0	0,00	0	0	0,00	0	0	0,00
254565261	C4QW37	RSM7	mitochondrial 37S ribosomal protein RSM7	10,34	28090	0	0	0,00	0	0	0,00	0	0	0,00	0	0	0,00	0	0	0,00	0	0	0,00	1	1	10,16	0	0	0,00
254565263	C4QW38	COR1	Core subunit of the ubiquinol-cytochrome c reductase complex (bc1 complex)	5,00	47391	2	2	10,16	2	2	15,14	2	4	35,23	1	2	20,20	2	5	50,19	2	2	10,15	2	17	85,20	2	8	60,21
254565273	C4QW43	PP7435_Chr1-0406	Putative protein proposed to be involved in the metabolism of purine and pyrimidine base analogues	4,96	24834	0	0	0,00	0	0	0,00	2	1	10,16	1	1	10,13	1	1	10,16	0	0	0,00	2	8	35,23	2	4	20,18
254565279	C4QW46	GDH1	NADP(+)-dependent glutamate dehydrogenase	5,44	49258	2	48	100,24	2	78	135,25	2	9	45,21	2	3	20,15	2	22	70,24	2	11	55,19	2	32	90,26	2	28	95,25
254565299	C4QW56	PP7435_Chr1-0419	hypothetical protein	7,69	46294	0	0	0,00	0	0	0,00	0	0	0,00	0	0	0,00	0	0	0,00	0	0	0,00	0	0	0,00	2	2	15,15
254565307	C4QW60	CIT1	hypothetical protein	8,56	51855	0	0	0,00	0	0	0,00	2	2	15,15	1	5	20,19	1	1	10,15	2	3	20,15	2	24	94,21	2	13	55,20
254565311	C4QW62	PAS_chr1-1_0123	Putative sensor/transporter protein involved in cell wall biogenesis	6,63	107412	0	0	0,00	0	0	0,00	0	0	0,00	0	0	0,00	0	0	0,00	1	1	10,13	0	0	0,00	0	0	0,00
254565317	C4QW65	PP7435_Chr1-0433	hypothetical protein	4,82	42238	0	0	0,00	0	0	0,00	0	0	0,00	0	0	0,00	0	0	0,00	0	0	0,00	1	1	10,13	0	0	0,00
254565321	C4QW67	PP7435_Chr1-0435	hypothetical protein	4,88	141140	0	0	0,00	0	0	0,00	0	0	0,00	0	0	0,00	0	0	0,00	0	0	0,00	2	3	10,18	0	0	0,00
254565327	C4QW70	PP7435_Chr1-0438	hypothetical protein	7,35	21809	2	2	10,15	0	0	0,00	0	0	0,00	1	1	10,15	0	0	0,00	0	0	0,00	0	0	0,00	0	0	0,00
254565333	C4QW73	PP7435_Chr1-0442	Purine nucleoside phosphorylase, specifically metabolizes inosine & guanosine nucleosides	5,42	32777	0	0	0,00	0	0	0,00	0	0	0,00	0	0	0,00	0	0	0,00	0	0	0,00	1	1	18,14	0	0	0,00
254565341	C4QW77	DLT1	Protein of unknown function, mutant sensitive to 6-azauracil (6AU) and mycophenolic acid (MPA)	9,80	28503	1	1	10,13	0	0	0,00	0	0	0,00	0	0	0,00	0	0	0,00	0	0	0,00	0	0	0,00	0	0	0,00
254565375	C4QW94	PP7435_Chr1-0463	Serine/threonine protein phosphatase Z, isoform of Ppz2p	6,20	54213	0	0	0,00	0	0	0,00	2	1	10,12	0	0	0,00	0	0	0,00	0	0	0,00	0	0	0,00	0	0	0,00
254565391	C4QWA2	PP7435_Chr1-0470	Protein disulfide isomerase, multifunctional protein resident in the endoplasmic reticulum lumen	5,88	41938	0	0	0,00	0	0	0,00	0	0	0,00	1	1	10,15	2	1	10,16	1	1	10,14	1	1	10,12	0	0	0,00
254565433	C4QWC3	PP7435_Chr1-0490	Essential beta'-coat protein of the COPI coatomer, involved in ER-to-Golgi and Golgi-to-ER transport	4,58	100601	0	0	0,00	0	0	0,00	1	1	10,13	0	0	0,00	0	0	0,00	0	0	0,00	0	0	0,00	0	0	0,00
254565441	C4QWC7		Gamma glutamylcysteine synthetase catalyzes the first step in glutathione (GSH) biosynthesis	5,44	75237	0	0	0,00	0	0	0,00	0	0	0,00	0	0	0,00	0	0	0,00	0	0	0,00	1	1	10,12	1	2	10,14
254565451	C4QWD2	PP7435_Chr1-0499	60S ribosomal protein L12	9,93	17743	2	25	50,20	2	24	50,23	2	57	60,23	2	24	45,21	2	34	60,22	2	29	40,23	2	39	35,24	2	26	50,23
254565453	C4QWD3	PAS_chr1-1_0190	Non-catalytic subunit of N-terminal acetyltransferase of the NatC type	5,43	87232	0	0	0,00	0	0	0,00	0	0	0,00	0	0	0,00	2	1	10,15	1	1	10,11	0	0	0,00	0	0	0,00
254565455	C4QWD4	ATP5	Subunit 5 of the stator stalk of mitochondrial F1F0 ATP synthase	9,59	21709	0	0	0,00	0	0	0,00	0	0	0,00	0	0	0,00	0	0	0,00	0	0	0,00	1	1	10,17	0	0	0,00
254565463	C4QWD8	VMA8	Subunit D of the eight-subunit V1 peripheral membrane domain of the vacuolar H+-ATPase (V-ATPase)	5,33	32859	0	0	0,00	0	0	0,00	0	0	0,00	0	0	0,00	0	0	0,00	0	0	0,00	1	1	10,11	0	0	0,00
254565465	C4QWD9	PP7435_Chr1-0506	Component of the spliceosome complex involved in pre-mRNA splicing	4,89	94874	0	0	0,00	0	0	0,00	1	1	10,19	0	0	0,00	0	0	0,00	0	0	0,00	0	0	0,00	0	0	0,00
254565475	C4QWE4	AAT1	Cytosolic aspartate aminotransferase	6,78	47734	2	18	55,23	2	16	70,23	2	11	35,24	1	1	10,20	2	9	45,22	2	5	25,18	2	20	35,26	2	10	30,26
254565491	C4QWF2	PP7435_Chr1-0519	Hexameric DNA polymerase alpha-associated DNA helicase A involved in lagging strand DNA synthesis	5,86	81750	0	0	0,00	0	0	0,00	0	0	0,00	1	1	10,17	0	0	0,00	0	0	0,00	0	0	0,00	0	0	0,00
254565493	C4QWF3	ATP7	hypothetical protein	7,63	19659	2	1	10,14	1	1	10,13	1	1	10,13	0	0	0,00	2	2	15,16	1	1	10,12	2	4	15,19	2	1	10,16
254565511	C4QWG2	PP7435_Chr1-0530	Processing alpha glucosidase I, ER type II integral membrane N-glycoprotein	4,80	91703	0	0	0,00	0	0	0,00	0	0	0,00	0	0	0,00	0	0	0,00	0	0	0,00	2	1	10,22	0	0	0,00
254565513	C4QWG3	PP7435_Chr1-0531	60S ribosomal protein L8	10,00	26790	0	0	0,00	0	0	0,00	0	0	0,00	1	1	10,12	0	0	0,00	0	0	0,00	0	0	0,00	0	0	0,00
254565517	C4QWG5	SBP1	hypothetical protein	5,51	38594	0	0	0,00	0	0	0,00	1	1	10,19	0	0	0,00	2	3	15,16	0	0	0,00	1	1	10,16	1	1	10,14
254565519	C4QWG6	RpL8	60S ribosomal protein L2	11,60	27175	1	1	10,17	0	0	0,00	0	0	0,00	0	0	0,00	0	0	0,00	0	0	0,00	0	0	0,00	0	0	0,00
254565527	C4QWH0	PAS_chr1-1_0224	Nuclear pore protein that is part of the evolutionarily conserved Nup84p complex	5,24	35624	0	0	0,00	0	0	0,00	0	0	0,00	0	0	0,00	1	1	10,23	0	0	0,00	0	0	0,00	0	0	0,00
254565531	C4QWH2	PRB2	Vacuolar proteinase B (yscB), a serine protease of the subtilisin family	5,93	59069	1	1	10,12	0	0	0,00	0	0	0,00	0	0	0,00	0	0	0,00	0	0	0,00	0	0	0,00	0	0	0,00
254565545	C4QWH9	IDP1	Mitochondrial NADP-specific isocitrate dehydrogenase, catalyzes the oxidation of isocitrate	8,31	48460	2	9	25,19	2	11	40,19	2	4	10,18	2	3	15,15	2	5	20,16	2	3	10,15	2	7	30,16	2	10	20,17
254565559	C4QWI6	PAS_chr1-1_0486	hypothetical protein	4,49	22682	0	0	0,00	0	0	0,00	0	0	0,00	0	0	0,00	0	0	0,00	0	0	0,00	1	1	10,15	0	0	0,00
254565587	C4QWK0	PP7435_Chr1-0565	Member of the DRG family of GTP-binding proteins	8,46	40670	0	0	0,00	0	0	0,00	0	0	0,00	1	1	10,13	0	0	0,00	1	1	10,11	0	0	0,00	0	0	0,00
254565589	C4QWK1	PP7435_Chr1-0566	GTPase	5,86	115512	0	0	0,00	0	0	0,00	0	0	0,00	0	0	0,00	0	0	0,00	0	0	0,00	2	1	10,14	0	0	0,00
254565607	C4QWL0	FUR1	Uracil phosphoribosyltransferase, synthesizes UMP from uracil	8,19	24385	0	0	0,00	0	0	0,00	0	0	0,00	0	0	0,00	1	1	10,15	1	1	10,10	0	0	0,00	0	0	0,00
254565617	C4QWL5	PPIB	Peptidyl-prolyl cis-trans isomerase (cyclophilin) of the endoplasmic reticulum	4,59	24660	2	2	15,18	2	2	15,18	2	3	25,20	1	1	10,17	2	4	25,21	1	1	10,19	2	11	25,23	1	3	20,21
254565637	C4QWM5	PP7435_Chr1-0593	hypothetical protein	4,76	22613	1	1	10,15	1	2	20,14	0	0	0,00	0	0	0,00	0	0	0,00	0	0	0,00	0	0	0,00	0	0	0,00
254565639	C4QWM6	SOL1	Protein with a possible role in tRNA export	6,37	32246	0	0	0,00	0	0	0,00	0	0	0,00	0	0	0,00	0	0	0,00	0	0	0,00	2	2	15,21	0	0	0,00
254565645	C4QWM9	XYL2	hypothetical protein	5,44	37411	2	5	20,25	2	2	15,19	0	0	0,00	0	0	0,00	0	0	0,00	0	0	0,00	0	0	0,00	1	1	10,12
254565657	C4QWN5	THI73	Putative plasma membrane permease proposed to be involved in carboxylic acid uptake	8,90	55298	0	0	0,00	1	1	10,18	0	0	0,00	0	0	0,00	0	0	0,00	0	0	0,00	0	0	0,00	0	0	0,00
254565661	C4QWN7	PP7435_Chr1-0604	F-box protein of unknown function	7,90	48100	1	1	10,14	0	0	0,00	0	0	0,00	0	0	0,00	0	0	0,00	0	0	0,00	0	0	0,00	0	0	0,00
254565681	C4QWP7	PP7435_Chr1-0613	Alpha subunit of COPI vesicle coatomer complex	5,84	136632	0	0	0,00	0	0	0,00	0	0	0,00	0	0	0,00	0	0	0,00	0	0	0,00	1	1	10,14	0	0	0,00
254565693	C4QWQ3	PAS_chr1-1_0299	Essential, non-ATPase regulatory subunit of the 26S proteasome lid	5,68	51088	0	0	0,00	0	0	0,00	0	0	0,00	0	0	0,00	1	1	10,17	0	0	0,00	2	8	10,22	1	1	10,15
254565699	C4QWQ6	PP7435_Chr1-0622	60S ribosomal protein L35	11,10	13866	2	2	10,21	1	1	10,22	2	4	10,22	0	0	0,00	0	0	0,00	0	0	0,00	0	0	0,00	0	0	0,00
254565701	C4QWQ7	UFD2	Ubiquitin chain assembly factor (E4) that cooperates with a ubiquitin-activating enzyme (E1)	5,50	109786	1	1	10,13	0	0	0,00	0	0	0,00	0	0	0,00	0	0	0,00	0	0	0,00	0	0	0,00	0	0	0,00
254565705	C4QWQ9	RDI1	Rho GDP dissociation inhibitor involved in the localization and regulation of Cdc42p	5,27	22337	0	0	0,00	0	0	0,00	0	0	0,00	0	0	0,00	0	0	0,00	0	0	0,00	1	1	10,18	0	0	0,00
254565713	C4QWR3	PP7435_Chr1-0630	Vacuolar ATPase V1 domain subunit A containing the catalytic nucleotide binding sites	5,05	63137	0	0	0,00	0	0	0,00	0	0	0,00	0	0	0,00	0	0	0,00	0	0	0,00	2	4	20,16	0	0	0,00
254565717	C4QWR5	PP7435_Chr1-0632	Putative protein of unknown function	6,36	39838	0	0	0,00	0	0	0,00	0	0	0,00	0	0	0,00	0	0	0,00	1	1	10,14	0	0	0,00	0	0	0,00
254565745	C4QWS9	PRD1	Zinc metalloendopeptidase, found in the cytoplasm and intermembrane space of mitochondria	5,70	83449	1	2	20,16	0	0	0,00	2	2	10,18	0	0	0,00	2	1	10,13	1	1	10,12	0	0	0,00	2	3	10,15
254565751	C4QWT2	PP7435_Chr1-0650	hypothetical protein	4,59	121302	0	0	0,00	0	0	0,00	1	1	10,13	0	0	0,00	0	0	0,00	0	0	0,00	0	0	0,00	0	0	0,00
254565783	C4QWU8	PP7435_Chr1-0662	60S ribosomal protein L5	8,70	34099	2	10	25,22	2	5	10,23	2	15	35,25	2	10	40,21	2	12	30,22	2	4	10,20	2	33	40,25	2	10	20,24
254565815	C4QWW4	PP7435_Chr1-0679	2-methylbutyraldehyde reductase, may be involved in isoleucine catabolism	5,40	34377	1	1	10,16	1	1	10,16	0	0	0,00	0	0	0,00	0	0	0,00	0	0	0,00	0	0	0,00	0	0	0,00
254565873	C4QWZ3	PAS_chr1-1_0389	Argininosuccinate lyase, catalyzes the final step in the arginine biosynthesis pathway	5,16	52280	0	0	0,00	0	0	0,00	1	1	10,13	0	0	0,00	0	0	0,00	0	0	0,00	0	0	0,00	0	0	0,00
254565899	C4QX06	PP7435_Chr1-0722	hypothetical protein	4,51	85173	2	1	10,17	2	1	10,17	0	0	0,00	0	0	0,00	0	0	0,00	0	0	0,00	0	0	0,00	0	0	0,00
254565909	C4QX11	PP7435_Chr1-0726	Putative protein of unknown function	6,20	21141	1	1	10,11	0	0	0,00	0	0	0,00	0	0	0,00	0	0	0,00	0	0	0,00	0	0	0,00	0	0	0,00
254565913	C4QX13	PP7435_Chr1-0728	Karyopherin beta, mediates nuclear import of ribosomal proteins	4,53	123038	0	0	0,00	0	0	0,00	0	0	0,00	0	0	0,00	0	0	0,00	0	0	0,00	1	1	10,18	0	0	0,00
254565947	C4QX30	PP7435_Chr1-0744	hypothetical protein	5,60	69255	2	1	10,16	0	0	0,00	0	0	0,00	0	0	0,00	0	0	0,00	0	0	0,00	0	0	0,00	0	0	0,00
254565949	C4QX31	PP7435_Chr1-0745	hypothetical protein	10,22	26545	2	1	10,11	1	1	10,13	0	0	0,00	0	0	0,00	0	0	0,00	0	0	0,00	0	0	0,00	0	0	0,00
254565955	C4QX34	ADE4	Phosphoribosylpyrophosphate amidotransferase	6,60	59850	0	0	0,00	0	0	0,00	1	1	10,15	0	0	0,00	2	2	15,14	2	1	10,17	0	0	0,00	0	0	0,00
254565959	C4QX36	ILV5	Acetohydroxyacid reductoisomerase	8,08	44265	2	14	40,22	2	16	40,20	2	19	75,18	2	2	19,14	2	17	50,22	2	13	55,17	2	50	95,25	2	25	65,24
254565961	C4QX37	PP7435_Chr1-0751	Mitochondrial peroxiredoxin (1-Cys Prx) with thioredoxin peroxidase activity	5,71	25102	2	3	15,17	2	3	10,18	2	1	14,23	2	2	14,21	2	3	20,24	2	2	10,19	2	3	15,18	2	2	15,19
254565973	C4QX43	rps8	40S ribosomal protein S8	10,69	22443	0	0	0,00	0	0	0,00	0	0	0,00	0	0	0,00	0	0	0,00	1	1	10,13	2	2	15,15	0	0	0,00
254565975	C4QX44	PP7435_Chr1-0758	hypothetical protein	6,38	11826	2	5	30,22	2	2	15,19	0	0	0,00	0	0	0,00	0	0	0,00	0	0	0,00	0	0	0,00	0	0	0,00
254565989	C4QX51	PP7435_Chr1-0766	Saccharopine dehydrogenase (NAD+, L-lysine-forming)	5,48	40755	0	0	0,00	2	1	10,13	1	1	10,13	0	0	0,00	1	2	20,15	2	1	10,13	1	2	10,13	0	0	0,00
254565991	C4QX52	PDX3	Pyridoxine (pyridoxamine) phosphate oxidase, has homologs in E. coli and Myxococcus xanthus	6,25	26056	2	1	10,16	2	1	10,15	0	0	0,00	0	0	0,00	0	0	0,00	0	0	0,00	0	0	0,00	0	0	0,00
254566021	C4QX67	DHH1	Cytoplasmic DExD/H-box helicase	8,75	53421	0	0	0,00	0	0	0,00	0	0	0,00	0	0	0,00	0	0	0,00	1	1	10,12	1	1	10,18	0	0	0,00
254566029	C4QX71	PRW1	hypothetical protein	8,82	60164	0	0	0,00	0	0	0,00	1	1	10,16	0	0	0,00	0	0	0,00	0	0	0,00	0	0	0,00	0	0	0,00
254566031	C4QX72	PAS_chr1-4_0011	hypothetical protein	6,02	54426	0	0	0,00	0	0	0,00	0	0	0,00	0	0	0,00	1	1	10,12	0	0	0,00	0	0	0,00	0	0	0,00
254566053	C4QX83	PP7435_Chr1-0797	Mitochondrial RNA polymerase	8,91	150453	0	0	0,00	0	0	0,00	0	0	0,00	1	1	10,13	0	0	0,00	0	0	0,00	0	0	0,00	0	0	0,00
254566055	C4QX84	SNF7	One of four subunits of the endosomal sorting complex required for transport III (ESCRT-III)	4,63	26148	2	1	10,19	0	0	0,00	2	3	20,21	0	0	0,00	2	1	10,20	0	0	0,00	2	1	10,19	2	1	10,18
254566071	C4QX92	PP7435_Chr1-0807	Putative protein of unknown function	5,46	35912	0	0	0,00	0	0	0,00	0	0	0,00	0	0	0,00	0	0	0,00	0	0	0,00	2	2	10,21	0	0	0,00
254566081	C4QX97	PP7435_Chr1-0812	hypothetical protein	9,80	12797	1	1	10,13	0	0	0,00	0	0	0,00	0	0	0,00	0	0	0,00	0	0	0,00	0	0	0,00	0	0	0,00
254566087	C4QXA0	PP7435_Chr1-0815	Glucose-6-phosphate 1-epimerase (hexose-6-phosphate mutarotase)	4,86	33898	0	0	0,00	0	0	0,00	0	0	0,00	0	0	0,00	0	0	0,00	0	0	0,00	2	4	10,23	0	0	0,00
254566089	C4QXA1	SBA1	Co-chaperone that binds to and regulates Hsp90 family chaperones	4,11	22441	0	0	0,00	0	0	0,00	2	9	60,18	2	7	45,17	2	1	10,16	2	9	35,18	2	1	10,19	2	4	35,16
254566097	C4QXA5	PFK1	Beta subunit of heterooctameric phosphofructokinase involved in glycolysis	6,26	101361	0	0	0,00	0	0	0,00	0	0	0,00	0	0	0,00	0	0	0,00	0	0	0,00	2	3	15,17	1	3	20,13
254566101	C4QXA7	HOM6	Homoserine dehydrogenase (L-homoserine:NADP oxidoreductase), dimeric enzyme	5,04	38880	0	0	0,00	0	0	0,00	0	0	0,00	0	0	0,00	1	1	10,15	0	0	0,00	2	2	15,15	0	0	0,00
254566125	C4QXB9	PP7435_Chr1-0835	hypothetical protein	5,60	54679	0	0	0,00	0	0	0,00	1	1	10,21	0	0	0,00	1	1	10,14	0	0	0,00	2	3	10,22	2	2	10,24
254566127	C4QXC0	PAS_chr1-4_0063	G-protein beta subunit and guanine nucleotide dissociation inhibitor for Gpa2p	6,33	34652	2	5	30,19	2	11	35,19	2	4	10,21	0	0	0,00	2	4	10,20	0	0	0,00	2	2	10,20	2	3	10,18
254566141	C4QXC7	SOD2	Mitochondrial superoxide dismutase, protects cells against oxygen toxicity	8,79	25140	0	0	0,00	0	0	0,00	0	0	0,00	0	0	0,00	2	1	10,15	0	0	0,00	0	0	0,00	1	1	10,16
254566143	C4QXC8	AHA1	Co-chaperone that binds to Hsp82p and activates its ATPase activity	5,32	37440	0	0	0,00	0	0	0,00	0	0	0,00	0	0	0,00	0	0	0,00	0	0	0,00	1	1	10,21	0	0	0,00
254566149	C4QXD1	PP7435_Chr1-0845	L-rhamnonate dehydratase	5,70	46057	0	0	0,00	0	0	0,00	0	0	0,00	0	0	0,00	1	1	10,13	0	0	0,00	0	0	0,00	0	0	0,00
254566157	C4QXD5	PAS_chr1-4_0079	Protein of unknown function, affects chromosome stability when overexpressed	9,18	45793	0	0	0,00	0	0	0,00	0	0	0,00	0	0	0,00	0	0	0,00	0	0	0,00	1	1	10,11	0	0	0,00
254566163	C4QXD8	PP7435_Chr1-0852	Putative cytoplasmic protein of unknown function	5,69	57052	0	0	0,00	0	0	0,00	0	0	0,00	0	0	0,00	0	0	0,00	0	0	0,00	1	2	20,21	0	0	0,00
254566191	C4QXF2	DPS1	Cytoplasmic aspartyl-tRNA synthetase, homodimeric enzyme	5,98	62992	0	0	0,00	0	0	0,00	0	0	0,00	0	0	0,00	2	2	20,14	0	0	0,00	2	2	20,14	0	0	0,00
254566225	C4QXG9	SGTA	Glutamine-rich cytoplasmic protein of unknown function	4,61	36649	1	1	10,13	0	0	0,00	0	0	0,00	0	0	0,00	1	1	10,19	0	0	0,00	0	0	0,00	0	0	0,00
254566257	C4QXI5	PP7435_Chr1-0897	Heat shock protein Hsp90	4,68	80833	2	1	10,16	2	1	13,16	2	9	50,18	2	6	50,17	2	4	33,15	2	13	65,21	2	15	70,22	2	3	30,17
254566307	C4QXL0	PP7435_Chr1-0922	Alpha aminoadipate reductase	5,44	155859	0	0	0,00	0	0	0,00	0	0	0,00	0	0	0,00	1	1	10,13	0	0	0,00	2	1	10,15	1	1	10,16
254566317	C4QXL5	CLC1	Clathrin light chain	4,36	24818	2	7	10,25	2	5	10,26	2	8	15,30	2	4	10,25	2	6	15,29	2	2	10,28	2	3	15,28	2	3	10,29
254566327	C4QXM0	PP7435_Chr1-0932	Putative protein of unknown function	9,55	11129	1	1	10,18	0	0	0,00	2	5	30,19	2	2	10,18	2	5	25,20	2	3	25,15	2	14	65,20	2	21	90,20
254566341	C4QXM7	PP7435_Chr1-0941	Putative protein of unknown function	4,27	33548	0	0	0,00	0	0	0,00	0	0	0,00	0	0	0,00	0	0	0,00	0	0	0,00	0	0	0,00	2	3	10,27
254566351	C4QXN2	PP7435_Chr1-0946	hypothetical protein	6,34	57077	0	0	0,00	0	0	0,00	0	0	0,00	0	0	0,00	0	0	0,00	0	0	0,00	2	3	20,16	0	0	0,00
254566355	C4QXN4	PP7435_Chr1-0948	hypothetical protein	4,82	17859	0	0	0,00	0	0	0,00	1	1	10,13	0	0	0,00	2	18	44,31	0	0	0,00	2	11	65,25	2	4	25,17
254566357	C4QXN5	IML2	hypothetical protein	8,23	83805	0	0	0,00	0	0	0,00	0	0	0,00	0	0	0,00	1	1	10,12	0	0	0,00	0	0	0,00	0	0	0,00
254566369	C4QXP1	NMA1	Nicotinic acid mononucleotide adenylyltransferase, involved in pathways of NAD biosynthesis	6,86	46595	1	1	10,10	0	0	0,00	0	0	0,00	0	0	0,00	0	0	0,00	0	0	0,00	0	0	0,00	0	0	0,00
254566377	C4QXP5	PP7435_Chr1-0961	Subunit of the ARP2/3 complex	6,64	19877	0	0	0,00	0	0	0,00	0	0	0,00	0	0	0,00	0	0	0,00	0	0	0,00	1	1	10,15	0	0	0,00

254566395	C4QXQ4	PP7435_Chr1-0970	Polyamine transporter that recognizes spermine, putrescine, and spermidine	5,93	63670	0	0	0,00	0	0	0,00	0	0	0,00	0	0	0,00	0	0	0,00	0	0	0,00	1	1	10,17	0	0	0,00
254566397	C4QXQ5	PAS_chr1-4_0195	Putative sulfate permease	8,13	72880	0	0	0,00	0	0	0,00	1	1	10,13	0	0	0,00	0	0	0,00	0	0	0,00	0	0	0,00	0	0	0,00
254566413	C4QXR3	PAS_chr1-4_0203	Component of the evolutionarily conserved kinetochore-associated Ndc80 complex	5,21	79506	0	0	0,00	0	0	0,00	0	0	0,00	0	0	0,00	0	0	0,00	0	0	0,00	0	0	0,00	1	1	10,13
254566415	C4QXR4	XPB2	Component of the holoenzyme form of RNA polymerase transcription factor TFIIH	6,00	92752	0	0	0,00	0	0	0,00	1	1	10,13	0	0	0,00	1	1	10,19	0	0	0,00	2	1	10,19	0	0	0,00
254566417	C4QXR5	SMT3	Ubiquitin-like protein of the SUMO family	5,01	11127	2	5	15,29	2	4	10,29	2	6	20,29	1	2	20,23	2	5	20,31	2	2	10,18	2	3	10,34	2	1	10,27
254566423	C4QXR8	GRX5	Hydroperoxide and superoxide-radical responsive glutathione-dependent oxidoreductase	4,69	18297	1	1	10,20	0	0	0,00	2	2	10,18	1	1	10,15	1	1	10,22	1	1	10,11	2	2	10,23	1	1	10,14
254566427	C4QXS0	PAS_chr1-4_0208	RNA helicase	9,27	23834	0	0	0,00	0	0	0,00	0	0	0,00	0	0	0,00	0	0	0,00	0	0	0,00	2	1	10,13	0	0	0,00
254566433	C4QXS3	PAS_chr1-4_0210	Core component of the signal recognition particle (SRP) ribonucleoprotein (RNP) complex	9,24	71360	0	0	0,00	0	0	0,00	1	1	10,13	0	0	0,00	0	0	0,00	0	0	0,00	0	0	0,00	0	0	0,00
254566447	C4QXT0	ARO4	3-deoxy-D-arabino-heptulosonate-7-phosphate (DAHP) synthase	5,84	38869	0	0	0,00	0	0	0,00	0	0	0,00	0	0	0,00	0	0	0,00	0	0	0,00	1	1	10,24	0	0	0,00
254566451	C4QXT2	BDF1	Protein involved in transcription initiation at TATA-containing promoters	5,46	72259	0	0	0,00	0	0	0,00	0	0	0,00	0	0	0,00	0	0	0,00	0	0	0,00	1	1	10,15	1	1	10,15
254566479	C4QXU6	HIS1	ATP phosphoribosyltransferase (hexameric enzyme) catalyzes the first step in histidine biosynthesis	5,10	32488	1	1	10,19	1	1	10,14	1	1	10,13	0	0	0,00	1	1	10,14	0	0	0,00	0	0	0,00	2	1	10,17
254566485	C4QXU9	PAS_chr1-4_0237	hypothetical protein	9,52	10217	0	0	0,00	0	0	0,00	0	0	0,00	0	0	0,00	2	1	10,21	0	0	0,00	0	0	0,00	0	0	0,00
254566487	C4QXV0	PAS_chr1-4_0238	Mitochondrial protein involved in maintenance of the mitochondrial genome	9,14	15871	2	4	15,23	2	5	20,23	2	1	10,22	0	0	0,00	2	3	30,22	1	1	10,17	2	1	10,22	2	1	10,19
254566497	C4QXV5	ILV1	Threonine deaminase, catalyzes the first step in isoleucine biosynthesis	7,95	61075	2	2	15,15	2	3	10,15	0	0	0,00	0	0	0,00	0	0	0,00	0	0	0,00	0	0	0,00	0	0	0,00
254566509	C4QXW1	PAS_chr1-4_0249	Acetyl-CoA carboxylase, biotin containing enzyme	5,92	247803	1	2	10,19	2	2	10,21	2	4	15,20	2	2	10,18	2	4	20,20	1	1	10,13	2	4	25,20	1	2	20,19
254566513	C4QXW3	PP7435_Chr1-1031	hypothetical protein	6,30	46664	1	1	10,18	0	0	0,00	0	0	0,00	0	0	0,00	1	1	10,16	0	0	0,00	0	0	0,00	0	0	0,00
254566517	C4QXW5	PP7435_Chr1-1033	Dihydrolipoamide dehydrogenase (E3)-binding protein (E3BP)	5,10	41704	1	1	10,12	1	1	10,15	2	1	10,19	0	0	0,00	2	2	15,24	0	0	0,00	2	4	25,24	2	2	10,23
254566531	C4QXX2	CKA2	Alpha' catalytic subunit of casein kinase 2	8,50	38810	2	2	10,21	1	1	10,20	0	0	0,00	0	0	0,00	0	0	0,00	0	0	0,00	0	0	0,00	0	0	0,00
254566535	C4QXX4	ERB1	hypothetical protein	4,87	92623	0	0	0,00	0	0	0,00	1	1	10,16	1	1	10,16	0	0	0,00	0	0	0,00	0	0	0,00	0	0	0,00
254566539	C4QXX6	PP7435_Chr1-1045	hypothetical protein	6,27	27339	0	0	0,00	0	0	0,00	0	0	0,00	0	0	0,00	0	0	0,00	0	0	0,00	1	1	10,12	0	0	0,00
254566545	C4QXX9	RUD3	Golgi matrix protein involved in the structural organization of the cis-Golgi	4,39	70666	2	1	10,15	1	1	10,20	0	0	0,00	0	0	0,00	0	0	0,00	0	0	0,00	0	0	0,00	0	0	0,00
254566553	C4QXY3	PAS_chr1-4_0272	GrpE protein homolog, mitochondrial	8,44	33207	0	0	0,00	0	0	0,00	2	2	15,15	1	1	10,16	2	1	10,16	2	2	15,18	2	5	20,18	2	7	25,18
254566567	C4QXZ0	KES1	Member of the oxysterol binding protein family, which includes seven yeast homologs	8,66	47240	0	0	0,00	0	0	0,00	2	1	10,15	0	0	0,00	2	1	10,21	0	0	0,00	2	1	10,18	0	0	0,00
254566601	C4QY07	PGK1	3-phosphoglycerate kinase	8,72	44058	2	22	85,18	2	24	50,18	2	5	45,22	2	1	10,16	2	4	35,16	2	3	25,18	2	8	69,23	1	1	10,15
254566607	C4QY10	PP7435_Chr1-1078	Alpha subunit of fatty acid synthetase	5,17	206000	1	1	10,17	2	3	30,17	2	4	35,18	2	3	30,19	2	4	30,18	2	2	15,16	2	7	40,19	1	1	10,15
254566611	C4QY12	PP7435_Chr1-1080	Suppressor protein STM1	10,21	29685	2	1	10,20	1	1	10,16	2	4	30,17	0	0	0,00	2	4	39,28	0	0	0,00	1	1	10,15	2	2	20,28
254566615	C4QY14	PP7435_Chr1-1082	hypothetical protein	6,39	49877	0	0	0,00	0	0	0,00	0	0	0,00	0	0	0,00	2	1	10,18	0	0	0,00	0	0	0,00	0	0	0,00
254566619	C4QY16	NDE1	Mitochondrial external NADH dehydrogenase, a type II NAD(P)H:quinone oxidoreductase	9,19	62672	0	0	0,00	0	0	0,00	0	0	0,00	0	0	0,00	1	1	10,14	0	0	0,00	1	1	10,15	0	0	0,00
254566631	C4QY22	PP7435_Chr1-1090	Acetyl-CoA C-acetyltransferase (acetoacetyl-CoA thiolase), cytosolic enzyme	6,10	41687	1	1	10,20	2	2	10,18	2	1	10,20	0	0	0,00	0	0	0,00	0	0	0,00	0	0	0,00	0	0	0,00
254566651	C4QY32	PP7435_Chr1-1100	Farnesyl pyrophosphate synthetase	5,09	41009	0	0	0,00	0	0	0,00	1	1	10,15	0	0	0,00	0	0	0,00	0	0	0,00	2	8	15,22	0	0	0,00
254566657	C4QY35	RET3	Zeta subunit of the coatomer complex (COPI), which coats Golgi-derived transport vesicles	4,53	21236	0	0	0,00	0	0	0,00	0	0	0,00	0	0	0,00	0	0	0,00	0	0	0,00	1	2	20,17	0	0	0,00
254566683	C4QY48	PAS_chr1-4_0671	hypothetical protein	6,32	58523	0	0	0,00	0	0	0,00	0	0	0,00	0	0	0,00	1	1	10,13	0	0	0,00	0	0	0,00	0	0	0,00
254566719	C4QY66	PP7435_Chr1-1132	Putative metalloprotease	5,10	77665	2	1	10,13	2	1	10,12	1	1	10,13	0	0	0,00	2	3	25,16	0	0	0,00	2	1	10,16	2	4	40,21
254566731	C4QY72	PAS_chr1-4_0353	40S ribosomal protein S19	9,63	16688	0	0	0,00	0	0	0,00	0	0	0,00	0	0	0,00	2	1	10,16	0	0	0,00	0	0	0,00	0	0	0,00
254566735	C4QY74	HRP1	Subunit of cleavage factor I	4,90	51234	2	1	10,19	1	1	10,19	2	1	10,24	1	1	10,24	0	0	0,00	2	2	15,22	0	0	0,00	0	0	0,00
254566743	C4QY78	PAS_chr1-4_0358	Protein associated with the mitochondrial nucleoid	5,26	17231	0	0	0,00	0	0	0,00	0	0	0,00	0	0	0,00	0	0	0,00	1	1	10,19	2	3	10,18	2	5	20,21
254566751	C4QY82	PP7435_Chr1-1147	Putative metalloprotease	7,72	72342	1	1	10,15	0	0	0,00	0	0	0,00	0	0	0,00	0	0	0,00	0	0	0,00	0	0	0,00	0	0	0,00
254566769	C4QY91	DBI	hypothetical protein	4,67	9814	0	0	0,00	2	1	10,14	0	0	0,00	0	0	0,00	0	0	0,00	0	0	0,00	0	0	0,00	0	0	0,00
254566771	C4QY92	NUFM	hypothetical protein	4,92	15543	0	0	0,00	0	0	0,00	1	1	10,20	0	0	0,00	0	0	0,00	0	0	0,00	2	2	15,18	0	0	0,00
254566779	C4QY96	PP7435_Chr1-1161	Subunit of an adoMet-dependent tRNA methyltransferase (MTase) complex (Trm11p-Trm112p)	4,62	14541	0	0	0,00	0	0	0,00	2	1	10,17	0	0	0,00	2	1	10,15	0	0	0,00	0	0	0,00	1	1	10,17
254566843	C4QYC8	PAS_chr1-4_0403	Essential evolutionarily-conserved nucleolar protein component of the box C/D snoRNP complexes	9,50	55398	1	1	10,15	0	0	0,00	2	1	10,18	0	0	0,00	2	1	10,17	0	0	0,00	0	0	0,00	0	0	0,00
254566845	C4QYC9	PAS_chr1-4_0404	hypothetical protein	4,84	28332	1	1	10,14	0	0	0,00	1	2	20,18	1	1	10,18	2	3	15,17	0	0	0,00	2	5	30,17	2	3	20,16
254566849	C4QYD1	COQ9	Protein required for ubiquinone (coenzyme Q) biosynthesis and respiratory growth	7,00	32419	0	0	0,00	0	0	0,00	0	0	0,00	0	0	0,00	0	0	0,00	0	0	0,00	1	1	10,15	0	0	0,00
254566881	C4QYE7	PAS_chr1-4_0421	Homocitrate synthase isozyme, catalyzes the condensation of acetyl-CoA and alpha-ketoglutarate	5,61	48995	0	0	0,00	0	0	0,00	0	0	0,00	0	0	0,00	0	0	0,00	0	0	0,00	0	0	0,00	2	1	10,12
254566893	C4QYF3	BGL2	Endo-beta-1,3-glucanase, major protein of the cell wall, involved in cell wall maintenance	3,91	33907	2	3	15,18	2	1	14,18	0	0	0,00	0	0	0,00	0	0	0,00	0	0	0,00	2	11	30,24	2	10	40,23
254566897	C4QYF5	BFR1	Component of mRNP complexes associated with polyribosomes	9,46	58239	0	0	0,00	0	0	0,00	1	1	18,15	0	0	0,00	2	1	14,14	0	0	0,00	1	1	18,15	1	1	10,14
254566899	C4QYF6	IDI1	Isopentenyl diphosphate:dimethylallyl diphosphate isomerase (IPP isomerase)	5,06	32379	1	1	10,17	2	2	15,15	1	1	10,16	0	0	0,00	0	0	0,00	1	1	10,15	1	1	10,18	1	1	10,18
254566925	C4QYG9	PAS_chr1-4_0441	Peroxisomal primary amine oxidase	5,72	80481	0	0	0,00	0	0	0,00	0	0	0,00	0	0	0,00	0	0	0,00	0	0	0,00	2	5	24,22	0	0	0,00
254566933	C4QYH3	PP7435_Chr1-1240	Delta subunit of the central stalk of mitochondrial F1F0 ATP synthase	4,73	16963	2	4	20,14	2	3	25,15	0	0	0,00	0	0	0,00	2	6	40,18	0	0	0,00	2	20	60,19	2	17	47,19
254566947	C4QYI0	SEC6	Essential 88kDa subunit of the exocyst complex	4,61	91652	0	0	0,00	0	0	0,00	1	1	10,14	0	0	0,00	0	0	0,00	0	0	0,00	1	1	10,17	0	0	0,00
254566961	C4QYI7	ilvD	Dihydroxyacid dehydratase	6,67	65865	2	3	20,17	2	3	25,16	2	3	20,20	1	1	10,14	2	3	20,20	2	4	20,17	2	4	10,27	2	4	25,25
254566971	C4QYJ2	SES1	Cytosolic seryl-tRNA synthetase, class II aminoacyl-tRNA synthetase	5,66	52822	0	0	0,00	0	0	0,00	2	8	35,22	2	2	10,17	2	7	35,21	2	4	10,21	2	9	40,21	2	5	15,21
254566975	C4QYJ4	ATP3	Gamma subunit of the F1 sector of mitochondrial F1F0 ATP synthase	8,40	31603	2	1	10,18	2	2	10,19	2	1	10,21	0	0	0,00	2	3	10,21	1	1	10,15	2	5	20,23	2	1	10,20
254566987	C4QYK0	YST1	40S ribosomal protein S0	4,40	29186	0	0	0,00	0	0	0,00	0	0	0,00	0	0	0,00	0	0	0,00	2	1	10,11	2	1	10,13	0	0	0,00
254567001	C4QYK7	NAS6	Regulatory, non-ATPase subunit of the 26S proteasome	5,07	25022	0	0	0,00	0	0	0,00	0	0	0,00	0	0	0,00	1	1	10,13	0	0	0,00	0	0	0,00	0	0	0,00
254567005	C4QYK9	ZPR1	Essential protein with two zinc fingers, present in the nucleus of growing cells	4,68	53012	0	0	0,00	0	0	0,00	0	0	0,00	0	0	0,00	0	0	0,00	0	0	0,00	2	1	10,18	0	0	0,00
254567025	C4QYL9	CYS3	Cystathionine gamma-lyase	5,86	42658	2	2	10,16	2	3	15,16	0	0	0,00	1	1	10,14	0	0	0,00	0	0	0,00	0	0	0,00	0	0	0,00
254567027	C4QYM0	PAS_chr1-4_0490	60S acidic ribosomal protein P2	3,73	12207	2	1	10,18	2	1	14,16	2	3	20,18	2	4	15,21	1	1	10,13	2	5	25,22	2	3	20,16	1	1	10,13
254567029	C4QYM1	PAS_chr1-4_0491	40S ribosomal protein S15	10,67	16333	2	4	10,13	2	2	10,13	0	0	0,00	0	0	0,00	0	0	0,00	0	0	0,00	0	0	0,00	0	0	0,00
254567053	C4QYN3	PAS_chr1-4_0503	Endosomal subunit of membrane-associated retromer complex required for retrograde transport	5,00	97336	0	0	0,00	0	0	0,00	0	0	0,00	0	0	0,00	1	1	10,14	0	0	0,00	0	0	0,00	0	0	0,00
254567081	C4QYP7	PAS_chr1-4_0519	Co-chaperone that stimulates the ATPase activity of Ssa1p	4,78	59199	0	0	0,00	0	0	0,00	1	1	10,12	0	0	0,00	0	0	0,00	0	0	0,00	0	0	0,00	0	0	0,00
254567099	C4QYQ6	PP7435_Chr1-1327	hypothetical protein	4,00	16946	0	0	0,00	0	0	0,00	2	1	10,24	2	1	10,24	0	0	0,00	0	0	0,00	0	0	0,00	0	0	0,00
254567103	C4QYQ8	PP7435_Chr1-1329	Subunit of the heterodimeric FACT complex (Spt16p-Pob3p)	4,96	114767	0	0	0,00	0	0	0,00	1	1	10,13	0	0	0,00	0	0	0,00	0	0	0,00	0	0	0,00	0	0	0,00
254567105	C4QYQ9	PAS_chr1-4_0530	hypothetical protein	9,30	55943	2	1	10,18	0	0	0,00	0	0	0,00	0	0	0,00	0	0	0,00	0	0	0,00	0	0	0,00	0	0	0,00
254567113	C4QYR3	leuS	Mitochondrial leucyl-tRNA synthetase	6,07	99099	0	0	0,00	0	0	0,00	0	0	0,00	0	0	0,00	1	1	14,13	0	0	0,00	0	0	0,00	0	0	0,00
254567123	C4QYR8	PAS_chr1-4_0688	mitochondrial 37S ribosomal protein MRPS35	10,10	37182	2	2	10,16	0	0	0,00	1	2	10,19	0	0	0,00	2	4	20,25	0	0	0,00	2	3	10,23	0	0	0,00
254567139	C4QYS6	MRI1	Methylthioribose-1-phosphate isomerase	5,44	45413	1	1	10,12	0	0	0,00	0	0	0,00	0	0	0,00	2	1	10,14	0	0	0,00	0	0	0,00	0	0	0,00
254567143	C4QYS8	Chc	Clathrin heavy chain, subunit of the major coat protein	4,85	187126	0	0	0,00	0	0	0,00	0	0	0,00	0	0	0,00	0	0	0,00	1	1	10,15	2	3	20,19	0	0	0,00
254567169	C4QYU1	TAH18	NADPH reductase	6,39	65641	0	0	0,00	0	0	0,00	1	1	10,19	0	0	0,00	2	1	10,20	0	0	0,00	1	1	10,18	2	1	10,19
254567173	C4QYU3	HXK2	Hexokinase-2	5,39	55359	2	17	50,26	2	22	55,26	2	4	15,25	2	4	25,23	2	9	40,24	2	4	40,24	2	5	30,25	2	15	55,27
254567185	C4QYU9	PRE4	Beta 7 subunit of the 20S proteasome	5,80	29705	1	1	10,18	1	1	10,13	0	0	0,00	0	0	0,00	0	0	0,00	2	1	10,15	0	0	0,00	1	1	10,14
254567189	C4QYV1	SLB2	Primary component of eisosomes	4,88	35209	2	10	70,29	2	8	60,29	2	4	20,17	2	2	20,14	2	6	40,16	2	1	10,14	2	8	40,28	2	15	75,28
254567191	C4QYV2	PP7435_Chr1-1374	High-affinity glucose transporter of the major facilitator superfamily	6,92	59508	0	0	0,00	1	1	10,12	0	0	0,00	0	0	0,00	0	0	0,00	0	0	0,00	0	0	0,00	2	3	10,17
254567231	C4QYX2	PAS_chr1-4_0588	hypothetical protein	8,47	18991	0	0	0,00	0	0	0,00	0	0	0,00	0	0	0,00	0	0	0,00	0	0	0,00	1	1	10,19	1	3	20,21
254567233	C4QYX3	PAS_chr1-4_0589	40S ribosomal protein S2	10,38	28190	2	7	50,20	2	4	35,16	0	0	0,00	0	0	0,00	0	0	0,00	0	0	0,00	2	8	20,23	0	0	0,00
254567239	C4QYX6	ABC1	hypothetical protein	6,17	66422	0	0	0,00	0	0	0,00	0	0	0,00	0	0	0,00	0	0	0,00	0	0	0,00	2	2	10,18	0	0	0,00
254567243	C4QYX8	PDHbeta1	E1 beta subunit of the pyruvate dehydrogenase (PDH) complex	4,91	39557	2	1	10,12	0	0	0,00	0	0	0,00	0	0	0,00	0	0	0,00	0	0	0,00	2	1	10,15	1	1	10,15
254567253	C4QYY3	PP7435_Chr1-1407	Glutamyl-tRNA synthetase, cytoplasmic	6,34	82285	2	1	10,17	0	0	0,00	2	1	10,17	1	1	10,15	2	4	30,18	0	0	0,00	2	1	10,22	0	0	0,00
254567273	C4QYZ3	ARO8	Aromatic aminotransferase I, expression is regulated by general control of amino acid biosynthesis	5,23	54032	0	0	0,00	0	0	0,00	2	3	20,18	0	0	0,00	2	3	15,18	0	0	0,00	2	2	20,16	2	3	25,16
254567279	C4QYZ6	PP7435_Chr1-1421	Vacuolar aminopeptidase Y, processed to mature form by Prb1p	5,18	54989	0	0	0,00	0	0	0,00	0	0	0,00	0	0	0,00	0	0	0,00	0	0	0,00	2	1	10,12	0	0	0,00
254567287	C4QZ00	PAS_chr1-4_0615	mitochondrial phosphate carrier protein	9,33	32856	0	0	0,00	0	0	0,00	0	0	0,00	0	0	0,00	0	0	0,00	0	0	0,00	2	7	25,18	0	0	0,00
254567323	C4QZ18	RBP1	Protein of unknown function that may interact with ribosomes	4,82	118791	0	0	0,00	0	0	0,00	0	0	0,00	0	0	0,00	0	0	0,00	1	1	10,11	0	0	0,00	0	0	0,00
254567339	C4QZ26	YAP3	transcription factor	4,82	25889	1	1	10,16	0	0	0,00	1	2	10,19	1	1	10,16	2	2	15,16	1	1	10,14	2	1	10,16	0	0	0,00
254567343	C4QZ28	AIP1	Actin-interacting protein	5,69	62264	0	0	0,00	0	0	0,00	2	2	14,16	1	2	10,15	2	2	10,17	0	0	0,00	2	1	10,13	0	0	0,00
254567349	C4QZ31	VPS1	Dynamin-like GTPase required for vacuolar sorting	7,34	76537	0	0	0,00	0	0	0,00	0	0	0,00	0	0	0,00	0	0	0,00	1	1	10,11	0	0	0,00	0	0	0,00
254567355	C4QZ34	OSH6	Oxysterol-binding protein	6,22	49223	2	1	10,18	0	0	0,00	1	1	10,15	0	0	0,00	2	2	15,17	0	0	0,00	2	3	20,21	1	1	10,16
254567379	C4QZ46	PP7435_Chr1-1469	hypothetical protein	5,25	176009	0	0	0,00	0	0	0,00	1	1	10,15	0	0	0,00	0	0	0,00	0	0	0,00	0	0	0,00	0	0	0,00
254567393	C4QZ53	PP7435_Chr1-1476	hypothetical protein	5,09	32159	2	1	10,20	2	1	10,17	1	1	10,18	0	0	0,00	2	1	14,23	0	0	0,00	2	1	10,19	0	0	0,00
254567397	C4QZ55	PP7435_Chr1-1478	hypothetical protein	5,54	122601	0	0	0,00	0	0	0,00	0	0	0,00	0	0	0,00	0	0	0,00	0	0	0,00	0	0	0,00	1	1	10,13
254567405	C4QZ59	PAS_FragB_0002	Protein CSF1	5,40	340940	0	0	0,00	1	1	10,15	0	0	0,00	0	0	0,00	0	0	0,00	0	0	0,00	0	0	0,00	0	0	0,00
254567419	C4QZ66	GCV3	H subunit of the mitochondrial glycine decarboxylase complex	4,19	18808	0	0	0,00	0	0	0,00	0	0	0,00	0	0	0,00	0	0	0,00	1	1	10,14	0	0	0,00	1	1	10,11
254567447	C4QZ80	PP7435_Chr1-1506	hypothetical protein	5,62	37893	0	0	0,00	0	0	0,00	2	1	10,17	0	0	0,00	2	4	25,15	0	0	0,00	2	8	60,21	2	3	20,14
254567485	C4QZ99	PAS_FragB_0040	Serine palmitoyltransferase 1	5,42	61695	0	0	0,00	0	0	0,00	0	0	0,00	0	0	0,00	0	0	0,00	0	0	0,00	1	1	10,17	0	0	0,00
254567487	C4QZA0	PAS_FragB_0041	hypothetical protein	9,71	36458	0	0	0,00	0	0	0,00	0	0	0,00	0	0	0,00	1	1	10,15	0	0	0,00	0	0	0,00	0	0	0,00
254567489	C4QZA1	YAH1	Adrenodoxin homolog, mitochondrial	5,19	17883	0	0	0,00	0	0	0,00	0	0	0,00	0	0	0,00	1	1	10,14	0	0	0,00	0	0	0,00	0	0	0,00
254567515	C4QZB4	SGS1	ATP-dependent helicase	5,82	147766	0	0	0,00	0	0	0,00	0	0	0,00	1	1	10,13	0	0	0,00	0	0	0,00	0	0	0,00	0	0	0,00
254567523	C4QZB8	PCK1	Phosphoenolpyruvate carboxykinase	5,99	62016	2	4	15,24	2	7	15,24	0	0	0,00	0	0	0,00	0	0	0,00	0	0	0,00	0	0	0,00	0	0	0,00
254567545	C4QZC9	PAS_chr2-1_0004	hypothetical protein	6,46	28162	0	0	0,00	0	0	0,00	0	0	0,00	0	0	0,00	1	1	10,11	0	0	0,00	0	0	0,00	0	0	0,00
254567569	C4QZE1	PP7435_Chr2-1289	2-deoxyglucose-6-phosphate phosphatase, similar to Dog2p, member of a family of low molecular weight	4,98	24906	2	1	10,19	2	4	10,19	0	0	0,00	0	0	0,00	0	0	0,00	0	0	0,00	0	0	0,00	0	0	0,00
254567581	C4QZE7	PAS_chr2-1_0022	60S ribosomal protein L7	9,87	27849	2	3	20,17	2	2	15,16	2	2	10,17	2	3	10,17	2	3	10,16	2	2	10,14	2	2	20,14	0	0	0,00
254567583	C4QZE8	PP7435_Chr2-1285	Protein of unknown function that may interact with ribosomes, based on co-purification experiments	4,51	17290	2	1	10,21	2	1	10,19	0	0	0,00	0	0	0,00	2	3	15,20	2	2	10,18	2	11	20,24	2	7	30,20
254567591	C4QZF2	HSF3	Trimeric heat shock transcription factor, activates multiple genes in response to stresses that incl	5,40	59860	1	2	20,15	1	2	10,14	0	0	0,00	0	0	0,00	0	0	0,00	1	1	10,13	0	0	0,00	0	0	0,00
254567623	C4QZG8	FES1	Hsp70 (Ssa1p) nucleotide exchange factor, cytosolic homolog of Sil1p, which is the nucleotide exchan	4,75	32377	0	0	0,00	0	0	0,00	2	4	20,19	1	1	10,17	2	1	10,15	2	1	10,13	0	0	0,00	0	0	0,00
254567658	C4QZI5	PP7435_Chr2-1250	Ubiquitin-conjugating enzyme involved in the error-free DNA postreplication repair pathway	6,37	17355	0	0	0,00	0	0	0,00	0	0	0,00	0	0	0,00	2	2	10,16	0	0	0,00	0	0	0,00	0	0	0,00
254567662	C4QZI7	PP7435_Chr2-1249	Hydroperoxide and superoxide-radical responsive glutathione-dependent oxidoreductase	4,76	26245	0	0	0,00	0	0	0,00	0	0	0,00	0	0	0,00	1	1	10,16	0	0	0,00	0	0	0,00	0	0	0,00
254567720	C4QZL6	sucA	Component of the mitochondrial alpha-ketoglutarate dehydrogenase complex, which catalyzes a key step	6,22	112682	0	0	0,00	0	0	0,00	1	3	30,16	1	1	10,14	2	3	20,21	2	2	15,13	2	17	70,21	2	3	25,16
254567722	C4QZL7	PAS_chr2-1_0090	tRNA methyltransferase, localizes to both the nucleus and mitochondrion to produce the modified base	9,26	57314	0	0	0,00	0	0	0,00	0	0	0,00	0	0	0,00	0	0	0,00	0	0	0,00	1	1	10,13	0	0	0,00
254567728	C4QZM0	PP7435_Chr2-1217	GDP-mannose pyrophosphorylase (mannose-1-phosphate guanyltransferase), synthesizes GDP-mannose from	6,47	49009	0	0	0,00	0	0	0,00	2	2	15,17	1	1	10,14	2	2	10,20	0	0	0,00	2	1	10,23	1	1	10,17
254567736	C4QZM4	PP7435_Chr2-1213	Essential iron-sulfur protein required for ribosome biogenesis and translation initiation	8,94	68080	0	0	0,00	2	1	10,14	0	0	0,00	1	1	10,14	2	2	10,13	1	1	10,14	2	1	10,15	0	0	0,00
254567738	C4QZM5	PAB1	Poly(A) binding protein, part of the 3'-end RNA-processing complex, mediates interactions between th	5,00	70017	1	1	10,14	1	1	10,14	2	3	20,17	0	0	0,00	2	2	20,18	0	0	0,00	2	3	10,20	0	0	0,00
254567754	C4QZN3	BMH1	hypothetical protein	4,65	28989	2	2	10,25	2	1	10,24	0	0	0,00	2	1	10,11	1	1	10,12	0	0	0,00	2	2	10,14	0	0	0,00
254567788	C4QZQ0	IDH2	Subunit of mitochondrial NAD(+)-dependent isocitrate dehydrogenase, which catalyzes the oxidation of	8,05	39963	0	0	0,00	0	0	0,00	1	1	10,13	0	0	0,00	1	1	10,16	2	1	10,14	2	4	30,14	2	2	15,15
254567798	C4QZQ5	EFT1	hypothetical protein	6,27	93392	1	1	10,15	0	0	0,00	2	9	54,20	2	3	25,17	2	3	25,19	2	6	35,18	2	11	62,19	2	3	20,16
254567802	C4QZQ7	PP7435_Chr2-1181	RNA polymerase II second largest subunit B150, part of central core	6,32	139452	0	0	0,00	0	0	0,00	0	0	0,00	0	0	0,00	0	0	0,00	1	1	10,11	0	0	0,00	0	0	0,00
254567834	C4QZS3	KAR2	ATPase involved in protein import into the ER, also acts as a chaperone to mediate protein folding i	4,63	74150	2	5	35,20	2	5	35,21	2	18	65,24	2	6	25,23	2	6	30,22	2	12	48,18	2	7	45,21	2	22	60,21
254567870	C4QZU1	PAS_chr2-1_0159	UBX (ubiquitin regulatory X) domain-containing protein that regulates Glc7p phosphatase activity and	4,66	36660	2	1	10,18	2	1	10,16	1	1	10,17	1	5	20,20	2	3	10,21	0	0	0,00	2	2	10,17	1	1	10,16
254567876	C4QZU4	PP7435_Chr2-1148	Histidine kinase osmosensor that regulates a MAP kinase cascade	5,26	121523	0	0	0,00	0	0	0,00	0	0	0,00	0	0	0,00	1	1	10,16	0	0	0,00	2	2	15,15	0	0	0,00
254567890	C4QZV1	PP7435_Chr2-1141	Protein that is processed in the mitochondrion to yield acetylglutamate kinase and N-acetyl-gamma-gl	7,00	92546	2	3	15,20	2	2	10,15	2	4	30,21	1	2	10,17	2	2	20,22	2	4	25,20	2	1	12,18	2	4	17,20
254567896	C4QZV4	PP7435_Chr2-1138	Putative protein of unknown function	3,86	17444	0	0	0,00	0	0	0,00	0	0	0,00	0	0	0,00	2	1	10,12	0	0	0,00	2	1	10,13	0	0	0,00
254567900	C4QZV6	glgP	Non-essential glycogen phosphorylase required for the mobilization of glycogen, activity is regulate	5,47	97607	2	11	30,23	2	5	30,19	2	2	10,22	0	0	0,00	2	3	24,22	0	0	0,00	2	3	12,22	2	1	10,19
254567908	C4QZW0	TMT1	hypothetical protein	4,86	32022	2	2	10,16	2	3	10,18	1	1	10,14	0	0	0,00	2	1	10,18	0	0	0,00	2	2	10,17	2	1	10,16
254567932	C4QZX2	PRE8	Alpha 2 subunit of the 20S proteasome	5,59	27310	0	0	0,00	0	0	0,00	2	6	10,20	1	2	20,16	2	2	20,14	2	6	20,22	2	12	20,21	2	17	25,23
254567940	C4QZX6	MSC1	Protein of unknown function	9,42	66342	0	0	0,00	0	0	0,00	0	0	0,00	0	0	0,00	0	0	0,00	0	0	0,00	2	4	15,23	2	2	10,24
254567996	C4R004	PP7435_Chr2-1087	hypothetical protein	4,76	14633	0	0	0,00	1	2	20,15	0	0	0,00	0	0	0,00	2	1	10,17	0	0	0,00	0	0	0,00	2	1	10,22
254568036	C4R024	MDH1	Mitochondrial malate dehydrogenase, catalyzes interconversion of malate and oxaloacetate	6,37	34868	2	2	10,13	2	2	20,14	0	0	0,00	0	0	0,00	2	1	10,15	0	0	0,00	2	8	60,19	2	2	15,18
254568040	C4R026	PP7435_Chr2-1066	Integral membrane protein localized to late Golgi vesicles along with the v-SNARE Tlg2p	6,09	18417	0	0	0,00	0	0	0,00	1	1	10,11	0	0	0,00	0	0	0,00	0	0	0,00	0	0	0,00	0	0	0,00
254568056	C4R034	VMA5	Subunit C of the eight-subunit V1 peripheral membrane domain of vacuolar H+-ATPase (V-ATPase)	5,24	44103	0	0	0,00	0	0	0,00	0	0	0,00	0	0	0,00	0	0	0,00	0	0	0,00	2	4	20,26	0	0	0,00
254568072	C4R042	PP7435_Chr2-1051	hypothetical protein	5,84	50166	0	0	0,00	0	0	0,00	0	0	0,00	0	0	0,00	0	0	0,00	0	0	0,00	1	1	10,12	0	0	0,00
254568098	C4R055	PAS_chr2-1_0267	Conserved 90S pre-ribosomal component essential for proper endonucleolytic cleavage of the 35 S rRNA	5,26	98783	0	0	0,00	0	0	0,00	0	0	0,00	0	0	0,00	0	0	0,00	0	0	0,00	1	1	10,14	0	0	0,00
254568110	C4R061	PAS_chr2-1_0271	Subunit of the Hat1p-Hat2p histone acetyltransferase complex	5,05	44449	0	0	0,00	0	0	0,00	0	0	0,00	0	0	0,00	0	0	0,00	0	0	0,00	1	1	10,14	0	0	0,00
254568122	C4R067	TOA1	TFIIA large subunit	4,07	27521	0	0	0,00	0	0	0,00	1	1	10,12	0	0	0,00	1	1	10,13	0	0	0,00	0	0	0,00	0	0	0,00
254568132	C4R072	ZUO1	Cytosolic ribosome-associated chaperone	7,70	50821	1	1	10,19	1	1	10,18	2	9	40,22	2	4	20,22	2	4	30,19	2	3	10,23	2	1	10,20	1	1	10,21
254568162	C4R087	PP7435_Chr2-1007	Essential 113kDa subunit of the exocyst complex	5,48	93800	0	0	0,00	0	0	0,00	1	1	10,13	0	0	0,00	0	0	0,00	0	0	0,00	0	0	0,00	0	0	0,00
254568170	C4R091	PP7435_Chr2-1001	Voltage-gated high-affinity calcium channel	6,72	239957	0	0	0,00	0	0	0,00	2	3	10,24	0	0	0,00	2	3	10,22	0	0	0,00	2	2	10,19	0	0	0,00
254568174	C4R093	PP7435_Chr2-0999	hypothetical protein	4,88	89683	0	0	0,00	0	0	0,00	0	0	0,00	1	1	10,12	0	0	0,00	0	0	0,00	0	0	0,00	0	0	0,00
254568184	C4R098	AOC3	hypothetical protein	4,25	89983	0	0	0,00	0	0	0,00	0	0	0,00	0	0	0,00	0	0	0,00	0	0	0,00	2	1	10,15	0	0	0,00
254568186	C4R099	ZWF1	Glucose-6-phosphate dehydrogenase (G6PD), catalyzes the first step of the pentose phosphate pathway	6,12	57635	2	7	20,24	2	5	20,23	2	9	35,24	1	3	30,18	2	8	25,23	2	4	25,17	2	15	80,22	2	6	25,20
254568192	C4R0A2	PP7435_Chr2-0991	NAD(+)-dependent glutamate dehydrogenase, degrades glutamate to ammonia and alpha-ketoglutarate	5,90	117609	1	1	10,16	1	1	10,14	0	0	0,00	0	0	0,00	0	0	0,00	0	0	0,00	2	2	15,20	0	0	0,00
254568196	C4R0A4	PP7435_Chr2-0990	Bifunctional enzyme with alcohol dehydrogenase and glutathione-dependent formaldehyde dehydrogenase	5,71	38581	2	2	20,18	2	2	15,18	0	0	0,00	0	0	0,00	2	2	15,15	0	0	0,00	2	2	15,17	1	2	20,19
254568216	C4R0B4	CAL1	Calnexin	4,35	63027	2	2	10,21	2	4	15,23	2	2	15,19	2	2	15,13	2	2	15,23	1	1	10,20	2	10	40,25	2	13	45,25
254568220	C4R0B6	PP7435_Chr2-0977	hypothetical protein	4,65	56683	0	0	0,00	0	0	0,00	0	0	0,00	0	0	0,00	0	0	0,00	0	0	0,00	1	1	10,21	0	0	0,00
254568222	C4R0B7	clpB	Oligomeric mitochondrial matrix chaperone	5,84	92768	0	0	0,00	0	0	0,00	0	0	0,00	0	0	0,00	0	0	0,00	0	0	0,00	1	2	10,16	2	1	10,16
254568230	C4R0C1	PP7435_Chr2-0972	Palmitoyl transferase involved in protein palmitoylation	8,82	68296	0	0	0,00	0	0	0,00	0	0	0,00	0	0	0,00	0	0	0,00	1	1	10,12	0	0	0,00	0	0	0,00
254568236	C4R0C4	PP7435_Chr2-0969	Transcriptional activator of proline utilization genes	5,74	100251	0	0	0,00	0	0	0,00	0	0	0,00	0	0	0,00	1	1	10,15	0	0	0,00	0	0	0,00	0	0	0,00
254568270	C4R0E1	PP7435_Chr2-0954	hypothetical protein	6,62	16185	0	0	0,00	0	0	0,00	0	0	0,00	0	0	0,00	0	0	0,00	0	0	0,00	1	1	10,12	0	0	0,00
254568300	C4R0F6	PP7435_Chr2-0940	Putative protein with sequence similarity to hydroxyacid dehydrogenases	6,35	40236	0	0	0,00	0	0	0,00	2	2	10,17	1	1	10,16	1	1	10,14	0	0	0,00	0	0	0,00	0	0	0,00
254568302	C4R0F7	PAS_chr2-1_0361	Subunit IV of cytochrome c oxidase	7,82	18041	0	0	0,00	0	0	0,00	0	0	0,00	0	0	0,00	0	0	0,00	0	0	0,00	2	1	10,16	0	0	0,00
254568306	C4R0F9	PP7435_Chr2-0938	hypothetical protein	5,02	52480	0	0	0,00	0	0	0,00	0	0	0,00	0	0	0,00	0	0	0,00	0	0	0,00	1	1	10,11	0	0	0,00
254568308	C4R0G0	PP7435_Chr2-0937	Subunit VIa of cytochrome c oxidase, which is the terminal member of the mitochondrial inner membran	9,78	13989	0	0	0,00	2	1	10,12	0	0	0,00	0	0	0,00	0	0	0,00	0	0	0,00	2	1	10,13	0	0	0,00
254568334	C4R0H3	PAS_chr2-1_0376	Putative GTPase, member of the Obg family	4,09	23252	2	11	29,21	2	13	30,20	2	23	45,23	2	11	35,22	2	25	65,26	2	20	45,23	2	11	40,21	2	12	35,21
254568386	Q92448	PFK1	Alpha subunit of heterooctameric phosphofructokinase involved in glycolysis	5,60	108731	0	0	0,00	0	0	0,00	0	0	0,00	0	0	0,00	0	0	0,00	0	0	0,00	0	0	0,00	1	1	10,14
254568396	C4R0K4	PP7435_Chr2-0893	Serine/threonine protein phosphatase Z, isoform of Ppz2p	8,52	60723	0	0	0,00	0	0	0,00	0	0	0,00	0	0	0,00	0	0	0,00	0	0	0,00	1	1	10,14	0	0	0,00
254568406	C4R0K9	YIM1	Protein of unknown function	9,07	40742	0	0	0,00	0	0	0,00	0	0	0,00	0	0	0,00	0	0	0,00	0	0	0,00	2	1	10,13	0	0	0,00
254568412	C4R0L2	PP7435_Chr2-0884	Alpha-isopropylmalate synthase (2-isopropylmalate synthase)	5,53	63589	0	0	0,00	0	0	0,00	2	1	10,15	0	0	0,00	0	0	0,00	0	0	0,00	1	1	10,14	1	1	10,15
254568416	C4R0L4	BUB1	Protein kinase that forms a complex with Mad1p and Bub3p	5,86	80790	0	0	0,00	0	0	0,00	0	0	0,00	0	0	0,00	0	0	0,00	0	0	0,00	1	1	10,13	0	0	0,00
254568418	C4R0L5	PP7435_Chr2-0881	Transmembrane protein subunit of the glycosylphosphatidylinositol transamidase complex	5,38	69254	0	0	0,00	0	0	0,00	0	0	0,00	0	0	0,00	0	0	0,00	0	0	0,00	1	1	10,15	0	0	0,00
254568432	C4R0M2	WBP1	Beta subunit of the oligosaccharyl transferase (OST) glycoprotein complex	4,40	48847	0	0	0,00	0	0	0,00	0	0	0,00	0	0	0,00	0	0	0,00	0	0	0,00	2	1	10,15	1	1	10,15
254568464	C4R0N8	PAS_chr2-1_0892	hypothetical protein	4,47	40538	2	2	15,13	2	2	10,13	1	1	10,13	0	0	0,00	2	1	10,14	1	1	10,13	0	0	0,00	0	0	0,00
254568470	C4R0P1	TDH1	Glyceraldehyde-3-phosphate dehydrogenase, isozyme 3, involved in glycolysis and gluconeogenesis	6,27	35563	1	1	10,14	0	0	0,00	1	2	10,22	0	0	0,00	2	3	10,23	2	2	10,15	2	4	35,16	2	2	15,17
254568478	C4R0P5	PP7435_Chr2-0854	Mitochondrial ribosomal protein of the large subunit	5,53	33995	2	1	10,13	2	1	10,13	0	0	0,00	0	0	0,00	0	0	0,00	0	0	0,00	0	0	0,00	0	0	0,00
254568492	C4R0Q2	GSP1	GTP binding protein (mammalian Ranp homolog)	6,61	24067	0	0	0,00	0	0	0,00	2	1	10,17	0	0	0,00	2	2	20,25	0	0	0,00	2	9	40,34	2	3	30,22
254568496	C4R0Q4	PAS_chr2-1_0451	hypothetical protein	4,62	13301	2	7	50,25	2	5	35,27	2	5	30,24	1	2	10,17	2	7	35,24	0	0	0,00	2	13	50,32	2	13	50,31
254568506	C4R0Q9	PP7435_Chr2-0840	hypothetical protein	6,07	79479	0	0	0,00	0	0	0,00	0	0	0,00	0	0	0,00	0	0	0,00	0	0	0,00	1	1	10,17	0	0	0,00
254568544	C4R0S8	ADH	Mitochondrial alcohol dehydrogenase isozyme III	5,81	36958	2	74	140,17	2	136	174,19	2	2	15,15	1	1	10,15	2	3	25,15	2	14	50,19	2	33	65,18	2	13	45,18
254568562	C4R0T7	PP7435_Chr2-0813	40S ribosomal protein S14	11,38	15181	2	2	10,13	2	2	10,14	1	1	10,18	2	1	10,15	1	1	10,15	2	1	10,15	2	1	10,17	0	0	0,00
254568572	C4R0U2	PP7435_Chr2-0809	ubiquitin-60S ribosomal protein L40 fusion protein	10,27	14541	0	0	0,00	0	0	0,00	0	0	0,00	0	0	0,00	0	0	0,00	0	0	0,00	1	1	10,12	1	1	10,11
254568576	C4R0U4	PAS_chr2-1_0488	hypothetical protein	5,10	67838	0	0	0,00	1	1	10,13	0	0	0,00	0	0	0,00	0	0	0,00	0	0	0,00	1	1	10,17	2	2	15,16
254568582	C4R0U7	PP7435_Chr2-0804	Nitrogen catabolite repression transcriptional regulator	5,59	26759	0	0	0,00	0	0	0,00	0	0	0,00	0	0	0,00	2	2	15,16	0	0	0,00	2	4	15,23	1	1	10,16
254568588	C4R0V0	PP7435_Chr2-0801	Essential NTPase required for small ribosome subunit synthesis	4,12	21371	0	0	0,00	0	0	0,00	0	0	0,00	0	0	0,00	0	0	0,00	0	0	0,00	1	1	10,20	0	0	0,00
254568594	C4R0V3	PAS_chr2-1_0497	Single-domain racemase, possibly non-specific due to the lack of the second domain	8,80	29853	0	0	0,00	0	0	0,00	0	0	0,00	0	0	0,00	0	0	0,00	0	0	0,00	1	2	10,17	0	0	0,00
254568606	C4R0V9	PAS_chr2-1_0502	Thiol-specific peroxiredoxin, reduces hydroperoxides to protect against oxidative damage	9,06	19180	0	0	0,00	0	0	0,00	0	0	0,00	0	0	0,00	0	0	0,00	2	1	10,14	0	0	0,00	0	0	0,00
254568616	C4R0W4	PP7435_Chr2-0787	hypothetical protein	5,91	56736	1	1	10,12	1	1	10,11	2	3	15,14	0	0	0,00	2	3	20,15	2	3	15,13	2	18	80,22	2	12	50,22
254568642	C4R0X7	STI1	Hsp90 cochaperone, interacts with the Ssa group of the cytosolic Hsp70 chaperones	5,03	63435	0	0	0,00	0	0	0,00	2	3	25,17	1	2	10,16	2	1	10,16	2	1	10,14	2	2	15,16	2	2	15,15
254568646	C4R0X9	SNF1	hypothetical protein	5,90	62669	1	1	10,15	1	1	10,13	2	1	10,14	0	0	0,00	2	1	10,13	0	0	0,00	2	4	15,18	0	0	0,00
254568654	C4R0Y3	TCT1	Protein that associates with ribosomes	4,24	18519	0	0	0,00	0	0	0,00	2	5	10,16	1	2	10,12	2	2	10,15	2	3	10,15	2	3	15,15	2	4	20,18
254568680	C4R0Z6		Gamma-adaptin, large subunit of the clathrin-associated protein (AP-1) complex	6,52	89140	0	0	0,00	0	0	0,00	0	0	0,00	0	0	0,00	1	1	10,18	0	0	0,00	2	2	10,20	0	0	0,00
254568690	C4R101	PP7435_Chr2-0749	Putative benzil reductase	5,63	27247	0	0	0,00	0	0	0,00	0	0	0,00	0	0	0,00	0	0	0,00	0	0	0,00	2	1	10,23	1	1	10,18
254568718	C4R115	Cct5	Subunit of the cytosolic chaperonin Cct ring complex, related to Tcp1p	4,99	60694	0	0	0,00	0	0	0,00	0	0	0,00	0	0	0,00	0	0	0,00	1	1	10,14	1	1	10,20	0	0	0,00
254568720	C4R116	ARP3	Essential component of the Arp2/3 complex	5,48	47703	0	0	0,00	0	0	0,00	0	0	0,00	0	0	0,00	0	0	0,00	0	0	0,00	2	3	20,19	0	0	0,00
254568722	C4R117	PP7435_Chr2-0732	PIK-related protein kinase and rapamycin target	6,46	275747	0	0	0,00	0	0	0,00	1	1	10,17	0	0	0,00	0	0	0,00	0	0	0,00	0	0	0,00	0	0	0,00
254568730	C4R121	PP7435_Chr2-0728	hypothetical protein	4,04	59355	0	0	0,00	0	0	0,00	1	1	10,15	0	0	0,00	0	0	0,00	0	0	0,00	0	0	0,00	0	0	0,00
254568732	C4R122	CYK1	hypothetical protein	7,91	40375	2	6	20,20	2	5	20,18	2	3	10,20	2	2	10,17	2	6	15,21	2	4	10,19	1	1	10,18	2	5	30,22

254568758	C4R135	PP7435_Chr2-0714	Putative xylose and arabinose reductase	6,09	32089	0	0	0,00	0	0	0,00	0	0	0,00	0	0	0,00	0	0	0,00	0	0	0,00	1	1	10,13	0	0	0,00
254568770	C4R141	ILV6	hypothetical protein	6,10	32619	1	1	10,13	2	1	10,13	2	3	10,14	0	0	0,00	2	2	10,15	0	0	0,00	2	4	25,19	2	2	10,13
254568772	C4R142	PAS_chr2-1_0580	Cytosolic NADP-specific isocitrate dehydrogenase	5,47	48706	2	1	10,19	2	3	10,19	0	0	0,00	0	0	0,00	1	1	10,17	0	0	0,00	2	3	10,19	1	2	10,14
254568776	C4R144	PP7435_Chr2-0705	hypothetical protein	6,56	163068	0	0	0,00	0	0	0,00	1	1	10,12	0	0	0,00	0	0	0,00	0	0	0,00	0	0	0,00	0	0	0,00
254568788	C4R150	PP7435_Chr2-0699	Subunit (61/68 kDa) of TFIID and SAGA complexes	10,25	66563	0	0	0,00	1	1	10,12	0	0	0,00	0	0	0,00	0	0	0,00	0	0	0,00	0	0	0,00	0	0	0,00
254568792	C4R152	PP7435_Chr2-0697	Subunit of signal peptidase complex (Spc1p, Spc2p, Spc3p, Sec11p)	7,52	19945	0	0	0,00	0	0	0,00	0	0	0,00	0	0	0,00	0	0	0,00	1	1	10,16	0	0	0,00	0	0	0,00
254568796	C4R154	CRN1	Coronin, cortical actin cytoskeletal component	5,10	71638	0	0	0,00	1	1	10,17	0	0	0,00	0	0	0,00	0	0	0,00	0	0	0,00	0	0	0,00	0	0	0,00
254568800	C4R156	PAS_chr2-1_0593	Dihydroorotase, catalyzes the third enzymatic step in the de novo biosynthesis of pyrimidines	6,20	40043	2	3	10,19	0	0	0,00	0	0	0,00	0	0	0,00	0	0	0,00	0	0	0,00	0	0	0,00	0	0	0,00
254568814	C4R163	PP7435_Chr2-0685	Conserved nuclear regulatory subunit of Glc7p type 1 protein serine-threonine phosphatase (PP1)	4,74	41013	0	0	0,00	0	0	0,00	0	0	0,00	0	0	0,00	1	1	10,15	0	0	0,00	2	1	10,16	2	1	10,15
254568824	C4R168	PP7435_Chr2-0679	Putative Nedd8 ligase	4,40	30507	0	0	0,00	0	0	0,00	0	0	0,00	0	0	0,00	0	0	0,00	0	0	0,00	1	1	10,13	0	0	0,00
254568830	C4R171	SNF4	Activating gamma subunit of the AMP-activated Snf1p kinase complex	5,19	36591	0	0	0,00	1	1	10,14	0	0	0,00	0	0	0,00	2	1	10,14	0	0	0,00	1	1	10,16	0	0	0,00
254568836	C4R174	PP7435_Chr2-0673	hypothetical protein	5,96	41011	0	0	0,00	0	0	0,00	0	0	0,00	0	0	0,00	1	1	10,19	0	0	0,00	2	6	24,21	2	5	34,21
254568840	C4R176	POL5	hypothetical protein	5,15	109298	0	0	0,00	0	0	0,00	1	1	10,15	0	0	0,00	1	1	10,13	0	0	0,00	0	0	0,00	0	0	0,00
254568852	C4R182	PP7435_Chr2-0667	Cytoplasmic protein of unknown function	4,82	72974	0	0	0,00	0	0	0,00	0	0	0,00	0	0	0,00	0	0	0,00	0	0	0,00	1	1	10,13	0	0	0,00
254568868	C4R190	AAD5	6-phosphofructo-2-kinase	6,29	39102	0	0	0,00	0	0	0,00	0	0	0,00	0	0	0,00	0	0	0,00	0	0	0,00	1	1	10,13	0	0	0,00
254568876	C4R194	GFA1	Glutamine-fructose-6-phosphate amidotransferase	5,91	77304	0	0	0,00	0	0	0,00	1	1	10,12	0	0	0,00	0	0	0,00	1	2	20,15	2	1	10,14	1	1	10,13
254568888	C4R1A0	SAC6	Fimbrin, actin-bundling protein	5,12	70161	0	0	0,00	0	0	0,00	2	2	10,14	0	0	0,00	2	2	10,17	1	1	10,16	2	14	65,20	2	4	15,17
254568894	C4R1A3	PP7435_Chr2-0646	40S ribosomal protein S27	8,53	8919	0	0	0,00	0	0	0,00	1	1	10,17	1	1	10,13	2	2	10,16	0	0	0,00	0	0	0,00	0	0	0,00
254568896	C4R1A4	GLC7	Catalytic subunit of type 1 serine/threonine protein phosphatase	5,18	36827	1	1	10,12	1	1	10,11	2	1	10,12	1	1	10,11	2	3	10,14	1	2	20,12	2	2	20,13	1	1	10,12
254568902	C4R1A7	ARO2	Bifunctional chorismate synthase and flavin reductase	6,40	40486	2	1	10,18	1	2	10,19	1	1	10,17	0	0	0,00	1	1	10,20	0	0	0,00	1	2	10,19	1	1	10,20
254568908	C4R1B0	PP7435_Chr2-0640	Putative protein of unknown function	6,35	21714	0	0	0,00	0	0	0,00	0	0	0,00	0	0	0,00	0	0	0,00	0	0	0,00	2	7	30,18	1	1	10,15
254568914	C4R1B3	PP7435_Chr2-0637	Protein implicated in Mms22-dependent DNA repair during S phase	7,76	107653	0	0	0,00	0	0	0,00	1	1	10,16	0	0	0,00	0	0	0,00	0	0	0,00	0	0	0,00	0	0	0,00
254568924	C4R1B8	GAR1	Protein component of the H/ACA snoRNP pseudouridylase complex	11,56	20758	2	1	10,14	2	1	10,13	2	2	10,14	0	0	0,00	1	2	10,13	1	1	10,12	1	1	10,13	0	0	0,00
254568934	C4R1C3	PP7435_Chr2-0629	Arginine/alanine aminopeptidase, overproduction stimulates glycogen accumulation	5,00	99698	0	0	0,00	0	0	0,00	0	0	0,00	1	1	10,15	0	0	0,00	0	0	0,00	2	2	10,12	0	0	0,00
254568940	C4R1C6	PP7435_Chr2-0626	Nucleolar protein, component of the small subunit (SSU) processome	7,55	58246	0	0	0,00	0	0	0,00	0	0	0,00	0	0	0,00	0	0	0,00	0	0	0,00	0	0	0,00	1	1	10,13
254568944	C4R1C8	SER3	3-phosphoglycerate dehydrogenase, catalyzes the first step in serine and glycine biosynthesis	5,79	50974	0	0	0,00	0	0	0,00	0	0	0,00	0	0	0,00	1	1	10,14	0	0	0,00	2	3	20,16	1	2	10,15
254568946	C4R1C9	PP7435_Chr2-0623	60S ribosomal protein L15	11,80	24216	2	2	20,14	0	0	0,00	1	1	10,14	0	0	0,00	0	0	0,00	0	0	0,00	2	10	20,17	0	0	0,00
254568950	C4R1D1	PAS_chr2-1_0660	Enzyme of 'de novo' purine biosynthesis	6,76	63248	2	2	15,14	2	3	15,16	2	2	15,17	2	2	10,17	0	0	0,00	0	0	0,00	2	4	20,18	2	2	20,17
254568952	C4R1D2	GSL2	Catalytic subunit of 1,3-beta-D-glucan synthase	7,32	215060	0	0	0,00	0	0	0,00	0	0	0,00	0	0	0,00	0	0	0,00	0	0	0,00	1	1	10,16	1	1	10,18
254568972	C4R1E2	PAS_chr2-1_0670	Essential protein of the mitochondrial intermembrane space (IMS)	4,35	26687	0	0	0,00	0	0	0,00	1	1	10,15	0	0	0,00	0	0	0,00	0	0	0,00	0	0	0,00	2	3	20,21
254568982	C4R1E7	PAS_chr2-1_0675	Mitochondrial inner membrane protein with similarity to Mdm32p	8,94	68165	0	0	0,00	1	1	10,10	0	0	0,00	0	0	0,00	0	0	0,00	0	0	0,00	0	0	0,00	0	0	0,00
254569010	C4R1G1	PP7435_Chr2-0592	Essential protein involved in transcription regulation	4,99	51207	0	0	0,00	0	0	0,00	2	1	10,15	0	0	0,00	2	1	10,13	0	0	0,00	2	2	10,19	2	2	10,16
254569032	C4R1H2	PP7435_Chr2-0581	Putative protein of unknown function with similarity to human thiosulfate sulfurtransferase	6,26	34336	0	0	0,00	0	0	0,00	0	0	0,00	0	0	0,00	0	0	0,00	0	0	0,00	2	2	10,16	0	0	0,00
254569058	C4R1I5	AFG2	ATPase of the CDC48/PAS1/SEC18 (AAA) family, forms a hexameric complex	5,08	83485	0	0	0,00	0	0	0,00	1	1	10,12	0	0	0,00	0	0	0,00	0	0	0,00	0	0	0,00	0	0	0,00
254569066	C4R1I9	PP7435_Chr2-0564	SUMO-conjugating enzyme involved in the Smt3p conjugation pathway	9,44	17942	2	1	10,17	2	1	10,13	2	2	15,16	0	0	0,00	2	1	10,15	0	0	0,00	0	0	0,00	0	0	0,00
254569068	C4R1J0	PEX19	PEX19	3,93	33688	0	0	0,00	0	0	0,00	2	1	10,28	0	0	0,00	1	1	10,33	2	2	10,23	0	0	0,00	2	1	10,33
254569082	C4R1J7	PP7435_Chr2-0556	Protein of unknown function with similarity to Ykl050cp and Uso1p	5,79	91233	0	0	0,00	0	0	0,00	0	0	0,00	0	0	0,00	0	0	0,00	0	0	0,00	0	0	0,00	1	1	10,18
254569090	C4R1K1	CUE3	Protein of unknown function	9,46	73709	0	0	0,00	0	0	0,00	0	0	0,00	1	1	10,15	0	0	0,00	0	0	0,00	0	0	0,00	0	0	0,00
254569094	C4R1K3	PP7435_Chr2-0549	60S ribosomal protein L28	11,50	16441	1	1	10,16	0	0	0,00	0	0	0,00	0	0	0,00	1	1	10,16	0	0	0,00	1	1	10,14	0	0	0,00
254569100	C4R1K6	erf1	Endoribonuclease	5,72	42367	0	0	0,00	0	0	0,00	0	0	0,00	0	0	0,00	0	0	0,00	0	0	0,00	1	1	10,20	0	0	0,00
254569102	C4R1K7	PP7435_Chr2-0545	Protein that binds tRNA and methionyl-and glutamyl-tRNA synthetases (Mes1p and Gus1p)	8,89	40381	0	0	0,00	0	0	0,00	2	1	10,13	1	1	10,11	0	0	0,00	0	0	0,00	0	0	0,00	0	0	0,00
254569118	C4R1L5	TPS3	Regulatory subunit of trehalose-6-phosphate synthase/phosphatase complex	5,61	116405	0	0	0,00	0	0	0,00	0	0	0,00	0	0	0,00	0	0	0,00	0	0	0,00	2	1	14,16	0	0	0,00
254569140	C4R1M6	DAL2	Allantoicase, converts allantoate to urea and ureidoglycolate	5,96	37847	0	0	0,00	0	0	0,00	1	1	10,17	0	0	0,00	2	1	10,17	0	0	0,00	2	5	20,22	2	2	15,19
254569156	C4R1N4	EGD2	Alpha subunit of the heteromeric nascent polypeptide-associated complex (NAC)	4,42	21646	0	0	0,00	1	1	10,11	0	0	0,00	0	0	0,00	0	0	0,00	0	0	0,00	2	1	10,18	0	0	0,00
254569162	C4R1N7	CMD1	Calmodulin	4,15	16850	2	2	15,16	2	5	35,16	2	8	45,19	1	2	20,14	2	4	15,15	2	6	20,21	2	4	20,21	2	4	35,16
254569168	C4R1P0	PP7435_Chr2-0512	Spermidine synthase, involved in biosynthesis of spermidine and also in biosynthesis of pantothenic	5,18	26738	0	0	0,00	0	0	0,00	0	0	0,00	0	0	0,00	0	0	0,00	0	0	0,00	2	1	10,12	2	1	10,15
254569182	C4R1P7	acsB	Acetyl-coA synthetase isoform	5,70	73855	0	0	0,00	0	0	0,00	0	0	0,00	0	0	0,00	1	1	10,13	0	0	0,00	1	1	10,15	0	0	0,00
254569186	C4R1P9	PYK2	Pyruvate kinase	6,09	55573	2	14	35,22	2	16	40,22	2	6	40,23	2	2	15,20	2	5	35,20	2	1	10,21	2	10	50,24	2	14	55,24
254569208	C4R1R0	ROT2	Glucosidase II catalytic subunit required for normal cell wall synthesis	5,41	103665	0	0	0,00	0	0	0,00	0	0	0,00	0	0	0,00	0	0	0,00	0	0	0,00	2	2	15,14	1	1	10,13
254569212	C4R1R2	PP7435_Chr2-0491	40S ribosomal protein S17	10,73	15662	0	0	0,00	0	0	0,00	1	1	10,13	0	0	0,00	0	0	0,00	0	0	0,00	0	0	0,00	0	0	0,00
254569230	C4R1S1	fet3	Ferro-O2-oxidoreductase	4,06	70848	0	0	0,00	0	0	0,00	0	0	0,00	0	0	0,00	0	0	0,00	1	1	10,11	0	0	0,00	0	0	0,00
254569254	C4R1T3	PP7435_Chr2-0472	Putative protein of unknown function	5,60	39984	0	0	0,00	1	1	10,15	0	0	0,00	0	0	0,00	0	0	0,00	0	0	0,00	0	0	0,00	0	0	0,00
254569268	C4R1U0	PP7435_Chr2-0465	Protein with ubiquitin-like N terminus, recognizes and binds damaged DNA (with Rad4p)	4,28	37143	0	0	0,00	0	0	0,00	1	1	10,12	0	0	0,00	2	2	10,16	1	1	10,11	2	1	10,15	0	0	0,00
254569284	C4R1U8	agl	Glycogen debranching enzyme containing glucanotranferase and alpha-1,6-amyloglucosidase activities	5,56	173742	0	0	0,00	0	0	0,00	0	0	0,00	0	0	0,00	0	0	0,00	0	0	0,00	1	1	10,13	0	0	0,00
254569294	C4R1V3	PP7435_Chr2-0453	Regulatory subunit of the Atg1p signaling complex	9,36	67680	0	0	0,00	0	0	0,00	0	0	0,00	0	0	0,00	0	0	0,00	0	0	0,00	1	1	10,15	0	0	0,00
254569298	C4R1V5	QCR2	Subunit 2 of the ubiquinol cytochrome-c reductase complex	9,05	37377	2	2	10,13	2	2	10,13	0	0	0,00	0	0	0,00	0	0	0,00	1	1	10,13	0	0	0,00	0	0	0,00
254569318	C4R1W5	PP7435_Chr2-0442	Vacuolar membrane protein involved in vacuolar polyphosphate accumulation	8,23	81253	0	0	0,00	0	0	0,00	0	0	0,00	0	0	0,00	0	0	0,00	0	0	0,00	1	1	10,18	0	0	0,00
254569320	C4R1W6	PP7435_Chr2-0441	Putative protein of unknown function	4,87	58247	0	0	0,00	0	0	0,00	1	1	10,12	1	1	10,10	0	0	0,00	1	1	10,14	0	0	0,00	0	0	0,00
254569328	C4R1X0	PP7435_Chr2-0436	Protein with a role in maintenance of cellular integrity	7,00	133451	0	0	0,00	0	0	0,00	0	0	0,00	0	0	0,00	0	0	0,00	0	0	0,00	2	1	10,15	0	0	0,00
254569338	C4R1X5	PUT2	hypothetical protein	6,40	64803	2	3	15,16	1	3	10,16	0	0	0,00	0	0	0,00	0	0	0,00	0	0	0,00	2	2	10,17	0	0	0,00
254569340	C4R1X6	MEF1	Mitochondrial elongation factor involved in translational elongation	5,84	83059	0	0	0,00	0	0	0,00	0	0	0,00	0	0	0,00	0	0	0,00	0	0	0,00	1	1	10,21	1	1	10,13
254569358	C4R1Y5	JIP5	WD repeat-containing protein	5,17	51991	0	0	0,00	0	0	0,00	0	0	0,00	0	0	0,00	0	0	0,00	0	0	0,00	1	1	10,17	0	0	0,00
254569368	C4R1Z0	PP7435_Chr2-0413	hypothetical protein	5,67	35852	0	0	0,00	0	0	0,00	0	0	0,00	0	0	0,00	0	0	0,00	0	0	0,00	0	0	0,00	1	1	10,14
254569372	C4R1Z2	POR1	Mitochondrial porin (voltage-dependent anion channel), outer membrane protein	9,43	29584	0	0	0,00	0	0	0,00	0	0	0,00	0	0	0,00	0	0	0,00	0	0	0,00	2	4	20,18	1	1	10,14
254569386	C4R1Z9	PP7435_Chr2-0404	Thiol peroxidase that functions as a hydroperoxide receptor	6,18	18203	2	2	10,21	2	4	15,22	2	13	25,22	2	5	40,20	2	10	40,22	2	3	30,18	2	13	20,24	2	10	20,21
254569388	C4R200	ESS1	Peptidylprolyl-cis/trans-isomerase (PPIase)	8,19	18949	0	0	0,00	0	0	0,00	0	0	0,00	0	0	0,00	0	0	0,00	0	0	0,00	2	1	10,17	0	0	0,00
254569390	C4R201	PAS_chr2-2_0380	Zinc-dependent metallopeptidase yscII, may have a role in obtaining leucine from dipeptide substrate	6,19	104283	0	0	0,00	0	0	0,00	1	1	10,12	0	0	0,00	1	1	10,11	1	1	10,12	0	0	0,00	0	0	0,00
254569392	C4R202	NOP2	Probable RNA m(5)C methyltransferase	5,22	68193	0	0	0,00	0	0	0,00	1	1	10,18	0	0	0,00	0	0	0,00	1	1	10,13	0	0	0,00	0	0	0,00
254569396	C4R204	PP7435_Chr2-0399	Mitochondrial ribosomal component of the small subunit	10,12	54791	0	0	0,00	0	0	0,00	0	0	0,00	1	1	10,16	1	1	10,13	2	1	10,14	0	0	0,00	0	0	0,00
254569400	C4R206	PAS_chr2-2_0375	Peptidyl-prolyl cis-trans isomerase (PPIase), binds to the drugs FK506 and rapamycin	9,63	15092	0	0	0,00	0	0	0,00	1	1	10,11	1	1	10,11	2	3	20,14	0	0	0,00	0	0	0,00	0	0	0,00
254569410	C4R211	PP7435_Chr2-0391	Component (70 kDa) of the TOM (translocase of outer membrane) complex	5,20	67393	1	1	10,17	1	1	10,18	1	1	10,14	0	0	0,00	2	3	10,22	0	0	0,00	2	10	19,28	1	1	10,16
254569416	C4R214	PDZ1	Protein of unknown function which may contribute to lipid homeostasis and/or apoptosis	5,31	108957	0	0	0,00	0	0	0,00	1	1	10,11	0	0	0,00	1	1	10,12	0	0	0,00	2	1	10,16	2	2	10,15
254569478	C4R245	TAL1	Transaldolase, enzyme in the non-oxidative pentose phosphate pathway	4,93	35478	0	0	0,00	0	0	0,00	1	1	10,15	0	0	0,00	0	0	0,00	2	3	29,16	2	8	35,20	1	2	10,16
254569484	C4R248	ETFA	Protein with similarity to mammalian electron transfer flavoprotein complex subunit ETF-alpha	5,09	34408	1	1	10,13	0	0	0,00	1	1	10,15	0	0	0,00	1	1	18,15	0	0	0,00	2	6	25,23	2	5	15,25
254569494	C4R253	ADSL	Adenylosuccinate lyase, catalyzes two steps in the 'de novo' purine nucleotide biosynthetic pathway	5,64	54255	2	5	21,16	2	4	20,15	0	0	0,00	0	0	0,00	0	0	0,00	0	0	0,00	0	0	0,00	0	0	0,00
254569496	C4R254	PP7435_Chr2-0348	Subunit of the ARP2/3 complex	7,30	39811	0	0	0,00	0	0	0,00	0	0	0,00	0	0	0,00	0	0	0,00	0	0	0,00	2	2	10,20	0	0	0,00
254569506	C4R259	MDJ1	Co-chaperone that stimulates the ATPase activity of the HSP70 protein Ssc1p	9,44	53055	0	0	0,00	0	0	0,00	0	0	0,00	0	0	0,00	0	0	0,00	1	1	10,15	2	5	30,20	0	0	0,00
254569512	C4R262	ABP1	Actin-binding protein of the cortical actin cytoskeleton	4,44	60493	1	1	10,12	0	0	0,00	1	1	10,13	0	0	0,00	0	0	0,00	0	0	0,00	1	2	20,15	0	0	0,00
254569520	C4R266	ARL1	Soluble GTPase with a role in regulation of membrane traffic	4,87	20265	0	0	0,00	0	0	0,00	0	0	0,00	0	0	0,00	0	0	0,00	0	0	0,00	1	1	10,17	0	0	0,00
254569526	C4R269	PP7435_Chr2-0333	mitochondrial rhodanese-like protein	8,45	20219	0	0	0,00	0	0	0,00	0	0	0,00	0	0	0,00	0	0	0,00	0	0	0,00	2	1	10,15	0	0	0,00
254569530	C4R271	BNA1	3-hydroxyanthranilic acid dioxygenase, required for the de novo biosynthesis of NAD from tryptophan	5,41	19503	1	1	10,13	1	1	10,11	0	0	0,00	0	0	0,00	1	1	10,11	0	0	0,00	2	2	15,13	0	0	0,00
254569544	C4R278	RKS1	Putative kinase	5,45	81518	1	1	10,20	0	0	0,00	2	3	10,25	1	2	10,20	2	3	10,27	1	1	10,16	2	2	10,27	0	0	0,00
254569568	C4R290	PDA1	E1 alpha subunit of the pyruvate dehydrogenase (PDH) complex	7,94	44003	0	0	0,00	0	0	0,00	1	1	10,17	0	0	0,00	2	1	10,17	0	0	0,00	0	0	0,00	0	0	0,00
254569574	C4R293	CUE5	Protein containing a CUE domain that binds ubiquitin	4,67	37740	0	0	0,00	0	0	0,00	0	0	0,00	0	0	0,00	2	3	20,17	0	0	0,00	2	2	14,18	1	1	10,15
254569582	C4R297	PP7435_Chr2-0304	hypothetical protein	4,60	28257	2	1	10,15	1	1	10,19	2	1	10,19	0	0	0,00	2	2	10,20	2	2	20,19	2	2	15,21	2	2	15,19
254569584	C4R298	CAR1	Arginase, responsible for arginine degradation	5,11	35416	0	0	0,00	0	0	0,00	0	0	0,00	0	0	0,00	2	1	10,18	0	0	0,00	2	2	15,18	2	2	10,23
254569606	C4R2A9	PP7435_Chr2-0291	hypothetical protein	7,46	34670	0	0	0,00	0	0	0,00	0	0	0,00	0	0	0,00	0	0	0,00	0	0	0,00	2	3	20,17	0	0	0,00
254569618	C4R2B5	PP7435_Chr2-0285	B-type regulatory subunit of protein phosphatase 2A (PP2A)	5,25	73828	0	0	0,00	0	0	0,00	0	0	0,00	0	0	0,00	0	0	0,00	0	0	0,00	1	1	10,15	0	0	0,00
254569628	C4R2C0	PP7435_Chr2-0280	Essential structural subunit of the nuclear pore complex (NPC)	4,90	186426	1	1	10,14	0	0	0,00	0	0	0,00	0	0	0,00	0	0	0,00	0	0	0,00	0	0	0,00	0	0	0,00
254569638	C4R2C5	PP7435_Chr2-0276	ATP-binding protein that is a subunit of the HOPS complex and the CORVET tethering complex	6,05	75729	0	0	0,00	0	0	0,00	0	0	0,00	0	0	0,00	0	0	0,00	0	0	0,00	2	1	12,14	0	0	0,00
254569654	C4R2D3	PP7435_Chr2-0270	40S ribosomal protein S20	10,51	13460	1	1	10,15	0	0	0,00	0	0	0,00	0	0	0,00	1	1	10,15	0	0	0,00	1	2	10,15	1	1	10,15
254569674	C4R2E3	PAS_chr2-2_0247	Beta' regulatory subunit of casein kinase 2, a Ser/Thr protein kinase	4,92	25901	0	0	0,00	0	0	0,00	0	0	0,00	0	0	0,00	2	2	15,18	0	0	0,00	0	0	0,00	0	0	0,00
254569682	C4R2E7	TRR1	hypothetical protein	5,10	34343	1	1	10,16	1	1	10,14	2	3	30,24	2	4	30,21	2	18	55,24	2	7	30,25	2	7	45,22	2	12	70,25
254569696	C4R2F4	PP7435_Chr2-0250	Threonyl-tRNA synthetase, essential cytoplasmic protein	6,21	82450	0	0	0,00	0	0	0,00	0	0	0,00	0	0	0,00	2	2	20,13	0	0	0,00	2	1	10,14	2	1	10,14
254569700	C4R2F6	PP7435_Chr2-0248	hypothetical protein	4,77	19057	2	9	25,21	2	10	20,20	2	3	25,16	0	0	0,00	2	7	19,22	2	1	10,14	2	5	10,24	2	10	25,22
254569702	C4R2F7	PP7435_Chr2-0247	hypothetical protein	9,77	15666	0	0	0,00	0	0	0,00	0	0	0,00	0	0	0,00	0	0	0,00	0	0	0,00	1	1	10,18	0	0	0,00
254569714	C4R2G3	PP7435_Chr2-0241	hypothetical protein	4,24	45634	2	5	20,19	2	7	30,19	2	14	50,25	2	4	30,18	2	11	30,24	2	4	20,17	2	16	55,29	2	26	80,29
254569716	C4R2G4	RPL6	60S ribosomal protein L6	5,19	17371	0	0	0,00	0	0	0,00	1	2	10,17	0	0	0,00	1	1	10,14	0	0	0,00	0	0	0,00	0	0	0,00
254569730	C4R2H1	PAS_chr2-2_0221	Nuclear encoded protein required for translation of COX1 mRNA	6,14	49213	0	0	0,00	0	0	0,00	2	1	10,19	0	0	0,00	2	2	10,22	0	0	0,00	0	0	0,00	0	0	0,00
254569734	C4R2H3	TSA1	Thioredoxin peroxidase, acts as both a ribosome-associated and free cytoplasmic antioxidant	4,70	21418	2	4	35,16	2	5	40,18	2	15	70,22	2	8	35,17	2	32	145,23	2	8	50,17	2	42	105,25	2	36	135,23
254569742	C4R2H7	PP7435_Chr2-0227	Type 2C protein phosphatase	4,30	47840	0	0	0,00	1	2	10,17	2	1	10,20	0	0	0,00	2	1	10,17	0	0	0,00	1	1	10,21	0	0	0,00
254569758	C4R2I5	PP7435_Chr2-0218	hypothetical protein	6,66	18573	0	0	0,00	0	0	0,00	0	0	0,00	0	0	0,00	0	0	0,00	0	0	0,00	1	2	10,18	1	2	10,17
254569764	C4R2I8	PP7435_Chr2-0215	hypothetical protein	9,90	17853	0	0	0,00	1	1	10,17	0	0	0,00	0	0	0,00	0	0	0,00	0	0	0,00	0	0	0,00	0	0	0,00
254569768	C4R2J0	PP7435_Chr2-0213	Putative phosphopantothenoylcysteine synthetase (PPCS)	6,34	39601	0	0	0,00	0	0	0,00	0	0	0,00	0	0	0,00	0	0	0,00	0	0	0,00	2	1	10,16	0	0	0,00
254569842	C4R2M7	NAP1	Protein that interacts with mitotic cyclin Clb2p	4,15	48829	0	0	0,00	0	0	0,00	0	0	0,00	0	0	0,00	0	0	0,00	0	0	0,00	0	0	0,00	2	3	20,18
254569852	C4R2N2	PP7435_Chr2-0175	Homo-isocitrate dehydrogenase, an NAD-linked mitochondrial enzyme	5,90	40008	1	1	10,12	0	0	0,00	0	0	0,00	0	0	0,00	0	0	0,00	0	0	0,00	0	0	0,00	0	0	0,00
254569858	C4R2N5	atpD	Beta subunit of the F1 sector of mitochondrial F1F0 ATP synthase	5,01	53974	2	6	49,20	2	9	40,18	2	9	38,18	2	6	20,19	2	17	87,19	2	4	25,16	2	17	85,18	2	15	79,19
254569866	C4R2N9	ADO1	Adenosine kinase, required for the utilization of S-adenosylmethionine (AdoMet)	5,34	37872	1	1	10,18	1	2	20,16	2	2	10,18	0	0	0,00	1	1	10,16	0	0	0,00	0	0	0,00	1	1	10,15
254569874	C4R2P3	MAP2	Methionine aminopeptidase	5,13	50155	0	0	0,00	0	0	0,00	0	0	0,00	0	0	0,00	2	1	10,19	0	0	0,00	2	2	10,19	0	0	0,00
254569876	C4R2P4	PAS_chr2-2_0463	hypothetical protein	5,13	23617	0	0	0,00	0	0	0,00	1	1	10,17	0	0	0,00	0	0	0,00	0	0	0,00	2	2	15,17	0	0	0,00
254569890	C4R2Q1	SIS1	Type II HSP40 co-chaperone that interacts with the HSP70 protein Ssa1p	8,82	36980	0	0	0,00	0	0	0,00	2	1	10,16	1	1	10,15	0	0	0,00	1	1	10,16	2	1	10,13	1	1	10,15
254569896	C4R2Q4	PAS_chr2-2_0148	Cell wall protein that contains a putative GPI-attachment site	3,71	43204	0	0	0,00	0	0	0,00	0	0	0,00	0	0	0,00	1	1	10,14	2	2	15,19	2	8	40,23	2	27	120,23
254569906	C4R2Q9	URA2	Bifunctional carbamoylphosphate synthetase (CPSase)-aspartate transcarbamylase (ATCase)	5,61	244856	0	0	0,00	0	0	0,00	0	0	0,00	0	0	0,00	2	1	10,13	0	0	0,00	0	0	0,00	0	0	0,00
254569928	C4R2S0	NDH	hypothetical protein	8,24	52110	0	0	0,00	0	0	0,00	1	1	10,12	0	0	0,00	0	0	0,00	0	0	0,00	0	0	0,00	0	0	0,00
254569930	C4R2S1	CAT1	Catalase A, breaks down hydrogen peroxide in the peroxisomal matrix formed by acyl-CoA oxidase (Pox1	6,59	57810	0	0	0,00	0	0	0,00	2	1	10,12	1	1	10,12	2	1	10,15	2	3	23,13	2	9	60,22	2	3	19,14
254569936	C4R2S4	STH1	ATPase component of the RSC chromatin remodeling complex	5,99	142743	1	1	10,18	2	2	10,16	0	0	0,00	0	0	0,00	0	0	0,00	0	0	0,00	0	0	0,00	0	0	0,00
254569938	C4R2S5	CCP1	Mitochondrial cytochrome-c peroxidase	6,63	41961	2	4	25,21	2	3	20,21	0	0	0,00	0	0	0,00	2	2	15,18	0	0	0,00	2	4	20,25	1	1	10,19
254569966	C4R2T9	INO1	Inositol 1-phosphate synthase	5,07	58379	2	2	15,15	1	4	20,15	0	0	0,00	0	0	0,00	0	0	0,00	0	0	0,00	0	0	0,00	0	0	0,00
254570028	C4R2X0	PAS_chr2-2_0459	hypothetical protein	6,18	82817	0	0	0,00	0	0	0,00	1	1	10,12	0	0	0,00	0	0	0,00	0	0	0,00	0	0	0,00	0	0	0,00
254570034	C4R2X3	PAS_chr2-2_0082	hypothetical protein	7,64	16056	0	0	0,00	0	0	0,00	1	1	10,17	0	0	0,00	0	0	0,00	0	0	0,00	1	1	10,18	2	14	60,18
254570036	C4R2X4	PAS_chr2-2_0081	Constituent of the mitochondrial inner membrane presequence translocase (TIM23 complex)	8,53	52950	0	0	0,00	0	0	0,00	0	0	0,00	0	0	0,00	0	0	0,00	0	0	0,00	2	2	15,14	0	0	0,00
254570064	C4R2Y8	PP7435_Chr2-0075	hypothetical protein	9,60	19470	0	0	0,00	0	0	0,00	0	0	0,00	0	0	0,00	0	0	0,00	0	0	0,00	2	1	10,14	0	0	0,00
254570072	C4R2Z2	PP7435_Chr2-0071	Protein chaperone involved in regulation of the HSP90 and HSP70 functions	5,78	43370	1	1	10,14	0	0	0,00	2	6	45,20	1	2	10,15	2	9	54,22	2	3	20,21	2	3	15,19	2	3	25,21
254570088	C4R300	YNK1	Nucleoside diphosphate kinase	6,17	16943	2	1	10,13	1	1	10,12	2	3	25,20	2	4	25,20	2	4	25,20	1	1	10,14	2	4	35,18	2	17	85,21
254570090	C4R301	PP7435_Chr2-0060	Eukaryotic initiation factor (eIF) 2A	9,32	68082	2	2	15,25	2	2	15,22	0	0	0,00	0	0	0,00	1	1	10,17	0	0	0,00	0	0	0,00	0	0	0,00
254570098	C4R305	PP7435_Chr2-0056	60S ribosomal protein L10	10,29	25451	1	1	10,12	1	1	10,15	0	0	0,00	0	0	0,00	0	0	0,00	0	0	0,00	1	2	20,16	0	0	0,00
254570100	C4R306	PMM1	Phosphomannomutase, involved in synthesis of GDP-mannose and dolichol-phosphate-mannose	4,94	28659	0	0	0,00	0	0	0,00	1	1	10,16	0	0	0,00	2	1	14,18	0	0	0,00	1	1	10,14	2	1	10,16
254570112	C4R312	PP7435_Chr2-0049	Dihydrolipoamide dehydrogenase	6,31	52306	2	15	55,27	2	6	45,26	2	11	50,23	1	1	10,14	2	19	60,28	2	2	15,15	2	42	95,33	2	56	85,32
254570154	C4R333	PP7435_Chr2-0026	Subunit of the ARP2/3 complex	4,36	16909	0	0	0,00	2	2	10,14	2	3	20,14	2	2	20,12	2	4	25,17	2	1	10,15	1	1	10,12	1	1	10,13
254570192	C4R352	PAS_chr2-2_0011	hypothetical protein	6,59	47320	0	0	0,00	0	0	0,00	0	0	0,00	0	0	0,00	1	1	10,12	0	0	0,00	0	0	0,00	0	0	0,00
254570215	C4R3A2	yahK	NADPH-dependent medium chain alcohol dehydrogenase	7,71	38130	0	0	0,00	0	0	0,00	1	1	10,13	1	1	10,11	2	3	10,14	0	0	0,00	2	2	15,15	1	3	20,14
254570217	C4R3A3	PAS_chr3_0008	hypothetical protein	9,68	50934	0	0	0,00	0	0	0,00	0	0	0,00	0	0	0,00	0	0	0,00	0	0	0,00	0	0	0,00	1	1	10,14
254570271	C4R3D0	LEU2	Beta-isopropylmalate dehydrogenase (IMDH), catalyzes the third step in the leucine biosynthesis path	4,70	39781	1	2	20,15	2	1	10,14	2	2	15,19	0	0	0,00	2	3	21,22	2	4	25,16	2	10	35,23	2	18	40,22
254570279	C4R3D4	PEX2	peroxisomal integral membrane protein	4,71	52231	0	0	0,00	0	0	0,00	1	1	10,12	0	0	0,00	0	0	0,00	0	0	0,00	0	0	0,00	0	0	0,00
254570283	C4R3D6	PRP3	hypothetical protein	9,18	53288	0	0	0,00	0	0	0,00	0	0	0,00	0	0	0,00	0	0	0,00	0	0	0,00	0	0	0,00	1	1	10,14
254570305	C4R3E7	PP7435_Chr3-1188	60S ribosomal protein L20	10,30	21443	0	0	0,00	0	0	0,00	2	1	10,14	0	0	0,00	0	0	0,00	0	0	0,00	0	0	0,00	0	0	0,00
254570321	C4R3F5	PP7435_Chr3-1181	hypothetical protein	5,90	27247	1	1	10,13	0	0	0,00	0	0	0,00	0	0	0,00	0	0	0,00	0	0	0,00	0	0	0,00	0	0	0,00
254570337	C4R3G3	CAT2	Carnitine acetyl-CoA transferase present in both mitochondria and peroxisomes, transfers activated a	7,42	71635	2	4	30,17	2	4	25,20	0	0	0,00	0	0	0,00	2	1	10,19	0	0	0,00	2	3	25,18	1	1	10,15
254570361	C4R3H5	PP7435_Chr3-1158	Putative protein of unknown function	5,77	34632	0	0	0,00	0	0	0,00	0	0	0,00	0	0	0,00	0	0	0,00	0	0	0,00	0	0	0,00	2	1	10,16
254570367	C4R3H8	ENO2	Enolase I, a phosphopyruvate hydratase that catalyzes the conversion of 2-phosphoglycerate to phosph	5,33	46481	2	133	155,20	2	111	145,21	2	28	125,22	2	5	40,20	2	37	135,23	2	21	130,21	2	20	123,21	2	34	150,22
254570373	C4R3I1	ade6	Phosphoribosylaminoimidazole carboxylase, catalyzes a step in the 'de novo' purine nucleotide biosyn	6,22	61366	0	0	0,00	0	0	0,00	2	4	20,16	2	3	10,15	2	2	10,17	2	2	15,14	1	1	10,15	2	1	10,16
254570375	C4R3I2	PP7435_Chr3-1150	Peroxisomal 2,4-dienoyl-CoA reductase, auxiliary enzyme of fatty acid beta-oxidation	5,39	28309	0	0	0,00	0	0	0,00	0	0	0,00	0	0	0,00	0	0	0,00	2	1	10,18	2	1	10,14	2	1	10,18
254570407	C4R3J8	PP7435_Chr3-1133	hypothetical protein	6,36	21157	0	0	0,00	0	0	0,00	0	0	0,00	0	0	0,00	0	0	0,00	0	0	0,00	2	2	15,23	0	0	0,00
254570411	C4R3K0	PFY1	Profilin, actin-and phosphatidylinositol 4,5-bisphosphate-binding protein, involved in cytoskeleton	5,15	13783	0	0	0,00	1	1	10,13	2	1	10,11	0	0	0,00	0	0	0,00	1	1	10,11	2	3	15,21	2	2	15,20
254570413	C4R3K1	LEO1	hypothetical protein	4,17	48857	0	0	0,00	0	0	0,00	0	0	0,00	0	0	0,00	0	0	0,00	0	0	0,00	1	1	10,14	0	0	0,00
254570417	C4R3K3	PP7435_Chr3-1128	Protein required for Golgi localization of glycosyltransferases	6,26	33145	0	0	0,00	0	0	0,00	0	0	0,00	0	0	0,00	1	1	10,26	0	0	0,00	0	0	0,00	1	1	10,23
254570431	C4R3L0	BCS1	Protein of the mitochondrial inner membrane that functions as an ATP-dependent chaperone, required f	8,07	50971	0	0	0,00	0	0	0,00	0	0	0,00	0	0	0,00	0	0	0,00	0	0	0,00	0	0	0,00	1	1	10,12
254570435	C4R3L2	TFC6	One of six subunits of RNA polymerase III transcription initiation factor complex (TFIIIC)	5,66	70034	0	0	0,00	0	0	0,00	0	0	0,00	0	0	0,00	1	1	10,14	0	0	0,00	0	0	0,00	0	0	0,00
254570459	C4R3M4	PP7435_Chr3-1106	Essential nucleolar protein that is a component of the SSU (small subunit) processome involved in th	10,29	21648	0	0	0,00	0	0	0,00	0	0	0,00	0	0	0,00	0	0	0,00	0	0	0,00	1	1	10,10	0	0	0,00
254570461	C4R3M5	RUP1	hypothetical protein	4,41	75014	0	0	0,00	0	0	0,00	0	0	0,00	0	0	0,00	0	0	0,00	0	0	0,00	2	1	10,18	0	0	0,00
254570491	C4R3P0	TEL2	Essential DNA-binding protein specific to single-stranded yeast telomeric DNA repeats, required for	5,63	98815	0	0	0,00	0	0	0,00	0	0	0,00	0	0	0,00	0	0	0,00	1	1	10,14	0	0	0,00	0	0	0,00
254570497	C4R3P3	PAS_chr3_0150	GTP-binding protein of the rho subfamily of Ras-like proteins, involved in establishment of cell pol	5,22	21998	0	0	0,00	0	0	0,00	1	1	10,12	0	0	0,00	2	1	10,14	1	1	10,13	2	2	20,17	2	3	10,16
254570503	C4R3P6	PAS_chr3_0154	Putative protein of unknown function	5,86	60835	0	0	0,00	0	0	0,00	0	0	0,00	0	0	0,00	0	0	0,00	0	0	0,00	1	1	10,17	0	0	0,00
254570513	C4R3Q1	BRX1	Nucleolar protein, constituent of 66S pre-ribosomal particles	9,30	33661	2	1	10,14	0	0	0,00	2	2	10,17	0	0	0,00	0	0	0,00	0	0	0,00	0	0	0,00	0	0	0,00
254570517	C4R3Q3	AVO1	Component of a membrane-bound complex containing the Tor2p kinase and other proteins	6,45	71420	0	0	0,00	0	0	0,00	0	0	0,00	0	0	0,00	0	0	0,00	0	0	0,00	1	1	10,17	0	0	0,00
254570535	C4R3R2	HCH1	Co-chaperone that binds to Hsp82p and activates its ATPase activity	4,66	17087	0	0	0,00	0	0	0,00	1	2	10,16	0	0	0,00	1	2	10,16	0	0	0,00	0	0	0,00	1	1	10,16
254570537	C4R3R3	PAS_chr3_0171	hypothetical protein	6,74	23027	0	0	0,00	0	0	0,00	1	1	10,15	0	0	0,00	0	0	0,00	0	0	0,00	0	0	0,00	0	0	0,00
254570575	C4R3T2	PDC1	Major of three pyruvate decarboxylase isozymes	5,58	61304	2	34	132,27	2	40	148,25	2	29	165,28	2	15	85,23	2	34	165,28	2	15	104,22	2	53	160,31	2	31	95,30
254570591	C4R3U0	GET2	hypothetical protein	5,33	34354	0	0	0,00	0	0	0,00	2	1	10,22	0	0	0,00	2	2	10,26	0	0	0,00	0	0	0,00	1	1	10,15
254570599	C4R3U4	PP7435_Chr3-1029	Protein involved in 5.8S rRNA processing	9,00	55276	0	0	0,00	0	0	0,00	0	0	0,00	0	0	0,00	1	1	10,14	0	0	0,00	0	0	0,00	0	0	0,00
254570607	C4R3U8	ffr	hypothetical protein	4,30	33840	1	1	10,15	0	0	0,00	0	0	0,00	0	0	0,00	0	0	0,00	0	0	0,00	1	1	10,19	0	0	0,00
254570633	C4R3W1	ARG1	Arginosuccinate synthetase	5,04	46380	2	4	24,16	2	3	15,15	2	11	60,20	2	3	25,18	2	4	30,19	2	3	30,16	2	4	35,19	2	2	20,20
254570655	C4R3X2	OSM1	Soluble fumarate reductase	5,52	52707	0	0	0,00	1	2	20,14	0	0	0,00	0	0	0,00	0	0	0,00	0	0	0,00	0	0	0,00	0	0	0,00
254570661	C4R3X5	SLB2	Primary component of eisosomes	4,69	33893	2	6	30,19	2	8	30,21	2	2	10,15	2	2	15,16	2	2	10,17	2	2	15,14	2	2	24,14	2	10	59,21
254570663	C4R3X6	PP7435_Chr3-0998	Catalytic subunit of the Hat1p-Hat2p histone acetyltransferase complex	5,07	44375	0	0	0,00	0	0	0,00	0	0	0,00	0	0	0,00	0	0	0,00	0	0	0,00	1	1	10,14	0	0	0,00
254570667	C4R3X8	PP7435_Chr3-0996	ATPase involved in protein folding and the response to stress	4,95	70706	2	22	114,22	2	14	45,20	2	32	105,21	2	25	70,17	2	22	90,25	2	22	51,19	2	21	113,21	2	27	114,25
254570673	Q9P4D1	ACT1	hypothetical protein	5,16	41647	2	3	25,19	2	1	10,20	2	2	15,16	1	1	10,13	2	3	20,17	2	2	20,19	2	14	90,20	2	3	34,18
254570679	C4R3Y4	PCNA	Proliferating cell nuclear antigen (PCNA)	4,37	28335	0	0	0,00	0	0	0,00	0	0	0,00	0	0	0,00	0	0	0,00	0	0	0,00	2	1	10,17	0	0	0,00
254570683	C4R3Y6	PAS_chr3_0235	Zinc-finger protein of unknown function	9,70	31891	1	1	10,15	0	0	0,00	0	0	0,00	0	0	0,00	0	0	0,00	0	0	0,00	0	0	0,00	0	0	0,00
254570719	C4R404	ACH1	Acetyl-coA hydrolase	5,90	58183	2	17	20,27	2	20	20,26	2	9	10,24	2	6	15,20	2	14	20,27	2	5	25,19	2	10	15,27	2	13	10,25
254570727	C4R408	ADK1	Adenylate kinase	7,20	27999	0	0	0,00	1	1	10,14	0	0	0,00	0	0	0,00	1	1	10,14	0	0	0,00	2	1	10,15	0	0	0,00
254570735	C4R412	PP7435_Chr3-0962	hypothetical protein	4,49	38991	2	1	10,15	2	1	10,16	0	0	0,00	0	0	0,00	1	1	10,15	1	1	10,11	2	2	10,18	0	0	0,00
254570763	C4R426	PAS_chr3_0273	Non-essential hydrolase involved in mRNA decapping	6,17	35517	0	0	0,00	0	0	0,00	1	1	10,11	0	0	0,00	1	1	10,12	0	0	0,00	2	2	15,18	1	2	20,19
254570765	C4R427	PAS_chr3_0274	R-SNARE protein	9,54	24741	0	0	0,00	0	0	0,00	0	0	0,00	0	0	0,00	0	0	0,00	1	1	10,11	0	0	0,00	0	0	0,00
254570771	C4R430	PGD	6-phosphogluconate dehydrogenase (decarboxylating)	5,87	53929	2	23	70,30	2	20	80,27	2	25	35,24	2	5	25,23	2	36	60,26	2	6	30,21	2	24	79,26	2	21	39,23
254570789	C4R439	PP7435_Chr3-0935	Glutamine tRNA synthetase	6,61	91762	0	0	0,00	0	0	0,00	0	0	0,00	0	0	0,00	2	2	19,17	0	0	0,00	2	3	15,16	0	0	0,00
254570807	C4R448	LTV1	hypothetical protein	4,41	48750	2	1	10,21	1	1	10,18	2	1	10,20	1	1	10,18	2	1	10,21	2	2	15,23	0	0	0,00	1	1	10,21
254570839	C4R464	PP7435_Chr3-0906	UBX (ubiquitin regulatory X) domain-containing protein	4,13	49872	0	0	0,00	0	0	0,00	0	0	0,00	0	0	0,00	1	1	10,21	0	0	0,00	0	0	0,00	0	0	0,00
254570897	C4R493	RPG1	Subunit of the core complex of translation initiation factor 3(eIF3)	6,65	97284	0	0	0,00	0	0	0,00	1	2	24,18	0	0	0,00	2	1	17,18	0	0	0,00	2	3	10,16	2	3	15,15
254570901	C4R495	NSF	ATPase required for the release of Sec17p	6,50	83977	0	0	0,00	0	0	0,00	0	0	0,00	0	0	0,00	0	0	0,00	0	0	0,00	2	2	10,16	0	0	0,00
254570941	C4R4B5	PP7435_Chr3-0857	Ubiquitin-conjugating enzyme (E2)	4,04	19722	0	0	0,00	0	0	0,00	0	0	0,00	0	0	0,00	1	1	10,12	0	0	0,00	0	0	0,00	0	0	0,00
254570945	C4R4B7	PP7435_Chr3-0855	Nicotinamidase	5,14	24615	1	2	20,13	2	5	20,16	0	0	0,00	0	0	0,00	0	0	0,00	0	0	0,00	1	2	10,15	1	1	10,11

254570949	C4R4B9	PAS_chr3_0362	hypothetical protein	5,67	32554	0	0	0,00	0	0	0,00	0	0	0,00	0	0	0,00	0	0	0,00	2	2	15,14	2	4	10,15	1	1	10,15
254570957	C4R4C3	SSC1	Mitochondrial matrix ATPase	5,28	69648	2	16	75,25	2	9	50,26	2	30	120,31	2	10	70,21	2	84	185,33	2	14	90,17	2	12	60,15	2	11	40,18
254570979	C4R4D4	GARS	Cytoplasmic and mitochondrial glycyl-tRNA synthase	5,16	74111	2	12	15,18	2	11	10,18	2	20	45,21	2	1	10,13	2	13	25,19	2	14	40,18	2	25	50,25	2	12	35,21
254570981	C4R4D5	PP7435_Chr3-0838	hypothetical protein	6,40	45175	0	0	0,00	0	0	0,00	2	4	30,21	2	5	35,15	2	13	60,20	2	5	39,18	2	6	50,21	2	15	105,22
254570983	C4R4D6	PP7435_Chr3-0837	Widely conserved NADPH oxidoreductase containing flavin mononucleotide (FMN)	6,15	45407	0	0	0,00	0	0	0,00	2	2	20,18	2	3	25,18	2	7	40,21	2	2	20,20	2	2	24,20	2	11	75,21
254571007	C4R4E8	PAS_chr3_0388	Methionine aminopeptidase	5,78	41114	0	0	0,00	0	0	0,00	0	0	0,00	0	0	0,00	1	1	10,20	0	0	0,00	0	0	0,00	0	0	0,00
254571015	C4R4F2	GCD6	Catalytic epsilon subunit of the translation initiation factor eIF2B	4,67	72539	0	0	0,00	0	0	0,00	0	0	0,00	0	0	0,00	0	0	0,00	0	0	0,00	0	0	0,00	2	1	10,13
254571031	C4R4G0	ERF2	Translation termination factor eRF3	8,51	78161	0	0	0,00	0	0	0,00	1	1	10,17	0	0	0,00	1	1	10,24	0	0	0,00	2	1	10,18	0	0	0,00
254571043	C4R4G6	acsA	Acetyl-coA synthetase isoform	5,43	74000	1	1	10,13	0	0	0,00	2	1	10,14	0	0	0,00	2	2	15,17	2	2	15,13	2	4	35,24	0	0	0,00
254571057	C4R4H3	CAR2	L-ornithine transaminase (OTAse)	5,88	47097	2	24	65,22	2	26	70,22	2	9	50,25	2	5	40,17	2	16	65,24	2	5	45,16	2	26	80,25	2	10	50,23
254571059	C4R4H4	PAS_chr3_0411	hypothetical protein	4,72	15428	0	0	0,00	0	0	0,00	0	0	0,00	0	0	0,00	2	1	10,13	0	0	0,00	0	0	0,00	0	0	0,00
254571065	C4R4H7	PAS_chr3_0414	Calcineurin A	6,47	66340	2	1	10,20	2	1	10,21	0	0	0,00	0	0	0,00	0	0	0,00	0	0	0,00	0	0	0,00	0	0	0,00
254571075	C4R4I2	PAS_chr3_0419	Putative metalloprotease with similarity to the zinc carboxypeptidase family	5,27	62312	2	1	10,13	0	0	0,00	0	0	0,00	0	0	0,00	2	1	10,12	0	0	0,00	0	0	0,00	2	2	10,17
254571095	C4R4J2	PP7435_Chr3-0784	Protein required for cytochrome c oxidase assembly	7,80	11315	0	0	0,00	0	0	0,00	1	1	10,14	1	1	10,15	1	2	10,17	2	3	10,17	2	2	10,18	2	3	10,19
254571099	C4R4J4	PAS_chr3_0429	Protein required for transport of flavin adenine dinucleotide (FAD)	9,75	34062	0	0	0,00	0	0	0,00	0	0	0,00	0	0	0,00	0	0	0,00	0	0	0,00	1	1	10,18	0	0	0,00
254571103	C4R4J6	PP7435_Chr3-0780	Evolutionarily-conserved 5'-3' exonuclease component of cytoplasmic processing (P) bodies	6,39	164916	0	0	0,00	0	0	0,00	0	0	0,00	0	0	0,00	0	0	0,00	0	0	0,00	1	1	10,16	0	0	0,00
254571125	C4R4K7	PP7435_Chr3-0765	Putative dihydrokaempferol 4-reductase	6,02	39071	0	0	0,00	0	0	0,00	2	7	46,21	2	3	20,18	2	13	70,20	2	18	70,20	2	20	80,22	2	20	84,20
254571127	C4R4K8	PP7435_Chr3-0764	Putative dihydrokaempferol 4-reductase	5,75	39489	0	0	0,00	0	0	0,00	2	1	13,18	1	1	18,15	0	0	0,00	2	6	26,20	2	11	31,28	2	4	23,21
254571129	C4R4K9	PP7435_Chr3-0763	Putative dihydrokaempferol 4-reductase	5,73	39227	0	0	0,00	0	0	0,00	0	0	0,00	0	0	0,00	0	0	0,00	1	1	10,16	0	0	0,00	0	0	0,00
254571131	C4R4L0	PP7435_Chr3-0762	Putative dihydrokaempferol 4-reductase	6,02	39202	0	0	0,00	0	0	0,00	2	4	30,17	2	2	10,18	2	5	30,20	2	15	70,19	2	36	125,24	2	42	123,22
254571133	C4R4L1	PP7435_Chr3-0761	Alpha 4 subunit of the 20S proteasome	6,42	27783	0	0	0,00	0	0	0,00	0	0	0,00	0	0	0,00	0	0	0,00	0	0	0,00	0	0	0,00	2	2	11,19
254571137	C4R4L3	RTG2	Sensor of mitochondrial dysfunction	7,14	62778	0	0	0,00	0	0	0,00	0	0	0,00	0	0	0,00	0	0	0,00	0	0	0,00	1	1	10,15	0	0	0,00
254571145	C4R4L7	PGI1	Glycolytic enzyme phosphoglucose isomerase	5,81	61928	2	20	125,38	2	14	75,36	2	1	10,18	1	2	20,16	1	2	20,18	2	3	10,15	2	20	50,20	2	11	55,19
254571153	C4R4M1	PP7435_Chr3-0750	Essential component of the Arp2/3 complex	5,68	43993	0	0	0,00	1	1	10,13	0	0	0,00	0	0	0,00	0	0	0,00	0	0	0,00	0	0	0,00	0	0	0,00
254571157	C4R4M3	ATP4	Subunit b of the stator stalk of mitochondrial F1F0 ATP synthase	9,31	25326	0	0	0,00	0	0	0,00	0	0	0,00	0	0	0,00	0	0	0,00	0	0	0,00	1	1	10,14	0	0	0,00
254571161	C4R4M5	ALA1	Cytoplasmic alanyl-tRNA synthetase	5,36	109024	0	0	0,00	0	0	0,00	2	2	15,17	0	0	0,00	2	2	15,15	2	1	10,16	2	1	10,16	0	0	0,00
254571179	C4R4N4	HOM2	Aspartic beta semi-aldehyde dehydrogenase	5,37	38650	2	9	20,18	2	9	20,20	2	4	19,18	1	1	10,13	2	6	35,19	0	0	0,00	2	2	10,19	2	7	30,20
254571181	C4R4N5	PP7435_Chr3-0735	hypothetical protein	8,78	25487	0	0	0,00	0	0	0,00	1	1	10,17	0	0	0,00	1	1	10,18	0	0	0,00	2	2	20,18	1	1	10,18
254571293	C4R4U1	LYS9	Saccharopine dehydrogenase (NADP+, L-glutamate-forming)	4,98	48659	2	1	10,12	2	1	10,11	2	3	25,14	2	1	10,14	2	2	20,14	0	0	0,00	0	0	0,00	2	1	10,15
254571299	C4R4U4	GDI1	GDP dissociation inhibitor	5,32	50664	0	0	0,00	0	0	0,00	0	0	0,00	0	0	0,00	0	0	0,00	0	0	0,00	1	2	10,16	0	0	0,00
254571313	C4R4V1	PP7435_Chr3-0667	Peripheral mitochondrial membrane protein involved in mitochondrial protein import	9,75	46023	0	0	0,00	0	0	0,00	0	0	0,00	0	0	0,00	0	0	0,00	0	0	0,00	1	1	10,13	0	0	0,00
254571321	C4R4V5	PP7435_Chr3-0662	Zinc transporter	4,77	47694	0	0	0,00	0	0	0,00	0	0	0,00	0	0	0,00	0	0	0,00	0	0	0,00	1	1	10,14	0	0	0,00
254571327	C4R4V8	BCY1	Regulatory subunit of the cyclic AMP-dependent protein kinase (PKA)	5,35	44673	0	0	0,00	0	0	0,00	0	0	0,00	0	0	0,00	1	1	10,15	1	1	10,12	1	1	10,14	0	0	0,00
254571353	C4R4X1	GLO3	ADP-ribosylation factor GTPase activating protein (ARF GAP)	8,16	48179	2	2	10,24	2	1	10,20	0	0	0,00	0	0	0,00	0	0	0,00	0	0	0,00	0	0	0,00	0	0	0,00
254571359	C4R4X4	TUF1	Mitochondrial translation elongation factor Tu	5,54	46770	1	1	10,14	0	0	0,00	2	6	55,21	2	3	25,16	2	3	15,18	2	6	34,20	2	9	65,18	2	5	50,17
254571365	C4R4X7	PAS_chr3_0565	hypothetical protein	6,67	34113	1	1	10,14	0	0	0,00	0	0	0,00	0	0	0,00	1	1	10,15	0	0	0,00	0	0	0,00	0	0	0,00
254571367	C4R4X8	PAS_chr3_0566	3-phosphoserine aminotransferase	6,02	42298	0	0	0,00	0	0	0,00	0	0	0,00	0	0	0,00	0	0	0,00	0	0	0,00	1	1	10,15	2	1	10,13
254571369	C4R4X9	CPR6	Peptidyl-prolyl cis-trans isomerase (cyclophilin)	5,12	40035	2	3	30,19	2	2	15,17	2	6	35,21	2	7	30,26	1	1	10,19	2	7	40,21	2	1	10,20	2	2	20,16
254571371	C4R4Y0	PP7435_Chr3-0638	RNA polymerase II largest subunit B220	5,32	193757	0	0	0,00	1	1	10,11	0	0	0,00	0	0	0,00	0	0	0,00	0	0	0,00	1	1	10,18	0	0	0,00
254571387	C4R4Y8	atpA	Alpha subunit of the F1 sector of mitochondrial F1F0 ATP synthase	9,39	58713	2	2	15,13	2	3	10,13	2	3	20,13	2	2	10,15	2	6	20,14	2	2	20,13	2	7	40,14	2	9	25,16
254571393	C4R4Z1	GUT2	Mitochondrial glycerol-3-phosphate dehydrogenase	6,14	74167	2	3	10,20	2	3	10,20	2	2	10,18	0	0	0,00	2	2	15,19	0	0	0,00	2	4	10,24	2	2	15,20
254571395	C4R4Z2	RRP4	3'-5' exoribonuclease involved in rRNA processing	6,14	38322	0	0	0,00	0	0	0,00	0	0	0,00	1	1	10,14	0	0	0,00	0	0	0,00	0	0	0,00	0	0	0,00
254571401	C4R4Z5	PP7435_Chr3-0624	Component of the TOM (translocase of outer membrane) complex	9,46	19365	0	0	0,00	0	0	0,00	0	0	0,00	0	0	0,00	0	0	0,00	0	0	0,00	1	1	10,23	1	1	10,18
254571421	C4R505	PP7435_Chr3-0611	F-box protein containing five copies of the WD40 motif, controls cell cycle function	7,91	70171	0	0	0,00	0	0	0,00	1	1	10,13	0	0	0,00	1	1	10,14	1	1	10,12	1	1	10,12	0	0	0,00
254571423	C4R506	PAS_chr3_0595	Translation initiation factor eIF4A, identical to Tif1p	4,81	44994	0	0	0,00	0	0	0,00	1	1	10,18	0	0	0,00	2	2	15,18	0	0	0,00	2	3	25,20	0	0	0,00
254571447	C4R518	ORF3	hypothetical protein	6,53	23993	0	0	0,00	0	0	0,00	1	1	10,17	0	0	0,00	0	0	0,00	0	0	0,00	1	1	10,22	0	0	0,00
254571455	C4R522	SRP1	Karyopherin alpha homolog, forms a dimer with karyopherin beta Kap95p	4,52	61160	0	0	0,00	0	0	0,00	0	0	0,00	0	0	0,00	0	0	0,00	0	0	0,00	1	1	10,14	0	0	0,00
254571463	C4R526	PP7435_Chr3-0588	Subunit Va of cytochrome c oxidase	10,14	17603	0	0	0,00	0	0	0,00	0	0	0,00	0	0	0,00	0	0	0,00	0	0	0,00	1	1	10,11	0	0	0,00
254571471	C4R530	PAS_chr3_0619	hypothetical protein	7,90	24267	0	0	0,00	0	0	0,00	0	0	0,00	0	0	0,00	0	0	0,00	0	0	0,00	0	0	0,00	1	1	10,16
254571485	C4R537	CRP1	Protein that binds to cruciform DNA structures	4,80	32192	2	3	15,15	2	3	15,13	1	1	10,14	1	1	10,13	0	0	0,00	0	0	0,00	2	1	10,15	1	1	10,15
254571503	C4R546	PP7435_Chr3-0568	Putative serine type carboxypeptidase with a role in phytochelatin synthesis	4,74	61016	0	0	0,00	0	0	0,00	0	0	0,00	0	0	0,00	0	0	0,00	0	0	0,00	1	1	10,19	0	0	0,00
254571505	C4R547	trpB	Tryptophan synthase involved in tryptophan biosynthesis	5,19	44910	0	0	0,00	0	0	0,00	0	0	0,00	0	0	0,00	0	0	0,00	0	0	0,00	1	1	10,11	2	1	10,12
254571517	C4R553	PAS_chr3_0640	Carboxypeptidase Y inhibitor, function requires acetylation by the NatB N-terminal acetyltransferase	8,44	29722	2	2	10,15	2	1	10,12	2	3	10,19	0	0	0,00	2	3	10,21	0	0	0,00	2	4	15,20	2	4	10,21
254571529	C4R559	PP7435_Chr3-0553	Fumarase, converts fumaric acid to L-malic acid in the TCA cycle	6,42	52570	2	3	15,21	2	2	10,22	2	4	10,23	0	0	0,00	2	5	20,22	2	1	10,15	2	7	15,24	2	10	15,25
254571543	C4R566	PAS_chr3_0655	Phosphatidylinositol/phosphatidylcholine transfer protein	6,39	38442	2	2	10,17	2	1	10,17	1	1	10,23	0	0	0,00	0	0	0,00	0	0	0,00	2	2	10,18	2	1	10,19
254571547	C4R568	PAS_chr3_0657	ATP-dependent RNA helicase of the SFI superfamily, required for nonsense mediated mRNA decay and for	6,41	104819	1	1	10,14	0	0	0,00	0	0	0,00	0	0	0,00	0	0	0,00	0	0	0,00	0	0	0,00	0	0	0,00
254571579	C4R584	PP7435_Chr3-0526	Predominantly nucleolar DEAH-box ATP-dependent RNA helicase, required for 18S rRNA synthesis	6,94	93362	0	0	0,00	0	0	0,00	2	1	10,15	0	0	0,00	0	0	0,00	0	0	0,00	0	0	0,00	0	0	0,00
254571583	C4R586	PAS_chr3_0674	D-lactate dehydrogenase, located in the mitochondrial matrix	5,79	59483	2	4	20,20	2	3	20,20	2	3	15,19	0	0	0,00	2	4	20,23	2	1	10,10	2	4	25,23	2	13	60,24
254571585	C4R587	PP7435_Chr3-0523	hypothetical protein	4,48	59647	0	0	0,00	0	0	0,00	0	0	0,00	0	0	0,00	0	0	0,00	0	0	0,00	2	1	10,18	0	0	0,00
254571587	C4R588	ASN1	Asparagine synthetase, isozyme of Asn1p	5,53	65032	2	2	20,24	2	2	15,23	2	6	30,22	1	2	10,18	2	4	20,21	2	1	10,13	2	8	35,24	2	8	45,24
254571589	C4R589	PAS_chr3_0676	UDP-N-acetylglucosamine pyrophosphorylase	5,54	45473	0	0	0,00	0	0	0,00	2	1	10,12	0	0	0,00	0	0	0,00	0	0	0,00	0	0	0,00	0	0	0,00
254571619	C4R5A4	PAS_chr3_0691	Possible chaperone and cysteine protease with similarity to E. coli Hsp31	5,97	25375	0	0	0,00	0	0	0,00	0	0	0,00	2	1	10,15	2	2	20,20	0	0	0,00	1	1	10,17	2	1	10,18
254571635	C4R5B2	PAS_chr3_0699	Alpha 7 subunit of the 20S proteasome	4,60	28387	2	1	10,12	2	1	10,14	0	0	0,00	0	0	0,00	0	0	0,00	0	0	0,00	0	0	0,00	0	0	0,00
254571639	C4R5B4	PRT1	Subunit of the core complex of translation initiation factor 3(eIF3), essential for translation	5,66	82067	0	0	0,00	0	0	0,00	0	0	0,00	0	0	0,00	0	0	0,00	0	0	0,00	2	2	15,16	0	0	0,00
254571661	C4R5C5	PP7435_Chr3-0487	Ubiquitin-conjugating enzyme that mediates selective degradation of short-lived and abnormal protein	6,48	16309	0	0	0,00	0	0	0,00	0	0	0,00	0	0	0,00	0	0	0,00	1	1	10,13	1	2	20,16	0	0	0,00
254571663	C4R5C6	CPH2	Basic helix-loop-helix (bHLH) protein with similarity to myc-family transcription factors	5,50	108482	0	0	0,00	1	1	10,18	0	0	0,00	0	0	0,00	0	0	0,00	0	0	0,00	0	0	0,00	0	0	0,00
254571699	C4R5E4	PP7435_Chr3-0464	Cytoplasmic ATPase that is a ribosome-associated molecular chaperone	4,94	66447	2	8	35,21	2	3	20,20	2	7	25,25	1	2	20,19	2	12	30,25	2	2	10,16	0	0	0,00	0	0	0,00
254571725	C4R5F7	PP7435_Chr3-0449	Nit protein, one of two proteins in S. cerevisiae with similarity to the Nit domain	5,81	32127	0	0	0,00	0	0	0,00	0	0	0,00	0	0	0,00	0	0	0,00	0	0	0,00	1	1	10,23	0	0	0,00
254571727	C4R5F8	PP7435_Chr3-0448	Aldose reductase involved in methylglyoxal, d-xylose and arabinose metabolism	5,86	36095	1	1	10,13	1	1	10,12	2	1	10,12	0	0	0,00	2	2	15,12	0	0	0,00	2	6	40,15	2	6	20,14
254571733	C4R5G1	PP7435_Chr3-0446	hypothetical protein	6,82	61260	1	1	10,17	2	1	10,20	2	3	10,22	2	2	15,20	1	2	20,25	1	1	10,17	2	2	10,26	0	0	0,00
254571735	C4R5G2	PP7435_Chr3-0445	Beta 1 subunit of the 20S proteasome, responsible for cleavage after acidic residues in peptides	6,22	23231	0	0	0,00	0	0	0,00	1	1	10,12	0	0	0,00	1	1	10,13	0	0	0,00	1	1	10,13	0	0	0,00
254571739	C4R5G4	PP7435_Chr3-0443	Dynamin-related GTPase	5,62	88440	0	0	0,00	0	0	0,00	0	0	0,00	0	0	0,00	0	0	0,00	0	0	0,00	2	1	10,15	0	0	0,00
254571747	C4R5G8	PAS_chr3_0754	Mitochondrial and cytoplasmic valyl-tRNA synthetase	6,33	121579	0	0	0,00	0	0	0,00	2	1	10,17	0	0	0,00	2	1	10,16	0	0	0,00	2	2	15,23	2	2	10,19
254571775	C4R5I2	PP7435_Chr3-0426	Nuclear envelope protein that mediates the nuclear export of importin alpha (Srp1p)	4,86	110607	0	0	0,00	0	0	0,00	0	0	0,00	0	0	0,00	0	0	0,00	0	0	0,00	2	1	10,15	0	0	0,00
254571779	C4R5I4	SMI1	Protein involved in the regulation of cell wall synthesis	4,58	49548	0	0	0,00	0	0	0,00	1	1	10,13	0	0	0,00	0	0	0,00	0	0	0,00	0	0	0,00	0	0	0,00
254571815	C4R5K2	PP7435_Chr3-0409	Cytoplasmic glyoxalase II	5,70	28415	1	1	10,13	1	1	10,14	0	0	0,00	0	0	0,00	1	2	20,19	1	1	10,12	2	2	15,17	2	1	10,15
254571823	C4R5K6	HIS3	Imidazoleglycerol-phosphate dehydratase, catalyzes the sixth step in histidine biosynthesis	6,15	24133	0	0	0,00	0	0	0,00	1	1	10,13	0	0	0,00	2	4	20,23	0	0	0,00	0	0	0,00	2	1	10,16
254571845	C4R5L7	carB	Large subunit of carbamoyl phosphate synthetase	5,11	125841	2	3	20,25	2	3	25,25	0	0	0,00	0	0	0,00	0	0	0,00	0	0	0,00	1	3	20,21	1	1	10,15
254571849	C4R5L9	PP7435_Chr3-0386	hypothetical protein	9,67	86923	0	0	0,00	0	0	0,00	0	0	0,00	0	0	0,00	1	1	10,15	0	0	0,00	0	0	0,00	0	0	0,00
254571861	C4R5M5	NUC1	hypothetical protein	5,87	54808	0	0	0,00	0	0	0,00	1	1	10,14	0	0	0,00	0	0	0,00	0	0	0,00	2	5	30,16	0	0	0,00
254571869	C4R5M9	PP7435_Chr3-0376	hypothetical protein	4,84	48192	0	0	0,00	0	0	0,00	0	0	0,00	0	0	0,00	0	0	0,00	0	0	0,00	0	0	0,00	1	1	10,13
254571875	C4R5N2	PP7435_Chr3-0372	Protein of unknown function involved in vacuolar protein sorting	6,47	94532	2	3	25,18	2	2	20,21	1	1	10,17	0	0	0,00	2	3	30,22	1	1	10,13	2	10	55,18	2	4	25,17
254571887	C4R5N8	PXA1	Subunit of a heterodimeric peroxisomal ATP-binding cassette transporter complex (Pxa1p-Pxa2p)	9,66	101272	0	0	0,00	0	0	0,00	0	0	0,00	0	0	0,00	0	0	0,00	1	1	10,12	0	0	0,00	0	0	0,00
254571889	C4R5N9	MAS2	hypothetical protein	6,11	52677	0	0	0,00	0	0	0,00	0	0	0,00	0	0	0,00	0	0	0,00	0	0	0,00	1	1	10,14	0	0	0,00
254571895	C4R5P2	PAS_chr3_1224	hypothetical protein	9,67	96814	0	0	0,00	0	0	0,00	0	0	0,00	1	1	10,13	1	1	10,10	0	0	0,00	0	0	0,00	0	0	0,00
254571897	C4R5P3	NSP1	Essential component of the nuclear pore complex, which mediates nuclear import and export	5,05	81051	0	0	0,00	0	0	0,00	0	0	0,00	0	0	0,00	0	0	0,00	0	0	0,00	0	0	0,00	1	1	10,15
254571899	C4R5P4	GPM1	Tetrameric phosphoglycerate mutase	6,01	27966	2	5	20,16	2	5	25,14	0	0	0,00	1	1	10,15	2	2	15,15	2	5	20,18	2	1	10,14	2	2	15,16
254571901	C4R5P5	PP7435_Chr3-0356	hypothetical protein	4,73	14288	0	0	0,00	0	0	0,00	0	0	0,00	0	0	0,00	0	0	0,00	0	0	0,00	1	1	10,14	0	0	0,00
254571907	C4R5P8	PAS_chr3_0832	Transketolase, similar to Tkl2p	6,38	78785	0	0	0,00	0	0	0,00	0	0	0,00	0	0	0,00	0	0	0,00	0	0	0,00	1	1	10,13	0	0	0,00
254571919	C4R5Q4	PP7435_Chr3-0345	Gamma subunit of the translation initiation factor eIF2	5,45	57473	0	0	0,00	0	0	0,00	2	3	20,17	2	2	15,17	0	0	0,00	0	0	0,00	0	0	0,00	0	0	0,00
254571923	C4R5Q6	DAK	Dihydroxyacetone kinase, required for detoxification of dihydroxyacetone (DHA)	5,25	65254	0	0	0,00	0	0	0,00	0	0	0,00	0	0	0,00	1	1	10,15	0	0	0,00	1	1	10,15	1	1	10,13
254571933	C4R5R1	RAT1	Nuclear 5' to 3' single-stranded RNA exonuclease, involved in RNA metabolism	6,52	113955	0	0	0,00	0	0	0,00	0	0	0,00	0	0	0,00	1	1	10,18	0	0	0,00	0	0	0,00	0	0	0,00
254571941	C4R5R5	PAS_chr3_1226	hypothetical protein	4,70	117713	0	0	0,00	1	1	10,15	0	0	0,00	0	0	0,00	0	0	0,00	1	1	10,19	1	1	10,12	0	0	0,00
254571953	C4R5S1	MAS1	Smaller subunit of the mitochondrial processing protease (MPP)	5,55	50897	0	0	0,00	0	0	0,00	1	1	10,13	1	1	10,12	0	0	0,00	0	0	0,00	1	1	10,14	1	1	10,13
254571955	C4R5S2	UBA1	Ubiquitin activating enzyme (E1), involved in ubiquitin-mediated protein degradation	4,83	115061	0	0	0,00	0	0	0,00	0	0	0,00	0	0	0,00	1	1	10,16	2	1	10,16	0	0	0,00	0	0	0,00
254571957	C4R5S3	MOG1	hypothetical protein	4,64	22311	0	0	0,00	0	0	0,00	0	0	0,00	0	0	0,00	0	0	0,00	0	0	0,00	2	1	10,15	0	0	0,00
254571977	C4R5T3	PAS_chr3_0865	Protein with a potential role in actin cytoskeletal organization	7,16	92668	0	0	0,00	0	0	0,00	0	0	0,00	0	0	0,00	0	0	0,00	0	0	0,00	0	0	0,00	1	1	10,11
254571985	C4R5T7	PP7435_Chr3-0310	hypothetical protein	9,56	22126	2	3	25,22	1	2	20,21	0	0	0,00	1	1	10,11	0	0	0,00	0	0	0,00	0	0	0,00	0	0	0,00
254572005	C4R5U7	PAS_chr3_0876	S-adenosylmethionine synthetase	6,07	42331	1	1	10,13	0	0	0,00	0	0	0,00	1	1	10,16	0	0	0,00	0	0	0,00	2	3	30,19	2	4	30,20
254572033	C4R5W1	SAHH	S-adenosyl-L-homocysteine hydrolase	5,33	48631	1	1	10,12	2	1	10,14	0	0	0,00	0	0	0,00	0	0	0,00	0	0	0,00	2	3	25,16	2	2	15,15
254572047	C4R5W8	PP7435_Chr3-0276	hypothetical protein	6,27	51347	0	0	0,00	0	0	0,00	1	1	10,13	0	0	0,00	1	1	10,14	0	0	0,00	0	0	0,00	0	0	0,00
254572049	C4R5W9	PP7435_Chr3-0275	Non-ATPase base subunit of the 19S regulatory particle of the 26S proteasome	4,51	102000	0	0	0,00	0	0	0,00	0	0	0,00	1	1	10,20	2	3	20,19	0	0	0,00	2	5	25,20	1	1	10,21
254572093	C4R5Z1	PP7435_Chr3-0255	ATPase of the ATP-binding cassette (ABC) family involved in 40S and 60S ribosome biogenesis	6,12	67327	0	0	0,00	0	0	0,00	2	3	15,21	0	0	0,00	0	0	0,00	0	0	0,00	1	1	10,21	1	1	10,18
254572117	C4R603	PP7435_Chr3-0242	Protein required for beta-1,6 glucan biosynthesis	4,83	165903	0	0	0,00	0	0	0,00	0	0	0,00	0	0	0,00	0	0	0,00	0	0	0,00	1	1	10,17	0	0	0,00
254572123	C4R606	FDH1	NAD(+)-dependent formate dehydrogenase, may protect cells from exogenous formate	6,70	40267	2	2	15,14	0	0	0,00	0	0	0,00	0	0	0,00	0	0	0,00	0	0	0,00	2	5	15,17	0	0	0,00
254572127	C4R608	PAS_chr3_0933	Ubiquitin-specific protease that specifically disassembles unanchored ubiquitin chains	4,79	90351	0	0	0,00	0	0	0,00	0	0	0,00	0	0	0,00	0	0	0,00	1	1	10,14	0	0	0,00	0	0	0,00
254572135	C4R612	EHD3	3-hydroxyisobutyryl-CoA hydrolase, member of a family of enoyl-CoA hydratase/isomerases	5,30	56389	0	0	0,00	0	0	0,00	2	3	10,18	0	0	0,00	2	4	25,17	0	0	0,00	2	5	10,19	2	5	15,19
254572139	C4R614	PP7435_Chr3-0232	Ser-Thr protein kinase, member (with Ark1p and Prk1p) of the Ark kinase family	5,25	72307	0	0	0,00	0	0	0,00	0	0	0,00	0	0	0,00	0	0	0,00	0	0	0,00	1	1	10,11	0	0	0,00
254572145	C4R617	PP7435_Chr3-0229	Coproporphyrinogen III oxidase, an oxygen requiring enzyme	5,80	37282	2	68	30,26	2	39	30,25	2	3	15,21	1	2	20,17	2	10	35,24	2	5	20,20	2	6	10,26	2	10	20,28
254572159	C4R624	kars	Lysyl-tRNA synthetase	5,49	67464	0	0	0,00	0	0	0,00	1	1	10,13	1	1	10,13	0	0	0,00	0	0	0,00	0	0	0,00	0	0	0,00
254572163	C4R626	PAS_chr3_0951	Triose phosphate isomerase, abundant glycolytic enzyme	5,66	27074	2	11	15,21	2	13	10,21	2	5	10,21	0	0	0,00	2	5	10,21	1	1	10,19	2	4	20,20	2	6	10,21
254572175	C4R632	CP51	Lanosterol 14-alpha-demethylase, catalyzes the C-14 demethylation of lanosterol	7,70	58805	1	1	10,17	1	1	10,13	0	0	0,00	0	0	0,00	0	0	0,00	0	0	0,00	0	0	0,00	0	0	0,00
254572199	C4R644	PAS_chr3_1244	hypothetical protein	6,03	46455	0	0	0,00	0	0	0,00	1	1	10,13	0	0	0,00	0	0	0,00	0	0	0,00	0	0	0,00	1	1	10,12
254572227	C4R658	PP7435_Chr3-0189	Putative serine/threonine protein kinase with unknown cellular role	7,18	38823	0	0	0,00	0	0	0,00	1	1	10,14	1	1	10,14	0	0	0,00	0	0	0,00	0	0	0,00	0	0	0,00
254572233	C4R661	PP7435_Chr3-0186	Protein of unknown function, deletion causes hypersensitivity to the K1 killer toxin	4,08	35210	2	1	10,19	2	1	10,20	2	1	10,21	0	0	0,00	0	0	0,00	0	0	0,00	0	0	0,00	0	0	0,00
254572239	C4R664	ALD5	Mitochondrial aldehyde dehydrogenase	5,37	53935	2	5	30,15	2	7	25,15	1	1	10,14	0	0	0,00	2	4	30,17	1	1	10,13	2	6	35,20	2	2	15,17
254572265	C4R677	TUP1	General repressor of transcription, forms complex with Cyc8p	5,95	65814	1	1	10,13	0	0	0,00	0	0	0,00	0	0	0,00	0	0	0,00	0	0	0,00	2	1	10,13	2	1	10,15
254572283	C4R686	GLR1	Cytosolic and mitochondrial glutathione oxidoreductase	8,28	50254	0	0	0,00	0	0	0,00	0	0	0,00	0	0	0,00	2	2	10,22	0	0	0,00	0	0	0,00	0	0	0,00
254572309	C4R699	PP7435_Chr3-0146	hypothetical protein	9,10	16100	1	1	10,14	1	1	10,15	0	0	0,00	0	0	0,00	0	0	0,00	0	0	0,00	0	0	0,00	0	0	0,00
254572311	C4R6A0	gltB	NAD(+)-dependent glutamate synthase (GOGAT)	5,99	236697	1	1	10,17	2	2	15,17	0	0	0,00	0	0	0,00	1	2	20,19	0	0	0,00	2	3	25,20	0	0	0,00
254572321	C4R6A5	FLD1	S-(hydroxymethyl)glutathione dehydrogenase	6,07	40515	2	3	10,24	2	2	10,23	2	1	10,24	0	0	0,00	2	6	20,27	1	1	10,11	2	5	20,28	2	4	15,24
254572343	C4R6B6	PP7435_Chr3-0128	Subunit of the Nup84p subcomplex of the nuclear pore complex (NPC)	4,40	87569	0	0	0,00	1	1	10,15	0	0	0,00	0	0	0,00	0	0	0,00	0	0	0,00	0	0	0,00	0	0	0,00
254572345	C4R6B7	PP7435_Chr3-0127	Proteolipid subunit of the vacuolar H(+)-ATPase V0 sector	7,97	16277	0	0	0,00	0	0	0,00	0	0	0,00	0	0	0,00	1	1	10,13	0	0	0,00	0	0	0,00	0	0	0,00
254572349	C4R6B9	dis3	Catalytic component of the exosome, involved in RNA processing and degradation	6,31	108670	0	0	0,00	0	0	0,00	1	1	10,17	0	0	0,00	1	1	10,12	0	0	0,00	0	0	0,00	0	0	0,00
254572367	C4R6C8	PP7435_Chr3-0116	Putative protein of unknown function	4,91	40694	0	0	0,00	2	1	10,16	2	3	10,17	2	2	10,17	2	3	15,16	0	0	0,00	0	0	0,00	0	0	0,00
254572371	C4R6D0	PAS_chr3_1054	Translation initiation factor eIF-4B, has RNA annealing activity	5,50	43478	2	1	10,12	0	0	0,00	0	0	0,00	0	0	0,00	0	0	0,00	0	0	0,00	1	1	10,14	0	0	0,00
254572403	C4R6E6	RPN6	Essential, non-ATPase regulatory subunit of the 26S proteasome lid	5,67	49776	0	0	0,00	0	0	0,00	0	0	0,00	0	0	0,00	0	0	0,00	0	0	0,00	1	1	10,17	0	0	0,00
254572423	C4R6F6	PP7435_Chr3-0086	Component of the U4/U6-U5 snRNP complex, involved in the second catalytic step of splicing	9,17	280688	0	0	0,00	0	0	0,00	0	0	0,00	0	0	0,00	2	1	10,16	0	0	0,00	0	0	0,00	0	0	0,00
254572445	C4R6G7	PAS_chr3_1086	Putative protein of unknown function	4,95	124441	0	0	0,00	0	0	0,00	1	1	10,15	1	1	10,15	0	0	0,00	0	0	0,00	1	1	10,14	0	0	0,00
254572447	C4R6G8	36982	Vacuolar aspartyl protease (proteinase A)	4,49	44278	0	0	0,00	0	0	0,00	2	1	10,15	0	0	0,00	0	0	0,00	2	5	35,18	2	4	30,22	2	12	70,25
254572455	C4R6H2	PP7435_Chr3-0068	Protein kinase implicated in activation of the plasma membrane H(+)-ATPase Pma1p	8,30	66146	0	0	0,00	0	0	0,00	0	0	0,00	0	0	0,00	0	0	0,00	0	0	0,00	2	1	10,18	0	0	0,00
254572461	C4R6H5	PAS_chr3_1095	Protein of unknown function, copurifies with late Golgi vesicles containing the v-SNARE Tlg2p	4,78	64845	1	1	10,20	0	0	0,00	2	1	10,22	1	1	10,20	2	1	10,24	1	1	10,15	1	1	10,17	0	0	0,00
254572491	C4R6J0	GTS1	Protein that localizes to the nucleus and is involved in transcription regulation	7,69	30924	0	0	0,00	0	0	0,00	1	1	10,12	0	0	0,00	0	0	0,00	0	0	0,00	0	0	0,00	0	0	0,00
254572497	C4R6J3	PP7435_Chr3-0050	DNA ligase found in the nucleus and mitochondria	6,79	83874	0	0	0,00	0	0	0,00	0	0	0,00	0	0	0,00	0	0	0,00	0	0	0,00	1	1	10,18	0	0	0,00
254572501	C4R6J5	NTF2	Nuclear envelope protein, interacts with GDP-bound Gsp1p and with proteins of the nuclear pore	4,40	14432	1	2	10,13	2	2	10,13	1	1	10,12	2	1	10,17	0	0	0,00	2	2	10,19	1	1	10,16	2	2	15,19
254572503	C4R6J6	PMI1	Mannose-6-phosphate isomerase, catalyzes the interconversion of fructose-6-P and mannose-6-P	5,39	49626	2	2	22,16	2	2	19,15	2	2	19,15	1	1	10,12	2	1	14,15	2	2	10,14	2	2	15,17	0	0	0,00
254572523	C4R6K6	PAS_chr3_1124	Protein of unknown function, predicted to contain a single transmembrane domain	6,37	74203	0	0	0,00	0	0	0,00	0	0	0,00	0	0	0,00	0	0	0,00	1	1	10,15	0	0	0,00	0	0	0,00
254572531	C4R362	PP7435_Chr3-0034	Alpha 5 subunit of the 20S proteasome involved in ubiquitin-dependent catabolism	4,56	27955	0	0	0,00	0	0	0,00	1	1	10,15	0	0	0,00	0	0	0,00	1	1	10,15	0	0	0,00	2	1	10,16
254572535	C4R364	SKP1	hypothetical protein	4,30	21815	0	0	0,00	1	1	10,14	0	0	0,00	0	0	0,00	2	1	10,15	0	0	0,00	0	0	0,00	0	0	0,00
254572539	C4R366	PAS_chr3_1132	Kynurenine aminotransferase, catalyzes formation of kynurenic acid from kynurenine	6,73	49206	2	4	20,21	2	3	20,20	2	2	10,19	0	0	0,00	2	2	10,21	1	1	10,13	0	0	0,00	0	0	0,00
254572553	C4R373	APT1	Adenine phosphoribosyltransferase, catalyzes the formation of AMP	5,11	19790	0	0	0,00	0	0	0,00	0	0	0,00	0	0	0,00	0	0	0,00	0	0	0,00	2	2	15,12	0	0	0,00
254572571	C4R382	PP7435_Chr3-0015	hypothetical protein	7,14	97746	1	1	10,14	0	0	0,00	0	0	0,00	0	0	0,00	0	0	0,00	0	0	0,00	0	0	0,00	0	0	0,00
254572579	C4R386	PP7435_Chr3-0011	hypothetical protein	10,50	17126	1	1	10,18	1	1	10,15	0	0	0,00	0	0	0,00	0	0	0,00	0	0	0,00	0	0	0,00	0	0	0,00
254572585	C4R389	PP7435_Chr3-0008	60S ribosomal protein L14	4,62	14923	2	2	15,15	2	3	15,15	2	1	10,16	1	1	10,13	0	0	0,00	2	2	10,16	2	2	10,17	2	1	10,14
254572597	C4R395	PAS_c131_0017	hypothetical protein	5,85	53642	0	0	0,00	0	0	0,00	0	0	0,00	0	0	0,00	2	2	10,15	0	0	0,00	2	1	10,17	0	0	0,00
254572599	C4R396	PAS_c131_0021	vacuolar ATP synthase subunit B	5,12	55363	0	0	0,00	0	0	0,00	1	1	14,15	0	0	0,00	2	2	20,20	0	0	0,00	0	0	0,00	2	1	10,15
254572638	C4R6M4	PP7435_Chr4-0995	Ubiquitin-specific protease that may play a role in ubiquitin precursor processing	4,87	140746	0	0	0,00	0	0	0,00	0	0	0,00	0	0	0,00	0	0	0,00	0	0	0,00	2	2	20,26	0	0	0,00
254572656	C4R6N3	PAS_chr4_0032	Alpha subunit of the translation initiation factor eIF2B	5,77	34065	0	0	0,00	0	0	0,00	0	0	0,00	0	0	0,00	1	1	10,12	0	0	0,00	0	0	0,00	0	0	0,00
254572664	C4R6N7	PAS_chr4_0037	Protein kinase involved in the response to oxidative and osmotic stress	5,82	58112	1	1	10,16	0	0	0,00	0	0	0,00	0	0	0,00	0	0	0,00	0	0	0,00	0	0	0,00	0	0	0,00
254572668	C4R6N9	CEF3	Translational elongation factor 3, stimulates the binding of aminoacyl-tRNA (AA-tRNA) to ribosomes	5,47	116532	0	0	0,00	0	0	0,00	2	6	20,19	2	2	10,16	2	5	24,18	1	1	10,12	2	14	65,21	2	3	34,18
254572672	C4R6P1	PP7435_Chr4-0977	Protein of unknown function	4,61	25625	0	0	0,00	0	0	0,00	0	0	0,00	0	0	0,00	0	0	0,00	0	0	0,00	2	2	20,23	2	2	20,20
254572694	C4R6Q2	YMC1	Mitochondrial protein, putative inner membrane transporter	9,91	31212	0	0	0,00	0	0	0,00	1	1	10,13	0	0	0,00	0	0	0,00	0	0	0,00	0	0	0,00	0	0	0,00
254572724	C4R6R7	PAS_chr4_0066	Alpha 1 subunit of the 20S core complex of the 26S proteasome	5,79	27790	0	0	0,00	0	0	0,00	0	0	0,00	0	0	0,00	1	1	10,12	0	0	0,00	0	0	0,00	0	0	0,00
254572730	C4R6S0	PP7435_Chr4-0948	Nucleosome assembly factor, involved in chromatin assembly and disassembly	3,52	36935	0	0	0,00	0	0	0,00	0	0	0,00	0	0	0,00	0	0	0,00	0	0	0,00	2	1	10,20	0	0	0,00
254572744	C4R6S7	PP7435_Chr4-0942	One of two (see also PSK1) PAS domain containing S/T protein kinases	6,24	136632	0	0	0,00	0	0	0,00	0	0	0,00	0	0	0,00	0	0	0,00	0	0	0,00	1	1	10,14	0	0	0,00
254572756	C4R6T3	SQT1	Essential protein involved in a late step of 60S ribosomal subunit assembly or modification	4,06	46543	0	0	0,00	0	0	0,00	0	0	0,00	0	0	0,00	0	0	0,00	0	0	0,00	0	0	0,00	1	1	14,11
254572790	C4R6V0	PP7435_Chr4-0917	hypothetical protein	5,49	44857	0	0	0,00	1	1	10,13	1	1	10,14	0	0	0,00	0	0	0,00	0	0	0,00	1	1	10,15	0	0	0,00
254572796	C4R6V3	CAM1	Nuclear protein required for transcription of MXR1	6,09	47062	2	2	10,15	2	3	20,15	2	8	45,16	2	4	10,14	2	11	35,17	2	2	10,13	2	5	30,16	2	3	29,16
254572802	C4R6V6	PP7435_Chr4-0911	Putative GTPase that associates with free 60S ribosomal subunits in the nucleolus	9,49	72861	0	0	0,00	0	0	0,00	1	1	10,14	0	0	0,00	0	0	0,00	0	0	0,00	0	0	0,00	0	0	0,00
254572808	C4R6V9	PP7435_Chr4-0908	hypothetical protein	5,03	64730	0	0	0,00	0	0	0,00	0	0	0,00	0	0	0,00	0	0	0,00	0	0	0,00	1	1	10,18	0	0	0,00
254572814	C4R6W2	GLY1	Threonine aldolase	6,12	41315	2	2	10,17	2	2	10,18	2	4	10,22	0	0	0,00	2	5	15,23	0	0	0,00	2	2	10,19	2	1	10,15
254572822	C4R6W6	PP7435_Chr4-0902	Minor isoform of tropomyosin, binds to and stabilizes actin cables and filaments	4,60	19018	2	4	15,15	2	2	10,14	2	3	19,17	0	0	0,00	2	5	19,19	0	0	0,00	2	1	10,18	2	4	25,17
254572834	C4R6X2	ALI1	hypothetical protein	8,60	33100	2	1	10,16	0	0	0,00	0	0	0,00	0	0	0,00	0	0	0,00	0	0	0,00	1	2	20,17	0	0	0,00
254572836	C4R6X3	CCT2	Subunit beta of the cytosolic chaperonin Cct ring complex, related to Tcp1p	5,63	56774	0	0	0,00	0	0	0,00	0	0	0,00	0	0	0,00	0	0	0,00	0	0	0,00	1	1	10,16	0	0	0,00
254572842	C4R6X6	PMS1	ATP-binding protein required for mismatch repair in mitosis and meiosis	5,12	101080	0	0	0,00	0	0	0,00	0	0	0,00	0	0	0,00	1	1	10,11	0	0	0,00	0	0	0,00	0	0	0,00
254572856	C4R6Y3	RPL6	60S ribosomal protein L6	8,96	18577	2	6	15,23	2	7	20,24	2	8	20,23	1	3	10,22	2	4	15,23	2	2	15,21	2	5	40,22	2	3	20,21
254572866	C4R6Y8	PAS_chr4_0137	Putative tubulin tyrosine ligase associated with P-bodies	5,26	83519	0	0	0,00	0	0	0,00	1	1	18,19	0	0	0,00	2	3	15,17	0	0	0,00	1	2	10,19	0	0	0,00
254572868	C4R6Y9	CPA1	Small subunit of carbamoyl phosphate synthetase	6,53	46379	2	3	10,16	2	4	10,17	2	3	10,18	0	0	0,00	2	2	10,18	2	4	10,18	2	2	10,16	2	3	10,18
254572872	C4R6Z1	PP7435_Chr4-0875	ATP-dependent DEAD (Asp-Glu-Ala-Asp)-box RNA helicase	7,35	66379	0	0	0,00	0	0	0,00	2	2	10,17	1	1	10,15	2	3	10,19	0	0	0,00	2	3	20,16	2	3	20,17
254572884	C4R6Z7	PP7435_Chr4-0869	Aromatic aminotransferase II	5,53	51671	1	1	10,14	2	2	15,16	0	0	0,00	0	0	0,00	0	0	0,00	1	1	10,16	2	4	20,21	2	3	20,18
254572890	C4R700	HOM3	Aspartate kinase (L-aspartate 4-P-transferase)	5,99	57017	0	0	0,00	0	0	0,00	0	0	0,00	0	0	0,00	0	0	0,00	0	0	0,00	1	1	18,17	0	0	0,00
254572896	C4R703	PLB3	Phospholipase B (lysophospholipase) involved in phospholipid metabolism	3,95	69609	0	0	0,00	0	0	0,00	0	0	0,00	0	0	0,00	0	0	0,00	0	0	0,00	0	0	0,00	2	2	15,14
254572906	C4R708	PAS_chr4_0158	Tetradecameric mitochondrial chaperonin	4,90	60138	2	9	45,22	2	11	55,23	2	34	178,28	2	16	85,24	2	21	145,26	2	49	195,26	2	83	230,30	2	84	237,27
254572920	C4R715	PAS_chr4_0164	hypothetical protein	10,70	6652	0	0	0,00	0	0	0,00	0	0	0,00	1	1	10,11	0	0	0,00	2	1	10,15	0	0	0,00	0	0	0,00
254572936	C4R723	uca	Urea amidolyase, contains both urea carboxylase and allophanate hydrolase activities	5,42	201006	0	0	0,00	0	0	0,00	0	0	0,00	0	0	0,00	2	4	25,18	0	0	0,00	2	4	30,19	0	0	0,00
254572958	C4R734	PP7435_Chr4-0828	BAR domain-containing protein that localizes to both early and late Golgi vesicles	4,77	37725	2	4	15,23	2	4	20,23	2	3	20,26	1	1	10,10	2	3	20,24	2	2	20,16	2	3	10,26	2	4	20,24
254572962	C4R736	NSR1	Nucleolar protein that binds nuclear localization sequences	4,88	38774	2	9	25,19	2	7	20,19	2	5	20,18	0	0	0,00	2	3	20,19	1	1	10,14	2	5	20,21	2	2	15,16
254572972	C4R741	PAS_chr4_0191	Malate synthase	6,17	65632	2	2	10,21	2	3	15,22	0	0	0,00	0	0	0,00	2	1	10,19	1	1	10,14	2	1	10,20	0	0	0,00
254572974	C4R742	FMO2	Flavin-containing monooxygenase	5,20	57756	0	0	0,00	1	1	10,14	0	0	0,00	0	0	0,00	0	0	0,00	1	1	10,14	0	0	0,00	0	0	0,00
254572976	C4R743	PP7435_Chr4-0817	hypothetical protein	5,07	33704	2	1	10,15	0	0	0,00	0	0	0,00	0	0	0,00	0	0	0,00	0	0	0,00	0	0	0,00	1	1	10,16
254572988	C4R749	PAS_chr4_0198	C-8 sterol isomerase	5,15	23254	0	0	0,00	0	0	0,00	0	0	0,00	0	0	0,00	0	0	0,00	1	1	10,14	0	0	0,00	0	0	0,00
254573010	C4R760	PAS_chr4_0210	Major ADP/ATP carrier of the mitochondrial inner membrane	10,15	33190	0	0	0,00	0	0	0,00	0	0	0,00	0	0	0,00	0	0	0,00	0	0	0,00	2	2	10,11	0	0	0,00

254573014	C4R762	PP7435_Chr4-0798	40S ribosomal protein S7	9,65	21302	0	0	0,00	0	0	0,00	0	0	0,00	0	0	0,00	1	1	10,12	0	0	0,00	0	0	0,00	0	0	0,00
254573018	C4R764	PAS_chr4_0213	Ribose-5-phosphate ketol-isomerase	7,21	27216	1	1	10,11	2	1	10,14	0	0	0,00	0	0	0,00	0	0	0,00	0	0	0,00	0	0	0,00	0	0	0,00
254573030	C4R770	PP7435_Chr4-0791	Mitochondrial type 2C protein phosphatase involved in regulation of pyruvate dehydrogenase activity	9,77	65144	0	0	0,00	0	0	0,00	0	0	0,00	0	0	0,00	0	0	0,00	0	0	0,00	1	1	10,15	0	0	0,00
254573036	C4R773	YVC1	Vacuolar cation channel	5,11	73822	1	1	10,13	0	0	0,00	0	0	0,00	0	0	0,00	0	0	0,00	0	0	0,00	0	0	0,00	0	0	0,00
254573046	C4R778	PP7435_Chr4-0783	Gamma subunit of coatomer, a heptameric protein complex that together with Arf1p forms the COPI coat	4,99	100825	0	0	0,00	0	0	0,00	0	0	0,00	0	0	0,00	1	1	10,13	0	0	0,00	2	6	20,19	1	1	10,13
254573048	C4R779	PP7435_Chr4-0782	hypothetical protein	4,88	39768	1	1	10,16	2	1	10,15	2	2	10,19	1	1	10,17	2	3	20,20	1	1	10,13	2	1	10,21	2	1	10,20
254573056	C4R783	PAS_chr4_0228	Acetolactate synthase, catalyses the first common step in isoleucine and valine biosynthesis	6,39	72969	0	0	0,00	0	0	0,00	2	6	20,19	1	2	20,14	1	1	10,15	1	1	10,15	2	4	20,18	1	1	10,14
254573058	C4R784	TRF4	Catalytic subunit of TRAMP (Trf4/Pap2p-Mtr4p-Air1p/2p)	5,52	68096	0	0	0,00	0	0	0,00	0	0	0,00	0	0	0,00	1	1	10,14	0	0	0,00	0	0	0,00	0	0	0,00
254573080	C4R795	PP7435_Chr4-0766	Putative ATPase of the AAA family	9,80	79515	0	0	0,00	0	0	0,00	0	0	0,00	0	0	0,00	0	0	0,00	0	0	0,00	1	1	10,17	0	0	0,00
254573084	C4R797	NUA3	Protein of unknown function	5,23	70229	1	1	10,15	0	0	0,00	0	0	0,00	0	0	0,00	0	0	0,00	0	0	0,00	0	0	0,00	0	0	0,00
254573092	C4R7A1	RPS4	40S ribosomal protein S4	9,61	31713	2	2	10,15	2	2	10,15	2	1	10,12	1	1	10,12	1	1	10,12	0	0	0,00	2	2	20,15	0	0	0,00
254573094	C4R7A2	PP7435_Chr4-0758	hypothetical protein	9,30	30699	0	0	0,00	0	0	0,00	0	0	0,00	0	0	0,00	0	0	0,00	0	0	0,00	0	0	0,00	1	1	10,13
254573098	C4R7A4	PAS_chr4_0248	Mitochondrial branched-chain amino acid aminotransferase, homolog of murine ECA39	5,76	44670	2	5	10,18	2	6	15,15	2	4	20,19	2	1	10,15	2	4	30,18	0	0	0,00	2	4	10,20	2	3	10,19
254573100	C4R7A5	PCC1	Proposed transcription factor	4,37	10133	0	0	0,00	0	0	0,00	0	0	0,00	0	0	0,00	0	0	0,00	0	0	0,00	1	1	10,15	0	0	0,00
254573102	C4R7A6	PAS_chr4_0250	DEAD-box protein required for efficient splicing of mitochondrial Group I and II introns	9,91	76137	0	0	0,00	0	0	0,00	0	0	0,00	0	0	0,00	0	0	0,00	0	0	0,00	1	1	10,13	0	0	0,00
254573108	C4R7A9	PAS_chr4_0253	Mitochondrial ribosome recycling factor	10,21	27923	2	2	20,18	1	2	20,18	0	0	0,00	0	0	0,00	0	0	0,00	0	0	0,00	0	0	0,00	0	0	0,00
254573112	C4R7B1	PAS_chr4_0255	hypothetical protein	5,81	13029	0	0	0,00	0	0	0,00	0	0	0,00	0	0	0,00	0	0	0,00	0	0	0,00	1	1	10,13	0	0	0,00
254573140	C4R7C5	PP7435_Chr4-0736	Similar to 6-phosphofructo-2-kinase/fructose-2,6-bisphosphatase enzymes	7,10	57800	0	0	0,00	1	1	10,15	1	1	10,14	0	0	0,00	2	1	10,15	0	0	0,00	2	1	10,15	0	0	0,00
254573148	C4R7C9	MSP1	Mitochondrial protein involved in sorting of proteins in the mitochondria	7,93	39538	0	0	0,00	0	0	0,00	0	0	0,00	0	0	0,00	0	0	0,00	0	0	0,00	2	1	10,26	0	0	0,00
254573156	C4R7D3	PP7435_Chr4-0728	Essential nucleolar protein involved in the early steps of 35S rRNA processing	9,00	28949	1	1	10,15	0	0	0,00	2	1	10,15	0	0	0,00	1	1	10,16	0	0	0,00	0	0	0,00	0	0	0,00
254573164	C4R7D7	PP7435_Chr4-0723	Protein involved in pre-rRNA processing, 18S rRNA synthesis, and snoRNA synthesis	8,43	57918	0	0	0,00	0	0	0,00	0	0	0,00	0	0	0,00	1	1	10,13	0	0	0,00	0	0	0,00	1	1	10,14
254573174	C4R7E2	PP7435_Chr4-0717	hypothetical protein	9,57	27571	2	2	20,17	0	0	0,00	0	0	0,00	0	0	0,00	2	2	10,19	0	0	0,00	1	1	10,21	2	3	20,16
254573180	C4R7E5	TRX2	Cytoplasmic thioredoxin isoenzyme of the thioredoxin system	4,70	11252	2	2	15,18	0	0	0,00	0	0	0,00	0	0	0,00	0	0	0,00	0	0	0,00	0	0	0,00	0	0	0,00
254573188	C4R7E9	DIP5	Dicarboxylic amino acid permease	8,94	65309	0	0	0,00	1	1	18,13	0	0	0,00	0	0	0,00	0	0	0,00	0	0	0,00	0	0	0,00	0	0	0,00
254573190	C4R7F0	PBP1	Protein interacting with poly(A)-binding protein Pab1p	8,95	73559	0	0	0,00	0	0	0,00	0	0	0,00	0	0	0,00	0	0	0,00	0	0	0,00	2	1	10,15	0	0	0,00
254573192	C4R7F1	PAS_chr4_0289	hypothetical protein	4,89	12714	0	0	0,00	0	0	0,00	2	1	10,19	2	1	10,18	1	1	10,15	2	2	10,21	2	1	10,16	2	1	10,18
254573200	C4R7F5	PP7435_Chr4-0706	Component of the septin ring of the mother-bud neck that is required for cytokinesis	5,93	43427	1	1	10,13	1	1	10,13	2	2	15,16	0	0	0,00	0	0	0,00	0	0	0,00	2	2	15,17	0	0	0,00
254573216	C4R7G3	pam2	One of six ATPases of the 19S regulatory particle of the 26S proteasome	4,88	46758	0	0	0,00	0	0	0,00	0	0	0,00	0	0	0,00	1	1	10,14	1	1	10,11	2	2	10,16	0	0	0,00
254573242	C4R7H6	PAS_chr4_0311	2-enoyl thioester reductase, member of the medium chain dehydrogenase/reductase family	7,91	41227	0	0	0,00	0	0	0,00	0	0	0,00	0	0	0,00	2	3	25,19	0	0	0,00	2	4	15,25	0	0	0,00
254573248	C4R7H9	CLA4	Member of the PAK family of serine/threonine protein kinases with similarity to Ste20p and Cla4p	9,21	84791	0	0	0,00	0	0	0,00	0	0	0,00	0	0	0,00	0	0	0,00	0	0	0,00	1	1	10,18	0	0	0,00
254573252	C4R7I1	PP7435_Chr4-0680	hypothetical protein	5,21	52670	0	0	0,00	0	0	0,00	0	0	0,00	0	0	0,00	0	0	0,00	0	0	0,00	2	1	10,13	0	0	0,00
254573266	C4R7I8	MYH4	hypothetical protein	4,70	92679	2	1	10,20	2	1	10,19	0	0	0,00	0	0	0,00	0	0	0,00	0	0	0,00	0	0	0,00	0	0	0,00
254573284	C4R7J7	PP7435_Chr4-0665	Methionine and cysteine synthase (O-acetyl homoserine-O-acetyl serine sulfhydrylase)	5,60	48148	1	1	10,12	0	0	0,00	0	0	0,00	0	0	0,00	0	0	0,00	0	0	0,00	0	0	0,00	0	0	0,00
254573296	C4R7K3	PP7435_Chr4-0659	Putative dihydrokaempferol 4-reductase	5,41	39527	0	0	0,00	0	0	0,00	1	3	20,18	1	1	18,15	1	1	10,16	2	5	38,20	2	9	42,28	2	5	42,22
254573310	C4R7L0	ATH1	Acid trehalase required for utilization of extracellular trehalose	4,64	113900	0	0	0,00	0	0	0,00	0	0	0,00	0	0	0,00	0	0	0,00	0	0	0,00	1	1	10,12	0	0	0,00
254573316	C4R7L3	SCD6	Protein containing an Lsm domain, may bind RNA and have a role in RNA processing	9,38	34613	2	3	15,24	1	1	10,23	2	1	10,17	0	0	0,00	2	3	10,21	0	0	0,00	2	2	10,18	1	1	10,17
254573318	C4R7L4	NAT3	Catalytic subunit of the NatB N-terminal acetyltransferase	5,90	22978	2	1	10,11	0	0	0,00	0	0	0,00	0	0	0,00	0	0	0,00	0	0	0,00	0	0	0,00	0	0	0,00
254573334	C4R7M2	JAC1	Specialized J-protein that functions with Hsp70 in Fe-S cluster biogenesis in mitochondria	5,21	24820	2	2	10,22	2	2	10,21	2	2	10,25	0	0	0,00	2	3	10,26	0	0	0,00	2	3	10,28	2	2	10,27
254573338	C4R7M4	PP7435_Chr4-0638	hypothetical protein	6,33	20762	0	0	0,00	0	0	0,00	0	0	0,00	1	1	10,12	0	0	0,00	2	2	10,18	2	2	15,15	0	0	0,00
254573352	C4R7N1	PAS_chr4_0363	hypothetical protein	4,31	65574	1	1	10,13	0	0	0,00	0	0	0,00	0	0	0,00	0	0	0,00	0	0	0,00	0	0	0,00	0	0	0,00
254573364	C4R7N7	PP7435_Chr4-0625	hypothetical protein	5,94	36649	0	0	0,00	0	0	0,00	0	0	0,00	0	0	0,00	0	0	0,00	0	0	0,00	2	1	10,17	0	0	0,00
254573404	C4R7Q7	PP7435_Chr4-0605	hypothetical protein	5,27	41864	0	0	0,00	0	0	0,00	0	0	0,00	0	0	0,00	1	1	10,10	0	0	0,00	0	0	0,00	0	0	0,00
254573410	C4R7R0	PAS_chr4_0387	One of six ATPases of the 19S regulatory particle of the 26S proteasome	5,32	46807	0	0	0,00	0	0	0,00	0	0	0,00	0	0	0,00	0	0	0,00	0	0	0,00	1	1	10,14	0	0	0,00
254573414	C4R7R2	PAS_chr4_0388	Mitochondrial ribosomal protein of the large subunit	4,55	18798	0	0	0,00	0	0	0,00	0	0	0,00	0	0	0,00	0	0	0,00	0	0	0,00	1	1	10,12	0	0	0,00
254573420	C4R7R5	PP7435_Chr4-0596	Component of the TOM (translocase of outer membrane) complex	5,49	42730	0	0	0,00	0	0	0,00	0	0	0,00	0	0	0,00	0	0	0,00	0	0	0,00	2	1	10,14	0	0	0,00
254573426	C4R7R8	LARS	Cytosolic leucyl tRNA synthetase, ligates leucine to the appropriate tRNA	5,47	125315	0	0	0,00	0	0	0,00	1	1	10,12	0	0	0,00	0	0	0,00	0	0	0,00	1	1	10,13	0	0	0,00
254573438	C4R7S4	PP7435_Chr4-0586	Ferric reductase and cupric reductase	9,31	80400	0	0	0,00	0	0	0,00	0	0	0,00	0	0	0,00	0	0	0,00	1	1	10,12	0	0	0,00	0	0	0,00
254573452	C4R7T1	STF2	Protein of unknown function that associates with ribosomes	5,82	11563	2	3	20,15	2	3	20,15	0	0	0,00	0	0	0,00	0	0	0,00	0	0	0,00	0	0	0,00	0	0	0,00
254573462	C4R7T6	PP7435_Chr4-0573	60S acidic ribosomal protein P1	3,79	10990	2	1	10,19	2	1	10,17	1	1	10,13	0	0	0,00	2	1	10,14	0	0	0,00	0	0	0,00	0	0	0,00
254573464	C4R7T7	PP7435_Chr4-0572	60S ribosomal protein L13	11,17	22134	2	12	40,16	2	8	25,18	2	8	35,19	2	4	15,16	2	8	25,19	2	3	10,16	2	3	15,15	2	3	10,15
254573468	C4R7T9	SHM2	Cytosolic serine hydroxymethyltransferase	6,65	52031	0	0	0,00	0	0	0,00	0	0	0,00	0	0	0,00	0	0	0,00	2	2	20,17	1	1	10,16	2	1	10,18
254573470	C4R7U0	PAS_chr4_0416	Alanine:glyoxylate aminotransferase (AGT), catalyzes the synthesis of glycine from glyoxylate	8,34	45383	2	4	40,15	1	1	10,14	2	4	25,16	1	2	20,17	2	3	25,16	2	4	15,14	2	13	50,16	2	4	30,17
254573474	C4R7U2	HEM2	Delta-aminolevulinate dehydratase, a homo-octameric enzyme	6,14	37699	1	1	10,17	1	1	10,15	1	3	20,18	2	2	10,16	2	3	10,19	0	0	0,00	2	2	10,18	0	0	0,00
254573496	C4R7V3	PP7435_Chr4-0557	hypothetical protein	5,19	27180	0	0	0,00	0	0	0,00	0	0	0,00	0	0	0,00	0	0	0,00	1	1	10,12	2	1	10,20	0	0	0,00
254573500	C4R7V5	UBC1	Ubiquitin-conjugating enzyme that mediates selective degradation of short-lived and abnormal protein	5,14	26723	0	0	0,00	1	1	10,14	0	0	0,00	0	0	0,00	0	0	0,00	0	0	0,00	0	0	0,00	0	0	0,00
254573508	C4R7V9	PP7435_Chr4-0551	NADPH-dependent alpha-keto amide reductase	5,51	33673	0	0	0,00	0	0	0,00	2	2	15,18	2	1	10,20	2	2	15,18	1	1	10,20	2	4	30,18	2	2	15,18
254573510	C4R7W0	TIF35	Subunit of the core complex of translation initiation factor 3(eIF3)	8,87	31627	0	0	0,00	0	0	0,00	0	0	0,00	0	0	0,00	0	0	0,00	0	0	0,00	1	1	10,17	0	0	0,00
254573516	C4R7W3	PHA2	Prephenate dehydratase, catalyzes the conversion of prephanate to phenylpyruvate	5,09	34094	0	0	0,00	0	0	0,00	0	0	0,00	0	0	0,00	1	1	10,14	0	0	0,00	0	0	0,00	0	0	0,00
254573520	C4R7W5	PP7435_Chr4-0543	Heat shock protein with a zinc finger motif	9,67	18953	0	0	0,00	0	0	0,00	1	1	10,13	1	1	10,16	2	1	10,13	0	0	0,00	0	0	0,00	1	1	10,11
254573528	C4R7W9	PIM1	ATP-dependent Lon protease, involved in degradation of misfolded proteins in mitochondria	5,14	124155	0	0	0,00	0	0	0,00	0	0	0,00	0	0	0,00	0	0	0,00	0	0	0,00	2	2	13,31	0	0	0,00
254573542	C4R7X6	PAS_chr4_0448	Lsm (Like Sm) protein	6,09	11786	0	0	0,00	0	0	0,00	0	0	0,00	0	0	0,00	0	0	0,00	0	0	0,00	2	1	10,21	2	1	10,24
254573576	C4R7Z3	CFA1	hypothetical protein	6,60	56218	1	1	10,16	0	0	0,00	1	1	10,18	0	0	0,00	1	1	10,20	0	0	0,00	2	1	10,26	0	0	0,00
254573602	C4R806	PP7435_Chr4-0502	Essential protein involved in transcription regulation	5,58	49819	0	0	0,00	0	0	0,00	1	3	10,15	0	0	0,00	2	3	10,16	0	0	0,00	2	2	10,16	2	3	10,15
254573626	C4R818		Carbon source-responsive zinc-finger transcription factor	6,41	132627	0	0	0,00	0	0	0,00	0	0	0,00	0	0	0,00	0	0	0,00	0	0	0,00	1	1	10,14	0	0	0,00
254573636	C4R823	PAS_chr4_0493	Putative phosphomutase, contains a region homologous to the active site of phosphomutases	5,78	37913	1	2	20,17	1	1	10,13	0	0	0,00	0	0	0,00	0	0	0,00	0	0	0,00	2	3	20,17	1	1	10,15
254573640	C4R825	PP7435_Chr4-0482	hypothetical protein	9,90	54492	0	0	0,00	0	0	0,00	1	1	10,14	0	0	0,00	0	0	0,00	0	0	0,00	0	0	0,00	0	0	0,00
254573652	C4R831	LSB1	Protein that induces appearance of [PIN+] prion when overproduced	5,21	28761	0	0	0,00	0	0	0,00	0	0	0,00	0	0	0,00	0	0	0,00	0	0	0,00	1	1	10,14	0	0	0,00
254573664	C4R837	PAS_chr4_0506	Alpha 3 subunit of the 20S proteasome, the only nonessential 20S subunit	5,07	27931	0	0	0,00	0	0	0,00	0	0	0,00	0	0	0,00	0	0	0,00	0	0	0,00	1	1	10,17	2	1	10,17
254573688	C4R849	HGH1	Nonessential protein of unknown function	4,40	42144	0	0	0,00	0	0	0,00	0	0	0,00	0	0	0,00	0	0	0,00	0	0	0,00	1	1	10,13	0	0	0,00
254573694	C4R852	PAS_chr4_0522	Cytoplasmic tyrosyl-tRNA synthetase, class I aminoacyl-tRNA synthetase	6,79	45120	0	0	0,00	0	0	0,00	0	0	0,00	0	0	0,00	0	0	0,00	0	0	0,00	2	4	15,17	0	0	0,00
254573714	C4R862	PAS_chr4_0974	hypothetical protein	7,62	42396	2	2	10,12	2	2	10,12	1	1	10,14	1	1	10,15	1	1	10,14	1	1	10,14	2	3	15,14	2	4	10,15
254573726	C4R868	PAS_chr4_0533	Beta-tubulin	4,38	48774	0	0	0,00	0	0	0,00	1	1	10,14	0	0	0,00	0	0	0,00	0	0	0,00	2	13	10,23	1	1	10,15
254573734	C4R872	PAS_chr4_0537	Essential nuclear protein involved in early steps of ribosome biogenesis	4,21	56832	0	0	0,00	0	0	0,00	2	1	10,16	0	0	0,00	2	2	20,18	0	0	0,00	2	1	10,17	2	6	25,17
254573742	C4R876	PP7435_Chr4-0433	Protein that forms a complex with the Sit4p protein phosphatase and is required for its function	4,03	94657	0	0	0,00	0	0	0,00	0	0	0,00	0	0	0,00	1	1	10,14	0	0	0,00	0	0	0,00	0	0	0,00
254573756	C4R883	PAS_chr4_0548	Component of the septin ring of the mother-bud neck that is required for cytokinesis	5,61	54494	2	2	15,22	2	2	10,22	2	3	10,22	1	2	20,18	2	2	10,20	0	0	0,00	0	0	0,00	2	1	10,14
254573760	C4R885	PP7435_Chr4-0424	Putative protein of unknown function	5,48	36098	2	2	15,23	2	1	10,21	2	38	70,26	2	11	50,21	2	24	40,23	2	5	45,18	2	74	95,27	2	37	75,24
254573764	C4R887	HSA1	ATPase involved in protein folding and nuclear localization signal (NLS)-directed nuclear transport	4,74	69574	2	5	24,17	2	3	17,16	2	11	70,20	2	4	30,17	2	12	70,20	2	9	60,17	2	8	55,18	2	7	56,26
254573788	C4R899	CSL4	Subunit of the exosome, which is an essential complex present in both nucleus and cytoplasm	5,10	32208	0	0	0,00	1	1	10,14	0	0	0,00	0	0	0,00	0	0	0,00	0	0	0,00	0	0	0,00	0	0	0,00
254573806	C4R8A8	PP7435_Chr4-0399	hypothetical protein	5,55	50798	2	1	10,18	1	1	10,15	0	0	0,00	0	0	0,00	0	0	0,00	0	0	0,00	0	0	0,00	0	0	0,00
254573814	C4R8B2	NCE3	Carbonic anhydrase	4,94	32927	0	0	0,00	0	0	0,00	0	0	0,00	2	1	10,16	0	0	0,00	1	1	10,11	0	0	0,00	0	0	0,00
254573818	C4R8B4	IDH1	Subunit of mitochondrial NAD(+)-dependent isocitrate dehydrogenase	7,59	39554	0	0	0,00	0	0	0,00	0	0	0,00	0	0	0,00	1	1	10,14	0	0	0,00	0	0	0,00	0	0	0,00
254573840	C4R8C5	PP7435_Chr4-0380	hypothetical protein	4,65	87639	2	2	15,18	1	3	20,18	2	2	10,18	2	1	10,18	2	2	10,21	2	2	10,18	2	4	20,20	2	2	15,20
254573846	C4R8C8	PP7435_Chr4-0378	Glyoxylate reductase	6,02	38163	0	0	0,00	0	0	0,00	2	3	28,26	1	1	10,20	2	7	40,26	2	2	15,17	2	14	55,28	2	4	30,28
254573866	C4R8D8	PP7435_Chr4-0368	Acidic protein of the mitochondrial matrix involved in oxidative phosphorylation	4,43	28829	0	0	0,00	0	0	0,00	0	0	0,00	0	0	0,00	1	3	20,15	1	1	10,15	2	4	20,17	2	10	59,21
254573870	C4R8E0		Putative protein of unknown function	4,36	24748	0	0	0,00	0	0	0,00	0	0	0,00	0	0	0,00	0	0	0,00	1	1	10,12	0	0	0,00	0	0	0,00
254573878	C4R8E4	MKT1	Protein that forms a complex with Pbp1p	5,90	85336	0	0	0,00	0	0	0,00	0	0	0,00	0	0	0,00	0	0	0,00	0	0	0,00	1	1	10,17	1	1	10,13
254573886	C4R8E8	PP7435_Chr4-0357	Adenylosuccinate synthase	6,17	47720	2	3	15,17	2	2	15,16	2	10	40,19	2	2	10,15	2	11	35,19	2	4	30,16	2	10	30,21	2	7	25,18
254573892	C4R8F1	YRB2	hypothetical protein	5,18	40008	0	0	0,00	2	1	10,15	2	2	10,15	2	2	15,17	2	2	10,17	2	3	10,18	2	2	10,16	0	0	0,00
254573908	C4R8F9	EMI2	Non-essential protein of unknown function required for transcriptional induction	6,08	52325	2	3	20,15	2	3	20,14	2	2	10,15	0	0	0,00	2	2	15,14	0	0	0,00	2	7	40,18	2	6	35,17
254573914	C4R8G2	PP7435_Chr4-0342	Plasma membrane localized protein that protects membranes from desiccation	4,66	12431	0	0	0,00	0	0	0,00	0	0	0,00	0	0	0,00	0	0	0,00	0	0	0,00	0	0	0,00	1	1	10,17
254573922	C4R8G6	PP7435_Chr4-0337	hypothetical protein	9,57	45519	0	0	0,00	0	0	0,00	0	0	0,00	0	0	0,00	0	0	0,00	0	0	0,00	1	1	10,15	0	0	0,00
254573948	C4R8H9	PAS_chr4_0645	Acetylornithine aminotransferase	7,88	47484	2	1	10,14	2	1	10,14	2	1	10,15	1	3	20,15	2	1	10,15	1	1	10,15	0	0	0,00	2	3	20,16
254573986	C4R8J8	PRO2	Gamma-glutamyl phosphate reductase, catalyzes the second step in proline biosynthesis	5,33	49112	0	0	0,00	0	0	0,00	0	0	0,00	0	0	0,00	0	0	0,00	1	1	10,12	0	0	0,00	0	0	0,00
254573994	C4R8K2	PRE2	Beta 5 subunit of the 20S proteasome, responsible for the chymotryptic activity of the proteasome	5,55	31065	0	0	0,00	0	0	0,00	0	0	0,00	0	0	0,00	0	0	0,00	0	0	0,00	1	1	10,14	0	0	0,00
254574004	C4R8K7	PAS_chr4_0673	Putative protein of unknown function	6,62	29215	0	0	0,00	0	0	0,00	0	0	0,00	0	0	0,00	0	0	0,00	0	0	0,00	2	2	10,20	0	0	0,00
254574006	C4R8K8	VMA7	Subunit F of the eight-subunit V1 peripheral membrane domain of vacuolar H+-ATPase (V-ATPase)	4,78	13846	1	1	10,13	2	1	10,13	0	0	0,00	0	0	0,00	0	0	0,00	0	0	0,00	0	0	0,00	0	0	0,00
254574008	C4R8K9	CHS5	Component of the exomer complex, which also contains Csh6p, Bch1p, Bch2p, and Bud7p	4,25	66525	0	0	0,00	0	0	0,00	2	1	10,20	0	0	0,00	2	1	10,22	2	1	10,16	1	2	10,21	2	1	10,22
254574012	C4R8L1	UGA1	Gamma-aminobutyrate (GABA) transaminase (4-aminobutyrate aminotransferase)	5,85	51918	1	1	10,13	1	2	10,13	0	0	0,00	0	0	0,00	0	0	0,00	0	0	0,00	0	0	0,00	0	0	0,00
254574028	C4R8L9	CAR1	Arginase, responsible for arginine degradation	5,30	35120	0	0	0,00	1	1	10,13	2	6	10,14	2	7	20,15	2	7	30,16	2	8	20,15	2	10	35,23	2	4	10,14
254574030	C4R8M0	CPS1	Vacuolar carboxypeptidase yscS	4,98	65792	0	0	0,00	0	0	0,00	0	0	0,00	0	0	0,00	0	0	0,00	2	1	10,15	2	1	10,15	2	2	10,15
254574074	C4R8P2	PET112	Protein required for mitochondrial translation	6,52	55989	0	0	0,00	0	0	0,00	0	0	0,00	0	0	0,00	0	0	0,00	0	0	0,00	1	1	10,21	0	0	0,00
254574080	C4R8P5	ileS	isoleucine-tRNA synthetase	6,07	123289	0	0	0,00	0	0	0,00	0	0	0,00	0	0	0,00	1	1	10,16	0	0	0,00	0	0	0,00	0	0	0,00
254574100	C4R8Q5	RNA1	GTPase activating protein (GAP) for Gsp1p, involved in nuclear transport	4,37	46893	1	1	10,22	1	2	20,21	2	2	15,17	1	1	10,14	1	1	10,17	1	1	10,18	1	1	10,19	2	1	10,18
254574102	C4R8Q6	PP7435_Chr4-0243	Transcriptional coactivator	10,50	16461	1	2	10,14	0	0	0,00	1	2	20,15	0	0	0,00	1	1	10,11	0	0	0,00	0	0	0,00	1	1	10,12
254574120	C4R8R5	MAK5	Essential nucleolar protein, putative DEAD-box RNA helicase	8,65	85173	0	0	0,00	0	0	0,00	1	1	10,13	0	0	0,00	1	1	10,13	0	0	0,00	0	0	0,00	0	0	0,00
254574124	C4R8R7	PAS_chr4_0730	Inositol monophosphatase, involved in biosynthesis of inositol	4,55	26214	0	0	0,00	0	0	0,00	2	6	25,24	0	0	0,00	2	9	20,25	0	0	0,00	2	8	40,26	2	10	25,26
254574132	C4R8S1	SDH1	Flavoprotein subunit of succinate dehydrogenase (Sdh1p, Sdh2p, Sdh3p, Sdh4p)	6,08	70170	0	0	0,00	0	0	0,00	0	0	0,00	0	0	0,00	0	0	0,00	0	0	0,00	2	2	15,18	2	2	10,22
254574140	C4R8S5	PAS_chr4_0737	Mitochondrial NADH-cytochrome b5 reductase, involved in ergosterol biosynthesis	9,21	33553	2	1	10,14	0	0	0,00	2	1	10,15	1	1	10,11	2	2	15,14	1	1	10,12	2	3	25,18	2	3	25,16
254574142	C4R8S6	PP7435_Chr4-0222	Putative protein of unknown function	6,87	36870	0	0	0,00	0	0	0,00	0	0	0,00	0	0	0,00	0	0	0,00	1	1	10,14	0	0	0,00	0	0	0,00
254574144	C4R8S7	PP7435_Chr4-0221	Putative mitochondrial ribosomal protein, has similarity to E. coli ribosomal protein S2	4,22	22782	0	0	0,00	0	0	0,00	1	1	10,18	0	0	0,00	1	2	20,17	0	0	0,00	2	2	20,20	2	9	45,23
254574172	C4R8U1	SOU1	Peroxisomal 2,4-dienoyl-CoA reductase, auxiliary enzyme of fatty acid beta-oxidation	5,53	30800	0	0	0,00	0	0	0,00	0	0	0,00	0	0	0,00	0	0	0,00	0	0	0,00	1	3	20,17	2	2	10,18
254574176	C4R8U3	PAS_chr4_0755	hypothetical protein	4,86	31731	0	0	0,00	0	0	0,00	0	0	0,00	0	0	0,00	0	0	0,00	0	0	0,00	2	1	10,16	0	0	0,00
254574182	C4R8U6	PXL1	hypothetical protein	5,15	71836	1	1	10,14	0	0	0,00	0	0	0,00	0	0	0,00	0	0	0,00	0	0	0,00	0	0	0,00	0	0	0,00
254574190	C4R8V0	PP7435_Chr4-0198	Major component of the proteasome	5,72	202848	1	1	10,14	0	0	0,00	0	0	0,00	0	0	0,00	0	0	0,00	0	0	0,00	0	0	0,00	0	0	0,00
254574210	C4R8W0	EIF5	Translation initiation factor eIF-5	4,56	43397	1	2	10,19	2	2	10,18	2	5	15,19	2	1	10,17	2	5	15,19	2	2	10,15	2	4	15,18	2	3	10,19
254574238	C4R8X4	GUT1	Glycerol kinase, converts glycerol to glycerol-3-phosphate	5,13	68135	1	1	10,14	1	1	10,11	0	0	0,00	0	0	0,00	0	0	0,00	0	0	0,00	2	5	20,16	0	0	0,00
254574242	C4R8X6	GLN1	Glutamine synthetase (GS), synthesizes glutamine from glutamate and ammonia	6,15	41375	2	10	30,27	2	7	25,25	2	5	10,19	0	0	0,00	2	7	19,20	1	1	10,13	2	12	20,31	2	2	10,16
254574244	C4R8X7	PP7435_Chr4-0169	Cytosolic superoxide dismutase	5,93	15650	0	0	0,00	0	0	0,00	0	0	0,00	0	0	0,00	0	0	0,00	2	2	15,15	0	0	0,00	2	2	15,15
254574248	C4R8X9	PP7435_Chr4-0167	Mitochondrial ribosomal protein of the small subunit	5,11	29216	0	0	0,00	0	0	0,00	0	0	0,00	0	0	0,00	0	0	0,00	1	1	10,11	0	0	0,00	0	0	0,00
254574258	C4R8Y4	AMS1	Vacuolar alpha mannosidase, involved in free oligosaccharide (fOS) degradation	5,90	124829	1	1	10,16	2	1	10,16	0	0	0,00	0	0	0,00	0	0	0,00	0	0	0,00	0	0	0,00	0	0	0,00
254574268	C4R8Y9	PP7435_Chr4-0156	Homoaconitase, catalyzes the conversion of homocitrate to homoisocitrate, which is a step in the lys	5,56	75007	0	0	0,00	0	0	0,00	0	0	0,00	1	1	10,13	2	1	10,19	0	0	0,00	0	0	0,00	0	0	0,00
254574270	C4R8Z0	NHP2	Nuclear protein related to mammalian high mobility group (HMG) proteins	10,01	16792	0	0	0,00	0	0	0,00	0	0	0,00	0	0	0,00	0	0	0,00	1	1	10,11	0	0	0,00	0	0	0,00
254574274	C4R8Z2	PP7435_Chr4-0153	Essential beta-coat protein of the COPI coatomer	5,28	106250	2	1	10,14	2	3	10,17	1	2	10,15	1	1	10,16	1	3	10,17	0	0	0,00	1	2	10,17	1	1	10,14
254574296	C4R903	PAS_chr4_0808	Nucleolar protein, component of the small subunit processome complex, which is required for processi	10,56	33598	0	0	0,00	0	0	0,00	1	1	10,14	1	1	10,15	0	0	0,00	0	0	0,00	1	3	20,15	1	2	10,14
254574316	C4R913	PP7435_Chr4-0134	Ran GTPase binding protein	5,65	25186	2	9	25,25	2	8	20,24	2	24	10,25	2	8	10,22	2	21	20,26	2	7	10,23	2	6	10,23	2	4	10,25
254574322	C4R916	PAS_chr4_0820	Putative protein of unknown function	10,10	36016	0	0	0,00	0	0	0,00	0	0	0,00	0	0	0,00	0	0	0,00	0	0	0,00	0	0	0,00	1	1	10,13
254574326	C4R918	CBP1	Polyamine oxidase, converts spermine to spermidine	5,22	48809	0	0	0,00	0	0	0,00	1	1	10,11	0	0	0,00	0	0	0,00	0	0	0,00	0	0	0,00	0	0	0,00
254574342	C4R926	PP7435_Chr4-0120	Plasma membrane ATP-binding cassette (ABC) transporter	6,92	170506	0	0	0,00	0	0	0,00	0	0	0,00	0	0	0,00	0	0	0,00	0	0	0,00	1	1	10,16	2	2	10,12
254574358	C4R934	PAS_chr4_0840	Cytoplasmic glutaredoxin, thioltransferase, glutathione-dependent disulfide oxidoreductase	8,72	14524	0	0	0,00	0	0	0,00	0	0	0,00	0	0	0,00	2	1	10,21	0	0	0,00	0	0	0,00	1	2	10,24
254574362	C4R936	GLO1	Monomeric glyoxalase I	5,58	36628	2	8	60,20	1	1	16,17	0	0	0,00	0	0	0,00	0	0	0,00	0	0	0,00	1	1	10,14	0	0	0,00
254574372	C4R941	PP7435_Chr4-0104	Self-glucosylating initiator of glycogen synthesis, also glucosylates n-dodecyl-beta-D-maltoside	4,33	47407	0	0	0,00	0	0	0,00	0	0	0,00	0	0	0,00	0	0	0,00	1	1	10,16	0	0	0,00	0	0	0,00
254574392	C4R951	PAS_chr4_0857	hypothetical protein	8,86	70265	0	0	0,00	0	0	0,00	0	0	0,00	0	0	0,00	0	0	0,00	1	1	10,11	0	0	0,00	0	0	0,00
254574442	C4R976	PP7435_Chr4-0004	Translation initiation factor eIF1	8,57	12422	0	0	0,00	0	0	0,00	0	0	0,00	0	0	0,00	0	0	0,00	0	0	0,00	1	1	10,13	0	0	0,00
254574464	C4R987	PP7435_Chr4-0059	mitochondrial ATP synthase	7,50	10650	1	1	10,17	1	2	10,19	0	0	0,00	0	0	0,00	0	0	0,00	0	0	0,00	0	0	0,00	0	0	0,00
254574502	C4R9A6	PP7435_Chr4-0027	ATPase in ER, nuclear membrane and cytosol with homology to mammalian p97	4,68	91053	1	1	10,13	0	0	0,00	2	5	40,22	2	3	20,20	2	14	70,24	1	1	10,14	2	20	90,24	2	10	60,23
254574530	C4R9C0	PP7435_Chr4-0013	40S ribosomal protein S10	9,47	13189	0	0	0,00	0	0	0,00	0	0	0,00	1	1	10,12	0	0	0,00	1	1	10,15	1	1	10,13	0	0	0,00
308152294	E1UWE2	ACP1	ACPM1 (SDAP) subunit of mitochondrial NADH:ubiquinone oxidoreductase (complex I)	4,82	15464	0	0	0,00	0	0	0,00	1	2	10,13	1	1	10,15	0	0	0,00	2	3	10,19	0	0	0,00	2	2	10,12
328349873	F2QLY5	RIM4	Embryonic polyadenylate-binding protein B	6,28	74148	0	0	0,00	0	0	0,00	0	0	0,00	1	1	10,15	0	0	0,00	0	0	0,00	0	0	0,00	0	0	0,00
328350030	F2QNJ7	PP7435_Chr1-0269	hypothetical protein PP7435_Chr1-0269	6,08	17608	0	0	0,00	0	0	0,00	0	0	0,00	0	0	0,00	0	0	0,00	0	0	0,00	1	1	10,11	0	0	0,00
328351213	F2QQD5	CYP1	peptidylprolyl isomerase	6,11	20040	0	0	0,00	0	0	0,00	0	0	0,00	0	0	0,00	0	0	0,00	2	2	10,14	1	1	10,13	0	0	0,00
328351909	F2QSD0	ATIC	phosphoribosylaminoimidazolecarboxamide formyltransferase / IMP cyclohydrolase	6,56	64966	0	0	0,00	1	1	10,13	1	1	10,11	0	0	0,00	2	1	10,12	0	0	0,00	0	0	0,00	0	0	0,00
328352125	F2QSZ6	XOG1	glucan 1,3-beta-glucosidase similar to S. cerevisiae EXG1 (YLR300W)	4,34	43977	0	0	0,00	0	0	0,00	0	0	0,00	0	0	0,00	0	0	0,00	0	0	0,00	2	2	10,26	0	0	0,00
328352191	F2QT62	AAP1	Aminopeptidase N	4,50	23000	2	3	25,16	2	2	20,21	2	2	10,15	0	0	0,00	2	5	30,26	2	9	10,16	2	14	55,31	2	4	20,30
328352429	F2QTV0	SHP1	NSFL1 cofactor p47	4,92	39370	0	0	0,00	0	0	0,00	0	0	0,00	0	0	0,00	0	0	0,00	0	0	0,00	1	1	10,18	0	0	0,00
328353017	F2QVI7	MEU1	5'-methylthioadenosine phosphorylase	6,11	34011	0	0	0,00	0	0	0,00	0	0	0,00	1	1	10,12	0	0	0,00	0	0	0,00	0	0	0,00	0	0	0,00
328353048	F2QVL8	MTR4	ATP-dependent RNA helicase DOB1	5,87	120398	0	0	0,00	0	0	0,00	0	0	0,00	0	0	0,00	0	0	0,00	0	0	0,00	2	2	10,18	0	0	0,00
328353086	F2QVQ6	PP7435_Chr3-0524	hypothetical protein PP7435_Chr3-0524	5,45	58146	0	0	0,00	0	0	0,00	0	0	0,00	0	0	0,00	1	1	10,14	0	0	0,00	0	0	0,00	0	0	0,00
328353173	F2QVZ3	PP7435_Chr3-0614	molybdenum cofactor sulfurase protein (HxB),putative	6,60	54120	1	1	10,11	0	0	0,00	0	0	0,00	0	0	0,00	0	0	0,00	0	0	0,00	0	0	0,00	0	0	0,00
328353568	F2QX38	ELC1	Transcription elongation factor B polypeptide 1	3,93	11890	0	0	0,00	0	0	0,00	0	0	0,00	0	0	0,00	0	0	0,00	0	0	0,00	1	1	10,11	0	0	0,00
328353629	F2QX99	PP7435_Chr3-1084	Lactation elevated protein 1 homolog	8,13	90286	0	0	0,00	0	0	0,00	1	1	10,19	0	0	0,00	0	0	0,00	0	0	0,00	0	0	0,00	0	0	0,00
328353682	F2QXF2	PP7435_Chr3-1137	60S acidic ribosomal protein P2	3,71	11085	0	0	0,00	0	0	0,00	0	0	0,00	0	0	0,00	2	3	25,23	0	0	0,00	0	0	0,00	2	1	10,15
328353820	F2QXT9	UBP6	ubiquitin carboxyl-terminal hydrolase 14	5,90	58624	1	1	10,15	0	0	0,00	0	0	0,00	0	0	0,00	0	0	0,00	0	0	0,00	0	0	0,00	0	0	0,00
328353905	F2QY24	PP7435_Chr4-0127	Uncharacterized membrane protein YPR109W	4,51	33269	0	0	0,00	0	0	0,00	1	1	10,11	0	0	0,00	0	0	0,00	0	0	0,00	0	0	0,00	0	0	0,00
328353940	F2QY59	PP7435_Chr4-0162	Subtilisin E	4,93	8191	2	1	10,16	0	0	0,00	0	0	0,00	0	0	0,00	2	1	10,25	1	1	10,13	2	1	10,16	2	2	10,19
328353969	F2QY88	PP7435_Chr4-0191	Protein PUF2	6,55	92334	0	0	0,00	0	0	0,00	0	0	0,00	0	0	0,00	0	0	0,00	0	0	0,00	1	1	10,13	0	0	0,00
328353998	F2QYB7	CTK2	Cyclin-L2	5,10	28497	0	0	0,00	0	0	0,00	1	1	10,13	0	0	0,00	0	0	0,00	0	0	0,00	0	0	0,00	0	0	0,00
328354180	F2QYU9	PP7435_Chr4-0410	hypothetical protein PP7435_Chr4-0410	4,60	8948	1	2	10,15	0	0	0,00	0	0	0,00	0	0	0,00	0	0	0,00	0	0	0,00	0	0	0,00	0	0	0,00
328354224	F2QYZ3	gua1	IMP dehydrogenase	6,85	56091	0	0	0,00	1	1	10,11	2	4	10,17	1	1	10,12	0	0	0,00	1	2	10,13	2	2	15,15	1	2	10,15
328354459	F2QZM8	PP7435_Chr4-0699	26 proteasome complex subunit DSS1	4,07	8827	2	2	10,24	2	2	10,23	2	3	10,24	1	2	10,26	2	1	10,23	2	2	10,24	2	2	10,24	2	1	10,25
328354527	F2QZU6	ADE3	methylenetetrahydrofolate dehydrogenase (NADP+) / methenyltetrahydrofolate cyclohydrolase / formyltetrahydrofolate synthetase	5,86	32251	2	2	15,23	2	2	20,22	2	5	20,23	0	0	0,00	2	4	30,21	0	0	0,00	2	9	35,22	2	3	25,19
328354528	F2QZU7	PP7435_Chr4-0771	methylenetetrahydrofolate dehydrogenase (NADP+) / methenyltetrahydrofolate cyclohydrolase / formyltetrahydrofolate synthetase	5,86	70692	0	0	0,00	0	0	0,00	0	0	0,00	0	0	0,00	0	0	0,00	0	0	0,00	0	0	0,00	2	2	15,18
328354583	F2R002	PP7435_Chr4-0827	Absent in melanoma 1 protein	7,18	65765	0	0	0,00	0	0	0,00	0	0	0,00	0	0	0,00	0	0	0,00	0	0	0,00	2	5	35,30	2	2	20,22
328354771	F2R0J0	ALS3	Agglutinin-like protein 3	3,59	35868	0	0	0,00	0	0	0,00	1	1	10,13	0	0	0,00	0	0	0,00	0	0	0,00	0	0	0,00	0	0	0,00
328672360	F2R0J7	COX2	cytochrome c oxidase subunit 2	4,11	28421	2	1	10,11	2	1	10,12	0	0	0,00	0	0	0,00	0	0	0,00	0	0	0,00	0	0	0,00	0	0	0,00
328672373	F2R0L0	ATP6	ATP synthase subunit 6	8,89	28088	0	0	0,00	2	1	10,14	0	0	0,00	0	0	0,00	0	0	0,00	0	0	0,00	0	0	0,00	0	0	0,00

Table S2. Differentially expressed proteins in ATX3-trasformed versus the control (transformed with empty vector) *P. pastoris* strain grown at 30°C for 48 h. For each protein are reported: GI Accession number, Uniprot entry, Gene name, Reference, pI, MW, DAve, DCI and molecular function. Positive values for DAve and DCI indicate that the protein is more abundant in the ATX3-trasformed strain; negative values the control strain. For further details regarding the meaning and the confidence range applied to DAve and DCI see Materials and Methods.

Accession	Uniprot Entry	Gene names	Reference	pI	MW	Dave	DCI	Molecular Function
254574362	C4R936	GLO1	Monomeric glyoxalase I	5,60	36628	2,00	1812	Pyruvate metabolism
254573470	C4R7U0	PAS_chr4_0416	Alanine:glyoxylate aminotransferase (AGT), catalyzes the synthesis of glycine from glyoxylate	8,30	45383	2,00	806	Biosynthetic enzymes - Amino acids
254564717	C4QVB5	CPGL	Probable di-and tri-peptidase	5,20	55999	0,99	404	Stress proteins - Oxidative stress
254570667	C4R3X8	PP7435_Chr3-0996	ATPase involved in protein folding and the response to stress	4,90	70706	0,87	5501	Stress proteins - Chaperones, Heat shock
254571699	C4R5E4	PP7435_Chr3-0464	Cytoplasmic ATPase that is a ribosome-associated molecular chaperone	4,90	66447	0,54	416	Stress proteins - Chaperones, Heat shock
254566601	C4QY07	PGK1	3-phosphoglycerate kinase	8,70	44058	0,52	2369	Glycolysis - Amphibolic
254571145	C4R4L7	PGI1	Glycolytic enzyme phosphoglucose isomerase	5,80	61928	0,50	5021	Glycolysis - Amphibolic
254573464	C4R7T7	PP7435_Chr4-0572	60S ribosomal protein L13	11,20	22134	0,46	490	Protein biosynthesis - Ribosomal proteins
254565545	C4QWH9	IDP1	Mitochondrial NADP-specific isocitrate dehydrogenase, catalyzes the oxidation of isocitrate	8,30	48460	-0,46	-490	Mitochondrial energy metabolism - Krebs cycle
254569162	C4R1N7	CMD1	Calmodulin	4,20	16850	-0,80	-503	Unclear
254566607	C4QY10	PP7435_Chr1-1078	Alpha subunit of fatty acid synthetase	5,20	206000	-2,00	-455	Biosynthetic enzymes - Fatty acids

Table S3. Differentially expressed proteins at 30°C versus 37°C at 48 h of growth in the ATX3-trasformed *P. pastoris* . For each protein are reported: GI Accession number, Uniprot entry, Gene name, Reference, pI, MW, DAve, DCI and Molecular Function. Positive values for DAve and DCI indicate that a protein is more abundant at 30°C; negative values at 37°C. For further details regarding the meaning and the confidence range applied to DAve and DCI see Materials and Methods. Proteins were primarily grouped according to their molecular function and secondarily according to their descending DAve values.

Accession	Entry Uniprot	Gene names	Reference	pI	MW	Dave	DCI	Molecular Function	
254573464	C4R7T7	PP7435_Chr4-0572	60S ribosomal protein L13	11,20	22134	0,90	692	Ribosomal proteins	Protein biosynthesis
254567233	C4QYX3	RPS2	40S ribosomal protein S2	10,40	28190	0,85	1056		
254565783	C4QWU8	PP7435_Chr1-0662	60S ribosomal protein L5	8,70	34099	-0,46	-492		
254572856	C4R6Y3	RPL6	60S ribosomal protein L6	9,00	18577	-0,90	-693		
254564587	C4QV50	RPP0	60S acidic ribosomal protein P0	4,50	33684	-2,00	-1211		
116293731	A0FJH5		translation elongation factor 1-alpha	9,40	50098	-1,43	-7028	Elongation factors	
254571359	C4R4X4	TUF1	Mitochondrial translation elongation factor Tu	5,50	46770	-2,00	-2124		
254572668	C4R6N9	CEF3	Translational elongation factor 3, stimulates the binding of aminoacyl-tRNA (AA-tRNA) to ribosomes	5,47	116532	-2,00	-2126		
254570979	C4R4D4	GARS	Cytoplasmic and mitochondrial glycyl-tRNA synthase	5,20	74111	-1,07	-1147	Aminoacyl-tRNA Synthetases	
254566971	C4QYJ2	SES1	Cytosolic seryl-tRNA synthetase, class II aminoacyl-tRNA synthetase	5,66	52822	-2,00	-809		
254566127	C4QXC0	PAS_chr1-4_0063	G-protein beta subunit and guanine nucleotide dissociation inhibitor for Gpa2p	6,30	34652	0,99	404	Messenger RNA biogenesis	
254568492	C4R0Q2	GSP1	GTP binding protein (mammalian Ranp homolog)	6,61	24067	-2,00	-814	Ribosome biogenesis	
254568216	C4R0B4	CAL1	Calnexin	4,40	63027	-1,19	-758	Protein processing in endoplasmic reticulum	
47132400	Q6GYJ7		methionine synthase	5,80	85966	0,77	7125	Amino acids	Biosynthetic enzymes
254571587	C4R588	ASN1	Asparagine synthetase, isozyme of Asn1p	5,50	65032	-0,54	-416		
254565959	C4QX36	ILV5	Acetohydroxyacid reductoisomerase	8,10	44265	-0,81	-3727		
254572005	C4R5U7	PAS_chr3_0876	S-adenosylmethionine synthetase	6,07	42331	-2,00	-456		
254570271	C4R3D0	LEU2	Beta-isopropylmalate dehydrogenase (IMDH), catalyzes the third step in the leucine biosynthesis path	4,70	39781	-2,00	-621		
254574028	C4R8L9	CAR1	Arginase, responsible for arginine degradation	5,30	35120	-2,00	-621		
224994879	C0LQF6	TKL1	transketolase 1, partial	5,80	74646	-0,46	-4400	Pentose phosphate pathway	
254568186	C4R099	ZWF1	Glucose-6-phosphate dehydrogenase (G6PD), catalyzes the first step of the pentose phosphate pathway	6,10	57635	-1,19	-3013		
254568470	C4R0P1	TDH1	Glyceraldehyde-3-phosphate dehydrogenase, isozyme 3, involved in glycolysis and gluconeogenesis	6,27	35563	-2,00	-618		
254569478	C4R245	TAL1	Transaldolase, enzyme in the non-oxidative pentose phosphate pathway	4,93	35478	-2,00	-619		
328354527	F2QZU6	ADE3	methylenetetrahydrofolate dehydrogenase (NADP+) / methenyltetrahydrofolate cyclohydrolase / formyltetrahydrofolate synthetase	5,90	32251	-0,79	-504	Purine	
254566607	C4QY10	PP7435_Chr1-1078	Alpha subunit of fatty acid synthetase	5,20	206000	-2,00	-808	Fatty acids	
254568036	C4R024	MDH1	Mitochondrial malate dehydrogenase, catalyzes interconversion of malate and oxaloacetate	6,40	34868	-1,42	-1760	Gluconeogenetic-Anaplerotic	
1871627	P78992	PYC1	pyruvate carboxylase	6,00	131400	-2,00	-1527		
254572145	C4R617	PP7435_Chr3-0229	Coproporphyrinogen III oxidase, an oxygen requiring enzyme	5,80	37282	0,99	405	Miscellaneous	
254570088	C4R300	YNK1	Nucleoside diphosphate kinase	6,20	16943	-1,11	-568		
254574124	C4R8R7	PAS_chr4_0730	Inositol monophosphatase, involved in biosynthesis of inositol	4,60	26214	-2,00	-810		
254573296	C4R7K3	PP7435_Chr4-0659	Putative dihydrokaempferol 4-reductase	5,41	39527	-2,00	-894		
254571125	C4R4K7	PP7435_Chr3-0765	Putative dihydrokaempferol 4-reductase	6,02	39071	-2,00	-3218		
254571131	C4R4L0	PP7435_Chr3-0762	Putative dihydrokaempferol 4-reductase	6,02	39202	-2,00	-7842		
254571145	C4R4L7	PGI1	Glycolytic enzyme phosphoglucose isomerase	5,80	61928	0,86	6600	Amphibolic	Glycolysis
254565205	C4QW09	FBA1	Fructose 1,6-bisphosphate aldolase, required for glycolysis and gluconeogenesis	6,00	39689	-0,59	-1071		
254573908	C4R8F9	EMI2	Non-essential protein of unknown function required for transcriptional induction	6,10	52325	-0,66	-604		
254568544	C4R0S8	ADH	Mitochondrial alcohol dehydrogenase isozyme III	5,80	36958	0,73	7700	Catabolic	
254567173	C4QYU3	HXK2	Hexokinase-2	5,40	55359	0,50	805		
254571043	C4R4G6	acsA	Acetyl-coA synthetase isoform	5,40	74000	-2,00	-621	Ethanol assimilation	
238034194	C4R9E0	PP7435_Chr1-1573	Alcohol dehydrogenase	6,35	37816	-2,00	-2060		
254568616	C4R0W4	PP7435_Chr2-0787	hypothetical protein	5,90	56736	-2,00	-3218		
254571699	C4R5E4	PP7435_Chr3-0464	Cytoplasmic ATPase that is a ribosome-associated molecular chaperone	4,90	66447	2,00	620	Chaperones - Heat shock	Stress proteins
254573764	C4R887	HSA1	ATPase involved in protein folding and nuclear localization signal (NLS)-directed nuclear transport	4,70	69574	-0,78	-1230		
238034205	C4R9F1	SSE1	Heat shock protein	5,00	78709	-1,23	-3428		
254572906	C4R708	PAS_chr4_0158	Tetradecameric mitochondrial chaperonin	4,90	60138	-1,34	-25495		
254566257	C4QXI5	PP7435_Chr1-0897	Heat shock protein Hsp90	4,70	80833	-1,49	-2414		
254566845	C4QYC9	PAS_chr1-4_0404	hypothetical protein	4,80	28332	-2,00	-455		
254569506	C4R259	MDJ1	Co-chaperone that stimulates the ATPase activity of the HSP70 protein Ssc1p	9,44	53055	-2,00	-456		
254566327	C4QXM0	PP7435_Chr1-0932	Putative protein of unknown function	9,60	11129	-2,00	-2126		
254571369	C4R4X9	CPR6	Peptidyl-prolyl cis-trans isomerase (cyclophilin)	5,12	40035	0,99	404	Peptidyl-prolyl cis-trans isomerase	
254569714	C4R2G3	PP7435_Chr2-0241	hypothetical protein	4,20	45634	-0,93	-1325		
238034215	C4R9G1	PAS_c034_0039	Peptidyl-prolyl isomerase	6,19	85597	-2,00	-1260		
254572796	C4R6V3	CAM1	Nuclear protein required for transcription of MXR1	6,10	47062	-0,99	-403	Oxidative stress	
254569734	C4R2H3	TSA1	Thioredoxin peroxidase, acts as both a ribosome-associated and free cytoplasmic antioxidant	4,70	21418	-1,00	-4921		
254569682	C4R2E7	TRR1	hypothetical protein	5,10	34343	-2,00	-1022		
254569930	C4R2S1	CAT1	Catalase A, breaks down hydrogen peroxide in the peroxisomal matrix formed by acyl-CoA oxidase (Pox1	6,59	57810	-2,00	-1813		
254566355	C4QXN4	PP7435_Chr1-0948	hypothetical protein	4,82	17859	-2,00	-2129		
254571727	C4R5F8	PP7435_Chr3-0448	Aldose reductase involved in methylglyoxal, d-xylose and arabinose metabolism	5,90	36095	-2,00	-806	Osmoprotection	
254569896	C4R2Q4	PAS_chr2-2_0148	Cell wall protein that contains a putative GPI-attachment site	3,71	43204	-2,00	-809	Cell wall damage	
254570112	C4R312	PP7435_Chr2-0049	Dihydrolipoamide dehydrogenase	6,30	52306	-0,53	-3016	Pyruvate dehydrogenase complex	Mitochondrial energy metabolism
254565157	C4QVY5	LAT1	Dihydrolipoamide acetyltransferase component (E2) of pyruvate dehydrogenase complex	6,60	50528	-2,00	-620		
254567788	C4QZQ0	IDH2	Subunit of mitochondrial NAD(+)-dependent isocitrate dehydrogenase, which catalyzes the oxidation of	8,05	39963	-2,00	-454	Krebs cycle	
254564647	C4QV80	KGD2	Dihydrolipoyl transsuccinylase, component of the mitochondrial alpha-ketoglutarate dehydrogenase	8,67	48038	-2,00	-1022		
254567720	C4QZL6	sucA	Component of the mitochondrial alpha-ketoglutarate dehydrogenase complex, which catalyzes a key step	6,22	112682	-2,00	-2465		
254565307	C4QW60	CIT1	hypothetical protein	8,56	51855	-2,00	-4438		
254565263	C4QW38	COR1	Core subunit of the ubiquinol-cytochrome c reductase complex (bc1 complex)	5,00	47391	-1,57	-3578	Respiratory chain	
254569858	C4R2N5	atpD	Beta subunit of the F1 sector of mitochondrial F1F0 ATP synthase	5,00	53974	-0,54	-2418	ATP synthase	
254571387	C4R4Y8	atpA	Alpha subunit of the F1 sector of mitochondrial F1F0 ATP synthase	9,40	58713	-0,90	-691		
254566933	C4QYH3	PP7435_Chr1-1240	Delta subunit of the central stalk of mitochondrial F1F0 ATP synthase	4,70	16963	-1,00	-1609		
254574362	C4R936	GLO1	Monomeric glyoxalase I	5,60	36628	2,00	1812	Pyruvate metabolism	
254571127	C4R4K8	PP7435_Chr3-0764	Putative dihydrokaempferol 4-reductase	5,75	39489	-2,00	-489		
254571861	C4R5M5	NUC1	hypothetical protein	5,90	54808	-2,00	-455	Cellular trafficking	
254568888	C4R1A0	SAC6	Fimbrin, actin-bundling protein	5,12	70161	-2,00	-2125		
254565475	C4QWE4	AAT1	Cytosolic aspartate aminotransferase	6,80	47734	0,44	904	Aminotransferases	
328352191	F2QT62	AAP1	Aminopeptidase N	4,50	23000	-0,75	-1213	Proteases (non-proteasomal)	
254572447	C4R6G8	36982	Vacuolar aspartyl protease (proteinase A)	4,49	44278	-2,00	-457		
254573760	C4R885	PP7435_Chr4-0424	Putative protein of unknown function	5,50	36098	-1,45	-4422	Vitamin B6 metabolism	
254565045	C4QVS9	PMA1	Plasma membrane H+-ATPase, pumps protons out of the cell	4,80	97797	0,80	2012	Unclear	
13235614	Q9C1Z8	pdi	protein disulphide isomerase	4,40	57735	-1,40	-3517		
254573508	C4R7V9	PP7435_Chr4-0551	NADPH-dependent alpha-keto amide reductase	5,51	33673	-2,00	-455		
254572936	C4R723	uca	Urea amidolyase, contains both urea carboxylase and allophanate hydrolase activities	5,40	201006	-2,00	-456		
328354583	F2R002	PP7435_Chr4-0827	Absent in melanoma 1 protein	7,18	65765	-2,00	-623		
254570981	C4R4D5	PP7435_Chr3-0838	hypothetical protein	6,40	45175	-2,00	-1260		
254573846	C4R8C8	PP7435_Chr4-0378	Glyoxylate reductase	6,02	38163	-2,00	-1528		
254570673	Q9P4D1	ACT1	hypothetical protein	5,16	41647	-1,13	-3750		
254574502	C4R9A6	PP7435_Chr4-0027	ATPase in ER, nuclear membrane and cytosol with homology to mammalian p97	4,68	91053	-2,00	-4072		
254565975	C4QX44	PP7435_Chr1-0758	hypothetical protein	6,40	11826	2,00	457	Unassigned	
254567189	C4QYV1	SLB2	Primary component of eisosomes	4,90	35209	0,54	1659		
254571875	C4R5N2	PP7435_Chr3-0372	Protein of unknown function involved in vacuolar protein sorting	6,50	94532	-0,75	-1205		
254568908	C4R1B0	PP7435_Chr2-0640	Putative protein of unknown function	6,40	21714	-2,00	-455		
254565273	C4QW43	PP7435_Chr1-0406	Putative protein proposed to be involved in the metabolism of purine and pyrimidine base analogues	4,96	24834	-2,00	-621		
254567447	C4QZ80	PP7435_Chr1-1506	hypothetical protein	5,62	37893	-2,00	-1813		
254567798	C4QZQ5	EFT1	hypothetical protein	6,30	93392	-2,00	-1934		

Table S4. Differentially expressed proteins, as shown by the MAProMa comparison between the ATX3-trasformed versus the control (transformed with empty vector) *P. pastoris* strains grown at 37°C at the three times monitored (16 h, 24 h and 48 h). The differentially expressed proteins in the JD-trasformed and JDC14A-trasformed P. pastoris strains (grown at 37°C for 48 h) are also reported. For each protein are reported: Molecular Function, GI Accession number, Uniprot entry, Gene name, Reference, pI, MW, Dave and DCI. Positive values for DAve and DCI indicate that a protein is more abundant in the ATX3- or in the JD-trasformed strain; negative in the control or in the JD-C14a strain, respectively. For further details regarding the meaning and the confidence range applied to DAve and DCI see Materials and Methods. The hyphen identifies proteins whose Dave and/or DCI is below the confidence threshold set (DAve |0.40| and/or DCI |400|). Proteins were primarily grouped according to their molecular function and secondarily according to their increasing GI Accession numbers.

Molecular Function		Accession	Uniprot Entry	Gene names	Reference	pI	MW	ATX3Q26 vs Ctrl						JD vs JDC14A	
								16h		24h		48h		48h	
								DAve	DCI	DAve	DCI	DAve	DCI	DAve	DCI
Protein biosynthesis	Ribosomal proteins	254564587	C4QV50	RPP0	60S acidic ribosomal protein P0	4,50	33684	0,73	1151	-	-	0,48	755	-	-
		254565783	C4QWU8	PP7435_Chr1-0662	60S ribosomal protein L5	8,70	34099	-	-	0,99	405	0,66	605	-	-
		254567027	C4QYM0	PAS_chr1-4_0490	60S acidic ribosomal protein P2	3,73	12207	-	-	-	-	-	-	0,47	1318
		254572856	C4R6Y3	RPL6	60S ribosomal protein L6	9,00	18577	-	-	-	-	0,66	605	-	-
		254574530	C4R9C0	PP7435_Chr4-0013	40S ribosomal protein S10	9,47	13189	-	-	-	-	-	-	2,00	455
		254573464	C4R7T7	PP7435_Chr4-0572	60S ribosomal protein L13	11,20	22134	0,80	504	-	-	-	-	-	-
		328353682	F2QXF2	PP7435_Chr3-1137	60S acidic ribosomal protein P2	3,71	11085	-	-	-	-	-	-	-0,57	-706
	Elongation factors	116293731	A0FJH5		translation elongation factor 1-alpha	9,40	50098	1,00	6417	-	-	-	-	0,55	4398
		254571359	C4R4X4	TUF1	Mitochondrial translation elongation factor Tu	5,50	46770	0,75	1207	-0,77	-470	-	-	-	-
		254572668	C4R6N9	CEF3	Translational elongation factor 3, stimulates the binding of aminoacyl-tRNA (AA-tRNA) to ribosomes	5,47	116532	-	-	-	-	0,62	1542	-	-
	Aminoacyl-tRNA Synthetases	254566971	C4QYJ2	SES1	Cytosolic seryl-tRNA synthetase, class II aminoacyl-tRNA synthetase	5,66	52822	1,10	569	1,10	568	0,90	693	-	-
		254567253	C4QYY3	PP7435_Chr1-1407	Glutamyl-tRNA synthetase, cytoplasmic	6,30	82285	-	-	2,00	456	-	-	-	-
		254570979	C4R4D4	GARS	Cytoplasmic and mitochondrial glycyl-tRNA synthase	5,20	74111	1,27	970	-0,46	-490	-	-	-	-
	Ribosome biogenesis	254572962	C4R736	NSR1	Nucleolar protein that binds nuclear localization sequences	4,88	38774	-	-	-	-	-	-	0,44	906
	mRNA biogenesis	254566127	C4QXC0	PAS_chr1-4_0063	G-protein beta subunit and guanine nucleotide dissociation inhibitor for Gpa2p	6,33	34652	-	-	-	-	-	-	0,77	469
Biosynthetic enzymes	Amino acids	47132400	Q6GYJ7		methionine synthase	5,80	85966	2,00	455	2,00	768	-	-	-	-
		254565279	C4QW46	GDH1	NADP(+)-dependent glutamate dehydrogenase	5,40	49258	0,77	819	-	-	-	-	-	-
		254565959	C4QX36	ILV5	Acetohydroxyacid reductoisomerase	8,10	44265	1,19	2643	-	-	-	-	-	-
		254567890	C4QZV1	PP7435_Chr2-1141	Protein that is processed in the mitochondrion to yield acetylglutamate kinase and N-acetyl-gamma-gl	7,00	92546	2,00	456	-	-	-	-	-	-
		254568732	C4R122	CYK1	hypothetical protein	7,90	40375	-	-	-	-	-2,00	-457	-	-
		254570633	C4R3W1	ARG1	Arginosuccinate synthetase	5,00	46380	0,82	1495	-	-	0,54	415	-	-
		254571179	C4R4N4	HOM2	Aspartic beta semi-aldehyde dehydrogenase	5,40	38650	-	-	2,00	619	-0,99	-404	-	-
		254571587	C4R588	ASN1	Asparagine synthetase, isozyme of Asn1p	5,50	65032	2,00	457	-	-	-	-	-	-
		254573470	C4R7U0	PAS_chr4_0416	Alanine:glyoxylate aminotransferase (AGT), catalyzes the synthesis of glycine from glyoxylate astor	8,30	45383	-	-	-	-	0,50	803	0,93	1322
		254574028	C4R8L9	CAR1	Arginase, responsible for arginine degradation	5,30	35120	-	-	-	-	1,11	569	-	-
	Pentose phosphate pathway	224994879	C0LQF6	TKL1	transketolase 1, partial	5,80	74646	2,00	621	0,67	1849	0,74	5705	-	-
		254568186	C4R099	ZWF1	Glucose-6-phosphate dehydrogenase (G6PD), catalyzes the first step of the pentose phosphate pathway	6,10	57635	2,00	621	-	-	1,04	2900	-	-
		254568470	C4R0P1	TDH1	Glyceraldehyde-3-phosphate dehydrogenase, isozyme 3, involved in glycolysis and gluconeogenesis ast	6,27	35563	-	-	-	-	0,79	503	-	-
		254569478	C4R245	TAL1	Transaldolase, enzyme in the non-oxidative pentose phosphate pathway	4,93	35478	-	-	-2,00	-425	2,00	619	-	-
		254570771	C4R430	PGD	6-phosphogluconate dehydrogenase (decarboxylating)	5,90	53929	-	-	0,66	1360	0,68	2372	-	-
	Purine	254573886	C4R8E8	PP7435_Chr4-0357	Adenylosuccinate synthase	6,20	47720	1,19	756	-	-	-	-	-	-
		328354527	F2QZU6	ADE3	methylenetetrahydrofolate dehydrogenase (NADP+) / methenyltetrahydrofolate cyclohydrolase / formyltetrahydrofolate synthetase	5,90	32251	-	-	2,00	456	-	-	-	-
	Fatty acids	254565063	C4QVT8	FAS1	Beta subunit of fatty acid synthetase	6,20	229580	-	-	2,00	1022	2,00	1813	0,72	403
		254566607	C4QY10	PP7435_Chr1-1078	Alpha subunit of fatty acid synthetase	5,20	206000	-	-	-	-	2,00	808	0,94	2149
	Gluconeogenetic-Anapleorotic	1871627	P78992	PYC1	pyruvate carboxylase	6,00	131400	-	-	-	-	1,14	1412	0,74	436
		254568036	C4R024	MDH1	Mitochondrial malate dehydrogenase, catalyzes interconversion of malate and oxaloacetate astoris GS	6,40	34868	-	-	-	-	1,19	1696	-	-
	Miscellaneous	2624379	O13505	IPP1	inorganic pyrophosphatase (pyrophosphate phospho-hydrolase)	5,28	32030	-	-	-2,00	-456	-	-	0,46	491
		254564917	C4QVL5	PHR2	Beta-1,3-glucanosyltransferase, required for cell wall assembly	3,84	58251	-	-	-	-	-2,00	-2467	-1,31	-1158
		254570034	C4R2X3	PAS_chr2-2_0082	hypothetical protein	7,60	16056	-	-	-	-	-2,00	-1811	-	-
		254570088	C4R300	YNK1	Nucleoside diphosphate kinase	6,20	16943	-	-	-	-	-0,83	-3012	-	-
		254571125	C4R4K7	PP7435_Chr3-0765	Putative dihydrokaempferol 4-reductase	6,02	39071	0,78	864	-	-	-	-	0,44	904
		254571131	C4R4L0	PP7435_Chr3-0762	Putative dihydrokaempferol 4-reductase	6,02	39202	0,99	403	-0,80	-2007	-	-	-	-
		254572145	C4R617	PP7435_Chr3-0229	Coproporphyrinogen III oxidase, an oxygen requiring enzyme	5,80	37282	-	-	0,54	417	-	-	-	-
		254573296	C4R7K3	PP7435_Chr4-0659	Putative dihydrokaempferol 4-reductase	5,41	39527	-	-	-2,00	-730	-	-	-	-
		254574124	C4R8R7	PAS_chr4_0730	Inositol monophosphatase, involved in biosynthesis of inositol	4,60	26214	-	-	-	-	0,46	491	-	-
Glycolysis	Amphibolic	254565205	C4QW09	FBA1	Fructose 1,6-bisphosphate aldolase, required for glycolysis and gluconeogenesis	6,02	39689	-	-	-	-	-	-	0,49	3111
		254566601	C4QY07	PGK1	3-phosphoglycerate kinase	8,70	44058	1,27	971	-	-	2,00	2397	-	-
		254570367	C4R3H8	ENO2	Enolase I, a phosphopyruvate hydratase that catalyzes the conversion of 2-phosphoglycerate to phosph	5,30	46481	1,03	7032	-	-	-	-	-	-
		254571145	C4R4L7	PGI1	Glycolytic enzyme phosphoglucose isomerase	5,81	61928	-	-	-	-	-	-	-0,66	-605
	Catabolic	254567173	C4QYU3	HXK2	Hexokinase-2	5,40	55359	-	-	-	-	-0,59	-1070	-	-
		254568544	C4R0S8	ADH	Mitochondrial alcohol dehydrogenase isozyme III	5,80	36958	-	-	-0,66	-943	-	-	-	-
		254569186	C4R1P9	PYK2	Pyruvate kinase	6,10	55573	0,90	694	1,10	567	-	-	-	-
		254570575	C4R3T2	PDC1	Major of three pyruvate decarboxylase isozymes	5,60	61304	0,64	10026	0,45	8228	0,51	8308	-	-
Ethanol assimilation		238034194	C4R9E0	PP7435_Chr1-1573	Alcohol dehydrogenase	6,35	37816	-	-	-	-	2,00	2060	-	-
		254568616	C4R0W4	PP7435_Chr2-0787	hypothetical protein	5,90	56736	-	-	-	-	0,46	1957	0,52	2375
		254571043	C4R4G6	acsA	Acetyl-coA synthetase isoform	5,40	74000	-	-	-	-	2,00	621	-	-
		254572239	C4R664	ALD5	Mitochondrial aldehyde dehydrogenase	5,40	53935	-	-	2,00	455	0,80	504	-	-
	Chaperones - Heat shock	90110409	Q1W5W1		heat shock protein 70	4,90	66435	0,75	774	-	-	0,74	991	-	-
		238034205	C4R9F1	SSE1	Heat shock protein	5,00	78709	0,62	2328	-	-	-	-	-	-
		254566089	C4QXA1	SBA1	Co-chaperone that binds to and regulates Hsp90 family chaperones	4,11	22441	-	-	-1,10	-567	-1,10	-566	-	-
		254566257	C4QXI5	PP7435_Chr1-0897	Heat shock protein Hsp90	4,70	80833	-	-	-0,65	-1577	0,80	2011	0,45	2280
		254566327	C4QXM0	PP7435_Chr1-0932	Putative protein of unknown function	9,60	11129	0,99	404	-	-	-	-	-0,46	-1957
		254568132	C4R072	ZUO1	Cytosolic ribosome-associated chaperone	7,72	50821	0,66	604	0,99	403	-	-	-	-
		254569506	C4R259	MDJ1	Co-chaperone that stimulates the ATPase activity of the HSP70 protein Ssc1p	9,44	53055	-	-	-	-	2,00	456	-	-
		254570072	C4R2Z2	PP7435_Chr2-0071	Protein chaperone involved in regulation of the HSP90 and HSP70 functions	5,80	43370	2,00	1021	0,91	1266	-	-	0,41	628
		254570667	C4R3X8	PP7435_Chr3-0996	ATPase involved in protein folding and the response to stress	4,90	70706	-	-	0,55	2762	-	-	-	-

								DAve	DCI	DAve	DCI	DAve	DCI	DAve	DCI
Stress proteins		254570957	C4R4C3	SSC1	Mitochondrial matrix ATPase	5,30	69648	0,53	4772	0,69	13108	-	-	-	-
		254571699	C4R5E4	PP7435_Chr3-0464	Cytoplasmic ATPase that is a ribosome-associated molecular chaperone	4,90	66447	-	-	0,99	406	-	-	-	-
		254572906	C4R708	PAS_chr4_0158	Tetradecameric mitochondrial chaperonin	4,90	60138	0,71	12259	-	-	-	-	-	-
		254573180	C4R7E5	TRX2	Cytoplasmic thioredoxin isoenzyme of the thioredoxin system	4,70	11252	-	-	-	-	-	-	0,79	503
		254573764	C4R887	HSA1	ATPase involved in protein folding and nuclear localization signal (NLS)-directed nuclear transport	4,70	69574	0,80	2009	-	-	-	-	-	-
	Peptidyl-prolyl cis-trans isomerase	238034215	C4R9G1	PAS_c034_0039	Peptidyl-prolyl isomerase	6,19	85597	-	-	-2,00	-455	-	-	-	-
		254569714	C4R2G3	PP7435_Chr2-0241	hypothetical protein	4,20	45634	0,50	807	-	-	-	-	-	-
		254571369	C4R4X9	CPR6	Peptidyl-prolyl cis-trans isomerase (cyclophilin)	5,12	40035	-	-	-2,00	-808	-	-	-	-
	Oxidative stress	254565961	C4QX37	PP7435_Chr1-0751	Mitochondrial peroxiredoxin (1-Cys Prx) with thioredoxin peroxidase activity	5,71	25102	-	-	-	-	-	-	0,46	491
		254566141	C4QXC7	SOD2	Mitochondrial superoxide dismutase, protects cells against oxygen toxicity	8,79	25140	-	-	-	-	-	-	0,54	415
		254566355	C4QXN4	PP7435_Chr1-0948	hypothetical protein	4,82	17859	-	-	2,00	981	0,89	1812	-	-
		254569386	C4R1Z9	PP7435_Chr2-0404	Thiol peroxidase that functions as a hydroperoxide receptor	6,18	18203	-0,46	-490	-	-	-	-	-	-
		254569682	C4R2E7	TRR1	hypothetical protein	5,10	34343	-	-	0,58	1068	-0,43	-1445	-	-
		254569734	C4R2H3	TSA1	Thioredoxin peroxidase, acts as both a ribosome-associated and free cytoplasmic antioxidant astoris	4,70	21418	0,67	1847	0,97	9287	-	-	-	-
		254569930	C4R2S1	CAT1	Catalase A, breaks down hydrogen peroxide in the peroxisomal matrix formed by acyl-CoA oxidase (Pox1	6,59	57810	-	-	-	-	1,04	1630	-	-
		254572796	C4R6V3	CAM1	Nuclear protein required for transcription of MXR1	6,10	47062	1,27	968	1,11	567	-	-	1,14	1409
		254573508	C4R7V9	PP7435_Chr4-0551	NADPH-dependent alpha-keto amide reductase	5,51	33673	-	-	-	-	-	-	2,00	620
		254574140	C4R8S5	PAS_chr4_0737	Mitochondrial NADH-cytochrome b5 reductase, involved in ergosterol biosynthesis	9,21	33553	-	-	-	-	-	-	0,85	4214
		254574362	C4R936	GLO1	Monomeric glyoxalase I	5,58	36628	-	-	-	-	-	-	2,00	1160
	Starvation - apoptosis	254566611	C4QY12	PP7435_Chr1-1080	Suppressor protein STM1	10,21	29685	2,00	455	2,00	772	-	-	-	-
	Detoxifying	254571583	C4R586	PAS_chr3_0674	D-lactate dehydrogenase, located in the mitochondrial matrix	5,80	59483	-	-	-	-	-0,82	-1496	-	-
	Osmoprotection	254571727	C4R5F8	PP7435_Chr3-0448	Aldose reductase involved in methylglyoxal, d-xylose and arabinose metabolism	5,90	36095	-	-	-	-	0,66	603	2,00	807
	Cell wall damage	254569896	C4R2Q4	PAS_chr2-2_0148	Cell wall protein that contains a putative GPI-attachment site	3,71	43204	-	-	-	-	-1,00	-6418	-0,93	-1321
Mitochondrial energy metabolism	Pyruvate dehydrogenase complex	254565157	C4QVY5	LAT1	Dihydrolipoamide acetyltransferase component (E2) of pyruvate dehydrogenase complex	6,60	50528	-	-	-	-	0,80	505	-	-
		254570112	C4R312	PP7435_Chr2-0049	Dihydrolipoamide dehydrogenase	6,30	52306	2,00	1261	1,20	1702	-	-	1,29	2354
	Krebs cycle	254564647	C4QV80	KGD2	Dihydrolipoyl transsuccinylase, component of the mitochondrial alpha-ketoglutarate dehydrogenase as	8,67	48038	-	-	-	-	0,76	818	0,99	404
		254564667	C4QV90	ACO1	Aconitase, required for the tricarboxylic acid (TCA) cycle and also independently required for mitoc	5,60	84478	2,00	1022	-	-	0,54	2422	-	-
		254564891	C4QVK2	DYS1	Deoxyhypusine synthase, catalyzes formation of deoxyhypusine	4,87	41461	-	-	-	-	-	-	0,54	416
		254565307	C4QW60	CIT1	hypothetical protein	8,56	51855	-	-	-	-	0,52	2914	-	-
		254567720	C4QZL6	sucA	Component of the mitochondrial alpha-ketoglutarate dehydrogenase complex, which catalyzes a key step	6,22	112682	-	-	-	-	0,94	2149	-	-
	Respiratory chain	254565263	C4QW38	COR1	Core subunit of the ubiquinol-cytochrome c reductase complex (bc1 complex)	5,00	47391	2,00	621	1,33	1208	-	-	-	-
	ATP synthase	254566933	C4QYH3	PP7435_Chr1-1240	Delta subunit of the central stalk of mitochondrial F1F0 ATP synthase	4,70	16963	-	-	2,00	807	-	-	-	-
		254568496	C4R0Q4	PAS_chr2-1_0451	hypothetical protein	4,60	13301	2,00	457	2,00	621	-	-	-	-
		254569858	C4R2N5	atpD	Beta subunit of the F1 sector of mitochondrial F1F0 ATP synthase	5,00	53974	0,62	525	1,10	3485	-	-	-	-
		254571387	C4R4Y8	atpA	Alpha subunit of the F1 sector of mitochondrial F1F0 ATP synthase	9,40	58713	-	-	-	-	0,46	489	-	-
Cellular trafficking		254567834	C4QZS3	KAR2	ATPase involved in protein import into the ER, also acts as a chaperone to mediate protein folding i	4,60	74150	0,88	1810	-0,46	-704	-	-	-	-
		254568888	C4R1A0	SAC6	Fimbrin, actin-bundling protein	5,12	70161	-	-	-	-	1,25	2010	-	-
		254570661	C4R3X5	SLB2	Primary component of eisosomes	4,70	33893	-	-	-	-	-0,84	-1462	-	-
		254571861	C4R5M5	NUC1	hypothetical protein	5,90	54808	-	-	-	-	2,00	455	-	-
		254572822	C4R6W6	PP7435_Chr4-0902	Minor isoform of tropomyosin, binds to and stabilizes actin cables and filaments	4,60	19018	-	-	-	-	-	-	0,75	774
Aminotransferases		254565475	C4QWE4	AAT1	Cytosolic aspartate aminotransferase	6,80	47734	2,00	621	0,57	705	-	-	-	-
		254571057	C4R4H3	CAR2	L-ornithine transaminase (OTAse)	5,90	47097	-	-	-	-	0,46	1958	-	-
		254573098	C4R7A4	PAS_chr4_0248	Mitochondrial branched-chain amino acid aminotransferase, homolog of murine ECA39	5,80	44670	-	-	2,00	455	-	-	-	-
Proteases (non-proteasomal)		254566719	C4QY66	PP7435_Chr1-1132	Putative metalloprotease	5,10	77665	-	-	-	-	-1,19	-757	-	-
		254572447	C4R6G8	36982	Vacuolar aspartyl protease (proteinase A)	4,49	44278	-	-	-2,00	-619	-0,80	-2011	-	-
		328352191	F2QT62	AAP1	Aminopeptidase N astoris CBS 7435]	4,50	23000	-	-	0,99	406	0,93	1324	0,66	945
Unclear		254564915	C4QVL4	GAS1	Beta-1,3-glucanosyltransferase, required for cell wall assembly	3,86	57236	-	-	-	-	-	-	-0,99	-403
		254565045	C4QVS9	PMA1	Plasma membrane H+-ATPase, pumps protons out of the cell	4,80	97797	-	-	-	-	-1,11	-5084	-0,82	-5975
		254566487	C4QXV0	PAS_chr1-4_0238	Mitochondrial protein involved in maintenance of the mitochondrial genome	9,10	15871	-	-	2,00	457	-	-	0,90	695
		254567754	C4QZN3	BMH1	hypothetical protein	4,65	28989	-	-	-	-	-	-	0,57	707
		254569162	C4R1N7	CMD1	Calmodulin	4,20	16850	2,00	1021	-	-	-0,54	-414	-	-
		254569544	C4R278	RKS1	Putative kinase	5,45	81518	-	-	-	-	-	-	2,00	457
		254570673	Q9P4D1	ACT1	hypothetical protein	5,16	41647	-	-	-	-	0,90	3483	0,57	707
		254570981	C4R4D5	PP7435_Chr3-0838	hypothetical protein	6,40	45175	-	-	0,42	1045	-0,71	-4275	1,04	2903
		254570983	C4R4D6	PP7435_Chr3-0837	Widely conserved NADPH oxidoreductase containing flavin mononucleotide (FMN)	6,15	45407	-	-	0,66	604	-1,03	-2535	2,00	1023
		254571485	C4R537	CRP1	Protein that binds to cruciform DNA structures	4,80	32192	-	-	-	-	-	-	0,54	415
		254571923	C4R5Q6	DAK	Dihydroxyacetone kinase, required for detoxification of dihydroxyacetone (DHA)	5,25	65254	-	-	-	-	-	-	0,84	576
		254572936	C4R723	uca	Urea amidolyase, contains both urea carboxylase and allophanate hydrolase activities	5,40	201006	-	-	-	-	2,00	456	-	-
		254573760	C4R885	PP7435_Chr4-0424	Putative protein of unknown function	5,48	36098	-	-	-	-	-	-	0,43	1445
		254573846	C4R8C8	PP7435_Chr4-0378	Glyoxylate reductase	6,02	38163	-	-	0,91	695	0,58	1069	-	-
		254573866	C4R8D8	PP7435_Chr4-0368	Acidic protein of the mitochondrial matrix involved in oxidative phosphorylation	4,43	28829	-	-	-	-	-0,98	-1549	-	-
		254574502	C4R9A6	PP7435_Chr4-0027	ATPase in ER, nuclear membrane and cytosol with homology to mammalian p97	4,68	91053	0,66	605	2,00	2467	-	-	0,59	2441
		328354583	F2R002	PP7435_Chr4-0827	Absent in melanoma 1 protein astoris CBS 7435]	7,18	65765	-	-	-	-	0,54	419	0,43	2113
Unassigned		254565273	C4QW43	PP7435_Chr1-0406	Putative protein proposed to be involved in the metabolism of purine and pyrimidine base analogues	4,96	24834	-	-	-	-	0,54	417	-	-
		254565299	C4QW56	PP7435_Chr1-0419	hypothetical protein	7,69	46294	-	-	-	-	-	-	0,55	960
		254567189	C4QYV1	SLB2	Primary component of eisosomes	4,90	35209	-	-	1,19	755	-0,61	-2023	-	-
		254567447	C4QZ80	PP7435_Chr1-1506	hypothetical protein	5,62	37893	-	-	-	-	1,00	1610	-	-
		254567798	C4QZQ5	EFT1	hypothetical protein	6,30	93392	0,73	1152	-	-	1,02	1730	-	-
		254568908	C4R1B0	PP7435_Chr2-0640	Putative protein of unknown function	6,40	21714	-	-	-	-	2,00	455	-	-
		254571875	C4R5N2	PP7435_Chr3-0372	Protein of unknown function involved in vacuolar protein sorting	6,50	94532	-	-	2,00	457	0,75	1206	-	-
		254574144	C4R8S7	PP7435_Chr4-0221	Putative mitochondrial ribosomal protein, has similarity to E. coli ribosomal protein S2 astoris GS	4,20	22782	-	-	-	-	-0,77	-819	-	-

Table S5. Differentially expressed mitochondrial proteins, as shown by the MAProMa comparison between the ATX3-trasformed- versus the control *P. pastoris* strain, and JD-transformed- versus JDC14A-transformed strain, grown at 37°C for 48 h. For each protein are reported: Molecular Function, GI Accession number, Uniprot entry, Gene name, Reference, pI, MW, Dave and DCI. Positive values for DAve and DCI indicate that a protein is more abundant in the ATX3- or in the JD-trasformed strain; negative in the control or in the JD-C14A-transformed strain, respectively. The bold borders indicate that the relevant DAve values are ≥ l0.4l; the DCI values ≥ l400l. Further details regarding DAve and DCI are in Materials and Methods. Proteins are grouped according to their function, in increasing GI Accession number.

Molecular Function		Accession	Uniprot Entry	Gene name	Reference	pI	MW	ATX3Q26 vs Ctrl		JD vs JDC14A	
								DAve	DCI	DAve	DCI
Mitochondrial energy metabolism	Pyruvate dehydrogenase complex	254565157	C4QVY5	LAT1	Dihydrolipoamide acetyltransferase component (E2) of pyruvate dehydrogenase complex	6,60	50528	0,80	505	2,00	115
		254567243	C4QYX8	PDHbeta1	E1 beta subunit of the pyruvate dehydrogenase (PDH) complex	4,91	39557	2,00	51	0,00	0
		254570112	C4R312	PP7435_Chr2-0049	Dihydrolipoamide dehydrogenase	6,31	52306	0,11	904	1,29	2354
		254571529	C4R559	PP7435_Chr3-0553	Fumarase, converts fumaric acid to L-malic acid in the TCA cycle	6,42	52570	0,00	0	0,31	716
	Krebs cycle	254564647	C4QV80	KGD2	Dihydrolipoyl transsuccinylase, component of the mitochondrial alpha-ketoglutarate dehydrogenase	8,67	48038	0,76	818	0,99	404
		254564667	C4QV90	ACO1	Aconitase, required for the tricarboxylic acid (TCA) cycle and also independently required for mitoc	5,63	84478	0,54	2422	0,29	2894
		254565307	C4QW60	CIT1	hypothetical protein	8,56	51855	0,52	2914	-0,02	-698
		254566517	C4QXW5	PP7435_Chr1-1033	Dihydrolipoamide dehydrogenase (E3)-binding protein (E3BP)	5,05	41704	0,85	266	0,61	132
		254567720	C4QZL6	sucA	Component of the mitochondrial alpha-ketoglutarate dehydrogenase complex, which catalyzes a key step	6,22	112682	0,94	2149	0,53	228
		254567788	C4QZQ0	IDH2	Subunit of mitochondrial NAD(+)-dependent isocitrate dehydrogenase, which catalyzes the oxidation of	8,05	39963	0,66	340	-0,25	-402
		254573818	C4R8B4	IDH1	Subunit of mitochondrial NAD(+)-dependent isocitrate dehydrogenase	7,60	39554	0,00	0	0,22	114
		254574312	C4R911	MDH3	Mitochondrial malate dehydrogenase, catalyzes interconversion of malate and oxaloacetate	5,20	36407	0,00	0	0,85	266
	Respiratory chain	254565263	C4QW38	COR1	Core subunit of the ubiquinol-cytochrome c reductase complex (bc1 complex)	5,00	47391	0,34	1817	-0,22	-114
		254565493	C4QWF3	ATP7	hypothetical protein	7,63	19659	0,40	64	-2,00	-52
		254566771	C4QY92	NUFM	hypothetical protein	4,92	15543	2,00	115	2,00	87
		254568302	C4R0F7	PAS_chr2-1_0361	Subunit IV of cytochrome c oxidase	7,82	18041	2,00	52	0,00	0
		254568308	C4R0G0	PP7435_Chr2-0937	Subunit VIa of cytochrome c oxidase, which is the terminal member of the mitochondrial inner membran	9,78	13989	2,00	51	0,00	0
		254570407	C4R3J8	PP7435_Chr3-1133	hypothetical protein	6,36	21157	2,00	116	0,00	0
		254573010	C4R760	PAS_chr4_0210	Major ADP/ATP carrier of the mitochondrial inner membrane	10,15	33190	2,00	51	0,00	0
		254573338	C4R7M4	PP7435_Chr4-0638	hypothetical protein	6,33	20762	2,00	115	0,00	0
		254573496	C4R7V3	PP7435_Chr4-0557	hypothetical protein	5,19	27180	2,00	52	0,00	0
		308152294	E1UWE2	ACP1	ACPM1 (SDAP) subunit of mitochondrial NADH:ubiquinone oxidoreductase (complex I)	4,82	15464	-2,00	-51	0,00	0
	ATP synthase	254565455	C4QWD4	ATP5	Subunit 5 of the stator stalk of mitochondrial F1F0 ATP synthase	9,59	21709	0,00	0	-0,22	-114
		254566933	C4QYH3	PP7435_Chr1-1240	Delta subunit of the central stalk of mitochondrial F1F0 ATP synthase	4,73	16963	0,24	698	0,08	208
		254566975	C4QYJ4	ATP3	Gamma subunit of the F1 sector of mitochondrial F1F0 ATP synthase	8,40	31603	0,66	153	0,40	64
		254568496	C4R0Q4	PAS_chr2-1_0451	hypothetical protein	4,62	13301	0,00	0	-0,65	-153
		254569858	C4R2N5	atpD	Beta subunit of the F1 sector of mitochondrial F1F0 ATP synthase	5,01	53974	0,07	493	0,07	1214
		254571387	C4R4Y8	atpA	Alpha subunit of the F1 sector of mitochondrial F1F0 ATP synthase	9,39	58713	0,46	489	0,12	1000
		254574006	C4R8K8	VMA7	Subunit F of the eight-subunit V1 peripheral membrane domain of vacuolar H+-ATPase (V-ATPase)	4,80	13846	0,00	0	-2,00	-51
		254574464	C4R987	PP7435_Chr4-0059	mitochondrial ATP synthase	7,50	10650	0,00	0	-0,66	-152

Table S6. Time course of glucose and ethanol concentrations (mM) in YPD medium at 37°C during growth of ATX3-transformed and control (transformed with empty vector) *Pichia* strains. Values are the mean of three determinations. Standard deviations never exceeded 5% of the mean value. For other details, see Materials and Methods.

**Growth time (h)**	**ATX3-transformed**		**Control**	
	**Glucose**	**Ethanol**	**Glucose**	**Ethanol**
**0**	115	b. d.	115	b. d.
**16**	89	42	110	b. d.
**24**	60	65	100	b. d.
**48**	16	119	110	b. d.

b. d.: below detection limit.
